# Polystyrene nanoplastic exposure induces excessive mitophagy by activating AMPK/ULK1 pathway in differentiated SH-SY5Y cells and dopaminergic neurons in vivo

**DOI:** 10.1186/s12989-023-00556-4

**Published:** 2023-11-22

**Authors:** Yuji Huang, Boxuan Liang, Zhiming Li, Yizhou Zhong, Bo Wang, Bingli Zhang, Jiaxin Du, Rongyi Ye, Hongyi Xian, Weicui Min, Xiliang Yan, Yanhong Deng, Yu Feng, Ruobing Bai, Bingchi Fan, Xingfen Yang, Zhenlie Huang

**Affiliations:** 1 NMPA Key Laboratory for Safety Evaluation of Cosmetics, Guangdong Provincial Key Laboratory of Tropical Disease Research, Department of Toxicology, School of Public Health, Southern Medical University, Guangzhou, 510515 People’s Republic of China; 2 School of Environmental Science and Engineering, Shandong University, Qingdao, 266237 People’s Republic of China; 3 Institute of Environmental Research at Greater Bay Area, Key Laboratory for Water Quality and Conservation of the Pearl River Delta, Ministry of Education, Guangzhou University, Guangzhou, 510006 People’s Republic of China; 4 NMPA Key Laboratory for Safety Evaluation of Cosmetics, Food Safety and Health Research Center, School of Public Health, Southern Medical University, Guangzhou, 510515 People’s Republic of China

**Keywords:** Microplastics and nanoplastics, Mitophagy, Mitochondrial dysfunction, Complex I, Neurotoxicity, Melatonin

## Abstract

**Background:**

Microplastics and nanoplastics (MNPs) are emerging environmental contaminants detected in human samples, and have raised concerns regarding their potential risks to human health, particularly neurotoxicity. This study aimed to investigate the deleterious effects of polystyrene nanoplastics (PS-NPs, 50 nm) and understand their mechanisms in inducing Parkinson's disease (PD)-like neurodegeneration, along with exploring preventive strategies.

**Methods:**

Following exposure to PS-NPs (0.5–500 μg/mL), we assessed cytotoxicity, mitochondrial integrity, ATP levels, and mitochondrial respiration in dopaminergic-differentiated SH-SY5Y cells. Molecular docking and dynamic simulations explored PS-NPs' interactions with mitochondrial complexes. We further probed mitophagy's pivotal role in PS-NP-induced mitochondrial damage and examined melatonin's ameliorative potential in vitro. We validated melatonin's intervention (intraperitoneal, 10 mg/kg/d) in C57BL/6 J mice exposed to 250 mg/kg/d of PS-NPs for 28 days.

**Results:**

In our in vitro experiments, we observed PS-NP accumulation in cells, including mitochondria, leading to cell toxicity and reduced viability. Notably, antioxidant treatment failed to fully rescue viability, suggesting reactive oxygen species (ROS)-independent cytotoxicity. PS-NPs caused significant mitochondrial damage, characterized by altered morphology, reduced mitochondrial membrane potential, and decreased ATP production. Subsequent investigations pointed to PS-NP-induced disruption of mitochondrial respiration, potentially through interference with complex I (CI), a concept supported by molecular docking studies highlighting the influence of PS-NPs on CI. Rescue experiments using an AMPK pathway inhibitor (compound C) and an autophagy inhibitor (3-methyladenine) revealed that excessive mitophagy was induced through AMPK/ULK1 pathway activation, worsening mitochondrial damage and subsequent cell death in differentiated SH-SY5Y cells. Notably, we identified melatonin as a potential protective agent, capable of alleviating PS-NP-induced mitochondrial dysfunction. Lastly, our in vivo experiments demonstrated that melatonin could mitigate dopaminergic neuron loss and motor impairments by restoring mitophagy regulation in mice.

**Conclusions:**

Our study demonstrated that PS-NPs disrupt mitochondrial function by affecting CI, leading to excessive mitophagy through the AMPK/ULK1 pathway, causing dopaminergic neuron death. Melatonin can counteract PS-NP-induced mitochondrial dysfunction and motor impairments by regulating mitochondrial autophagy. These findings offer novel insights into the MNP-induced PD-like neurodegenerative mechanisms, and highlight melatonin's protective potential in mitigating the MNP’s environmental risk.

**Supplementary Information:**

The online version contains supplementary material available at 10.1186/s12989-023-00556-4.

## Background

Microplastic and nanoplastic (MNP) pollution pose a significant environmental challenge, as they have been found in diverse ecosystems such as air [1], soil [2], oceans [3], rivers [4], and even human food systems [5]. A study has shown that measurable concentrations of MNPs in aquatic environments can reach 0.04 mg/L [6]. These particles have the ability to bioaccumulate in higher trophic levels through the food chain [7, 8] and have been detected in varying human bio-samples, including stool [9], lungs [10], blood (~ 1.6 μg/mL) [11], thrombi [12], placentas [13], and testes [14], raising concerns about their potential risks to human health. MNP exposure has been linked to intestinal toxicity [15, 16], pulmonary toxicity [17], hepatotoxicity [18], nephrotoxicity [19], cardiovascular toxicity [20, 21], and neurotoxicity [22]. Nanoplastics have demonstrated a higher capability to cross the blood–brain barrier (BBB) compared to microplastics [23, 24], thereby posing an increased risk to brain function. Given the brain's central role in coordinating bodily functions [25], it is crucial to understand the specific brain toxicity induced by nanoplastics.

Environmental factors have a noteworthy impact on brain dysfunction [26, 27], contributing to a range of central nervous system disorders, such as Parkinson's disease (PD) [28]. Recent research has highlighted the detrimental effects of ultrafine particles (≤ 100 nm) on the human brain [29]. Polystyrene, a highly common plastic, contributes to environmental pollution as it degrades, forming widespread polystyrene nanoplastics (PS-NPs) found abundantly in diverse environmental contexts [30]. PS-NPs have been shown to breach the BBB, leading to impaired brain function and altered neurobehaviors in mice [31, 32]. In our previous study, we identified the potential of PS-NPs to induce PD-like neurodegeneration in mouse brains through disrupted energy metabolism in dopaminergic neurons [33]. However, the specific mechanisms underlying PS-NP-induced energy metabolism disorder in dopaminergic neurons remain poorly understood, and effective preventive strategies for mitigating MNP's environmental risk are yet to be established. These knowledge gaps and the demand for comprehensive preventive strategies underscore the imperative for further research in PS-NP-induced neurodegeneration.

Mitochondria, the powerhouse of cellular energy metabolism, are implicated in numerous diseases, including neurodegenerative disorders like PD [34]. Mitochondrial dysfunction in PD involves impaired electron transport chain (ETC), altered morphology and dynamics, mutations in mitochondrial DNA, and disrupted calcium homeostasis [35]. Understanding the role of mitochondrial dysfunction in mediating energy metabolism disorder caused by PS-NPs in dopaminergic neurons requires further investigation. Mitophagy, a highly evolutionary conserved cellular process, maintains mitochondrial and metabolic homeostasis in neurons, ensuring energy supply, neuronal survival, and overall health [36]. Dysregulated mitophagy can disrupt cellular function and cause cytotoxicity [37, 38]. Given the scavenger role of mitophagy in mitochondrial dysfunction, we aimed to investigate its involvement in PS-NP-induced energy metabolism disorder in dopaminergic neurons.

Melatonin, synthesized by the pineal gland, exhibits diverse biological activities, including antioxidative, anti-inflammatory, and anti-apoptotic properties [39, 40]. It has demonstrated protective effects in conditions related to mitochondria, such as aging, neurodegenerative disorders, and various diseases [41]. While recent study suggests the protective potential of melatonin in PD [42], its role in preventing PS-NP-induced PD-like neurodegeneration remains unknown.

To address the knowledge gap, we utilized the differentiated SH-SY5Y neural cell model exposed to several doses of PS-NPs. We assessed cellular microstructure, mitochondrial integrity, ATP content, mitochondrial membrane potential (ΔΨm), and mitochondrial respiration. Molecular docking and dynamic simulations were employed to predict the interaction of PS-NPs with mitochondrial complexes. We investigated the mitophagy involvement in PS-NP-induced mitochondrial dysfunction in neuron cells and mice. Furthermore, we validated melatonin’s protective effects against PS-NP-induced PD-like neurodegeneration in mice. This study enhances understanding of the PS-NP-induced PD-like neurodegeneration mechanisms and provides insights for the prevention and risk assessment of MNP environmental exposure in humans.

## Results

### Characterization of nanoplastics

In our previous investigation, the utilization of scanning electron microscopy (SEM) revealed the spherical morphology of PS-NPs, with an average size of 50.7 nm for non-fluorescent (NF) PS-NPs and 50.2 nm for green fluorescent (GF) PS-NPs [15]. Another study's SEM showed the spherical morphology of polyethylene nanoplastics (PE-NPs), with an average size of 101.6 nm [43]. For the current study, we evaluated the fluid dynamic size, polymer dispersion index (PDI), and zeta potential of NF PS-NPs, GF PS-NPs, and PE-NPs in different solution systems using Zetasizer Nano ZS (Table 1). Our observations unveiled that NF PS-NPs exhibited monodispersity when suspended in double distilled water, as indicated by a high zeta potential, which is suggestive of minimal aggregation. Although the fluid dynamic size slightly increased in the MEM complete medium, all three types of nanoplastics maintained a narrow size distribution and exhibited a certain degree of stability.

**Table 1:** Physical characteristics of nanoplastics

Nanoplastic	Solution system	Dynamic light scattering		
		Fluid dynamic size (nm)	PDI	Zeta potential (mV)
NF PS-NPs	Double distilled water	51.89 ± 3.25	0.080 ± 0.041	-57.87 ± 3.36
NF PS-NPs	MEM complete medium	65.10 ± 9.98	0.760 ± 0.059	-10.60 ± 0.51
GF PS-NPs	MEM complete medium	52.81 ± 2.63	0.198 ± 0.001	-11.17 ± 0.88
PE-NPs	MEM complete medium	164.73 ± 19.01	0.661 ± 0.069	-5.16 ± 0.88

### PS-NPs induce differentiated SH-SY5Y cell death in reactive oxygen species (ROS)-independent manner

**Fig. 1:**
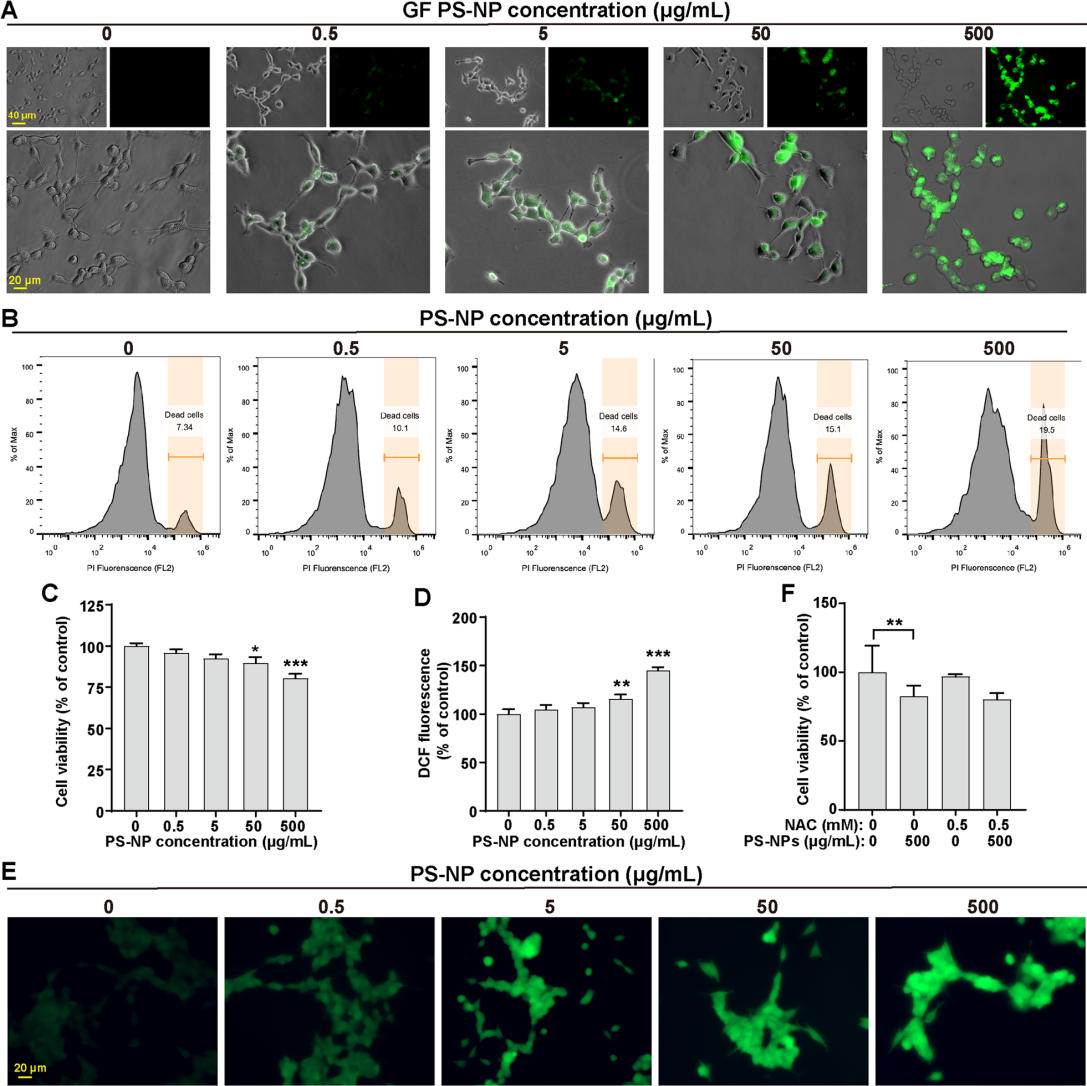
Cellular internalization and cytotoxicity of PS-NPs in differentiated SH-SY5Y cells. **A** GF PS-NPs distribution. **B** Flow cytometry analysis of PS-NP-induced cell death. **C** Cell viability after PS-NP exposure. **D**-**E** ROS levels post PS-NP exposure. **F** NAC effect on PS-NP-inhibited cell viability. Results are presented as mean ± SD (*n* = 3). *P < 0.05, **P < 0.01, ***P < 0.001, compared to the 0 μg/mL group

We investigated the neurotoxic potential of PS-NPs in differentiated SH-SY5Y cells. GF PS-NPs exhibited dose-dependent cytoplasmic accumulation (Fig. 1A), and transmission electron microscopy (TEM) analysis confirmed the uptake, even in mitochondria (Additional file 1: Fig. S1A-C). Propidium iodide (PI) staining revealed increased cell toxicity with higher PS-NP doses (Fig. 1B), and a cell counting kit-8 (CCK-8) assay showed reduced cell viability at 50 and 500 μg/mL (Fig. 1C). Four doses (0.5, 5, 50, 500 μg/mL) were selected for further investigation. Our focus also extended to investigating the role of oxidative stress in mediating PS-NP-induced cytotoxicity. After a 48-h PS-NP treatment, we observed a substantial increase in ROS levels within differentiated SH-SY5Y cells (Fig. 1D, E). Nevertheless, the introduction of the antioxidant N-acetylcysteine (NAC) at 0.5 mM, which effectively reduced ROS levels in PS-NP-exposed cells (Additional file 1: Fig. S2A, B), failed to rescue the PS-NP-inhibited cell viability (Fig. 1F). These results indicated that PS-NP-induced cytotoxicity in differentiated SH-SY5Y cells is not primarily due to ROS formation.

### PS-NPs induce mitochondrial dysfunction in differentiated SH-SY5Y cells

**Fig. 2:**
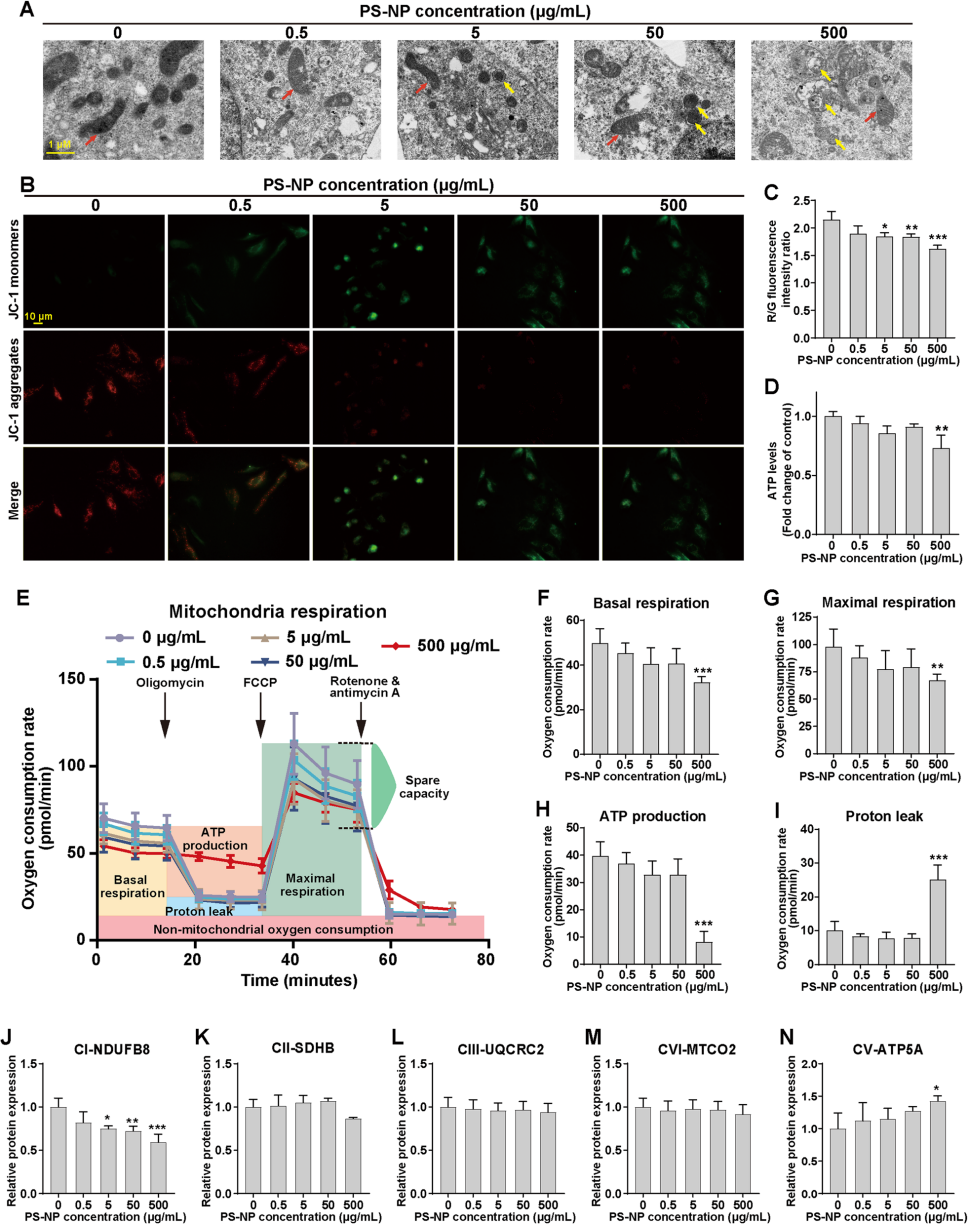
Impairment of mitochondria and mitochondrial stress by PS-NPs in differentiated SH-SY5Y cells. **A** Representative TEM images of cells after PS-NP exposure. Red arrows indicate mitochondria, and yellow arrows indicate mitophagosomes. Impact of PS-NPs on **B**, **C** ΔΨm and **D** cellular ATP levels. **E** Mitochondrial stress profiles. Relative changes in key parameters of mitochondrial function: **F** basal respiration, **G** maximal respiration, **H** mitochondrial ATP production, and **I** proton (H^+^) leakage. **J**–**N** The quantification of protein expression levels of ETC CI-NDUFB8, CII-SDHB, CIII-UQCRC2, CVI-MTCO2, and CV-ATP5A. Results are presented as mean ± SD (*n* = 3). *P < 0.05, **P < 0.01, ***P < 0.001, compared to the 0 μg/mL group

We have discovered that PS-NPs impact mitochondrial functionality in mouse brain dopaminergic neurons [33]. To validate these effects in vitro, we used differentiated SH-SY5Y cells. TEM imaging confirmed PS-NP-induced mitochondrial impairment, with morphological damage and the appearance of mitophagosomes (Fig. 2A). Additionally, we observed a dose-dependent reduction in ΔΨm (Fig. 2B, C) and decreased ATP levels (Fig. 2D), both of which are crucial indicators of mitochondrial dysfunction. Collectively, these findings indicated that PS-NPs can induce mitochondrial dysfunction in differentiated SH-SY5Y cells.

### PS-NPs disrupt mitochondrial respiration in differentiated SH-SY5Y cells

To comprehensively assess the influence of PS-NP exposure on mitochondrial function, we conducted a thorough investigation into the ETC function in the oxidative phosphorylation (OXPHOS). Our results revealed dose-dependent alterations in the mitochondrial stress profiles of differentiated SH-SY5Y cells (Fig. 2E). Basal and maximal respiration were notably reduced at a dose of 500 μg/mL PS-NP (Fig. 2F, G). The impact on mitochondrial ATP production, a fundamental indicator of OXPHOS, was even more pronounced, showcasing a substantial decline (Fig. 2H). Furthermore, our analysis unveiled the presence of severe proton leakage within the 500 μg/mL PS-NP group, indicative of severe functional impairment of complex I (CI) (Fig. 2I). To further unravel the underlying mechanisms, we examined the expression levels of critical subunits constituting the five mitochondrial complexes in mitochondrial respiration. Protein expression analysis revealed decreased CI-NDUFB8 levels with escalating PS-NP doses (Additional file 1: Fig. S3 and Fig. 2J). CII-SDHB, CIII-UQCRC2 and CVI-MTCO2 exhibited no discernible changes (Additional file 1: Fig. S3 and Fig. 2K–M). Increased CV-ATP5A expression indicated a potential stress response to the PS-NP-triggered decline in cellular ATP content (Additional file 1: Fig. S3 and Fig. 2N). These findings suggested that PS-NP exposure disrupts mitochondrial respiration by probably affecting CI, leading to disrupted ATP synthesis and mitochondrial injury in differentiated SH-SY5Y cells.

### Molecular docking identifies CI

To study the direct influence of PS-NPs on crucial mitochondrial complexes in OXPHOS and deeply analyze the intrinsic molecular mechanism, we conducted molecular docking and molecular dynamics (MD) simulations. Due to computational limitations in simulating actual 50 nm PS-NP, we employed a model of PS (n10) consisting of 10 repetitive PS chains to represent the PS-NPs. Initially, we selected the structures of five mitochondrial complexes involved in the OXPHOS and performed molecular docking with PS-NPs (Additional file 1: Fig. S4A-E). Using the affinity dG score, we found that PS-NPs had the highest affinity for CI (Additional file 1: Fig. S4F). Moreover, when compared with other types of nanoplastics, such as polypropylene (PP), polyethylene (PE), polyvinyl chloride (PVC), the affinity score between PS-NPs and CI remained the highest (Additional file 1: Fig. S5A-C, E). We also conducted cell experiments with PE-NPs and observed that a high dose of PE-NPs at 500 μg/mL did not result in a significant reduction in cell viability, ΔΨm, or ATP production (Additional file 1: Fig. S6A-D). Additionally, we compared CI's binding ability to larger PS (n20) composed of 20 repeating PS chains and found no significant difference (Additional file 1: Fig. S5D-E).

**Fig. 3:**
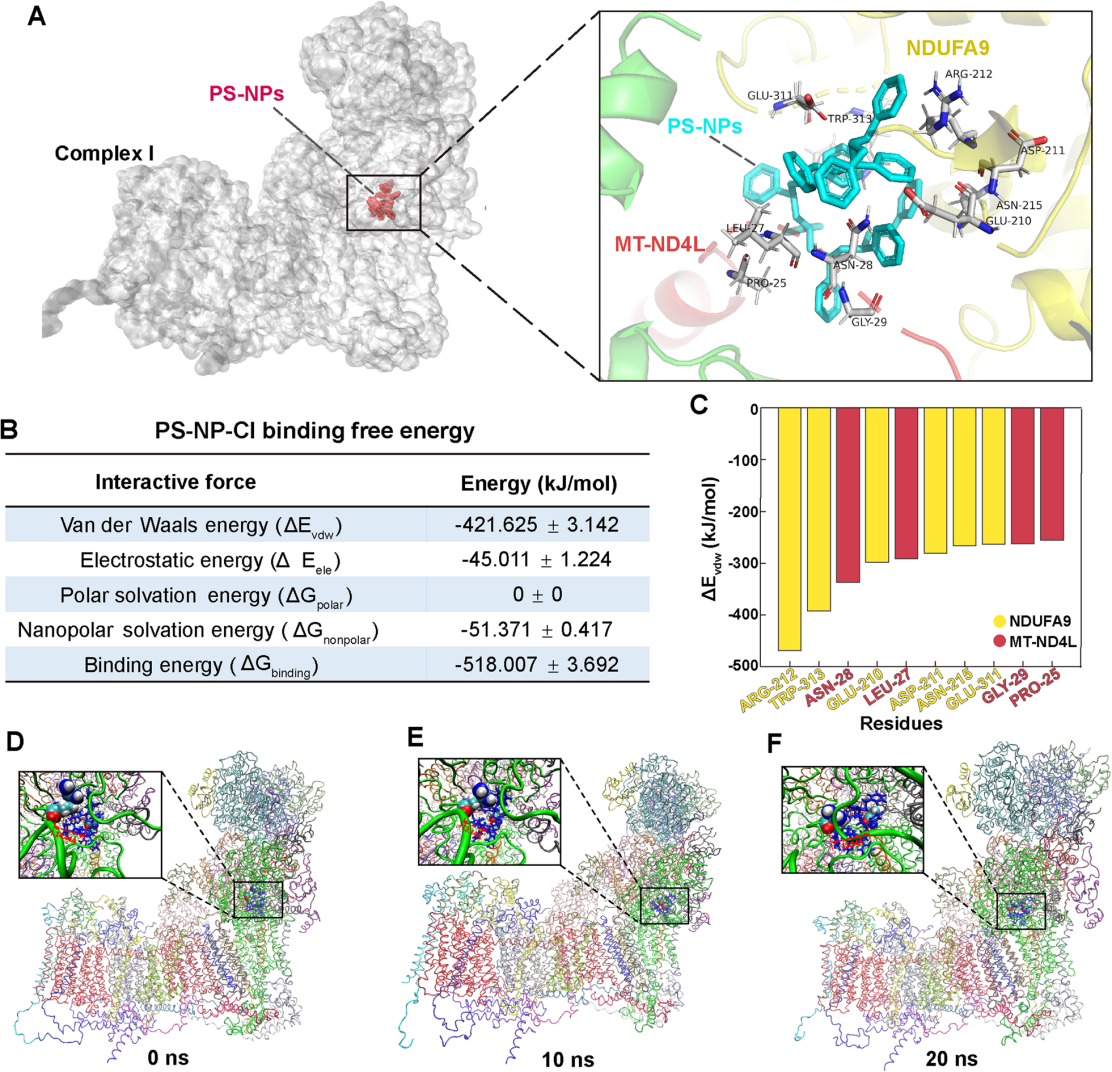
Molecular docking and MD simulation showing interaction between PS-NPs and CI. **A** Molecular docking model illustrating the binding of PS-NPs to CI. Detailed interaction view (enlarged panel). **B** Binding free energy between PS-NPs and CI. **C** Van der Waals interactions between specific residues of CI and PS-NPs. **D**-**F** Conformational changes of the PS-NP- CI complex in MD simulation

The docked molecular conformation was preserved, and its molecular dynamics were simulated to gain insight into the dynamic process of PS-NP-CI interaction. The results identified NDUFA9 and MT-ND4L as the main binding partners (Fig. 3A). To explore the predominant factor driving the binding of PS-NPs to CI, we decomposed the binding free energy into vacuum potential energy, encompassing electrostatic and van der Waals interactions, as well as solvation free energy, including polar and non-polar solvation interactions. As shown in Fig. 3B, the binding free energy (-518.007 ± 3.692) indicated that PS-NPs tightly binds to CI. Among the various binding energies, van der Waals energy (ΔEvdw) was relatively large, suggesting that van der Waals interactions were predominantly influential in the binding mechanism. Figure 3C lists the residues that primarily contributed to the ΔEvdw. Previous reports have highlighted the crucial role of NDUFA9 in stabilizing the interface between the matrix arm and the membrane arm of CI and its significance as a binding site for ubiquinone-10 (Q10) [44]. Based on this, we hypothesized that PS-NP exposure might induce conformational changes in CI, thereby inhibiting its active site. To test this, we measured the change in distance between two residues at the binding site, and the analysis at 0, 10, and 20 ns confirmed PS-NP-induced conformational changes in the Q10-binding domain, leading to reduced CI activity (Fig. 3D–F). These findings provided evidence that PS-NPs can induce conformational alterations in the 3D architecture of the Q10-binding domain, ultimately resulting in the attenuation of CI activity.

### PS-NPs cause mitophagy in differentiated SH-SY5Y cells

**Fig. 4:**
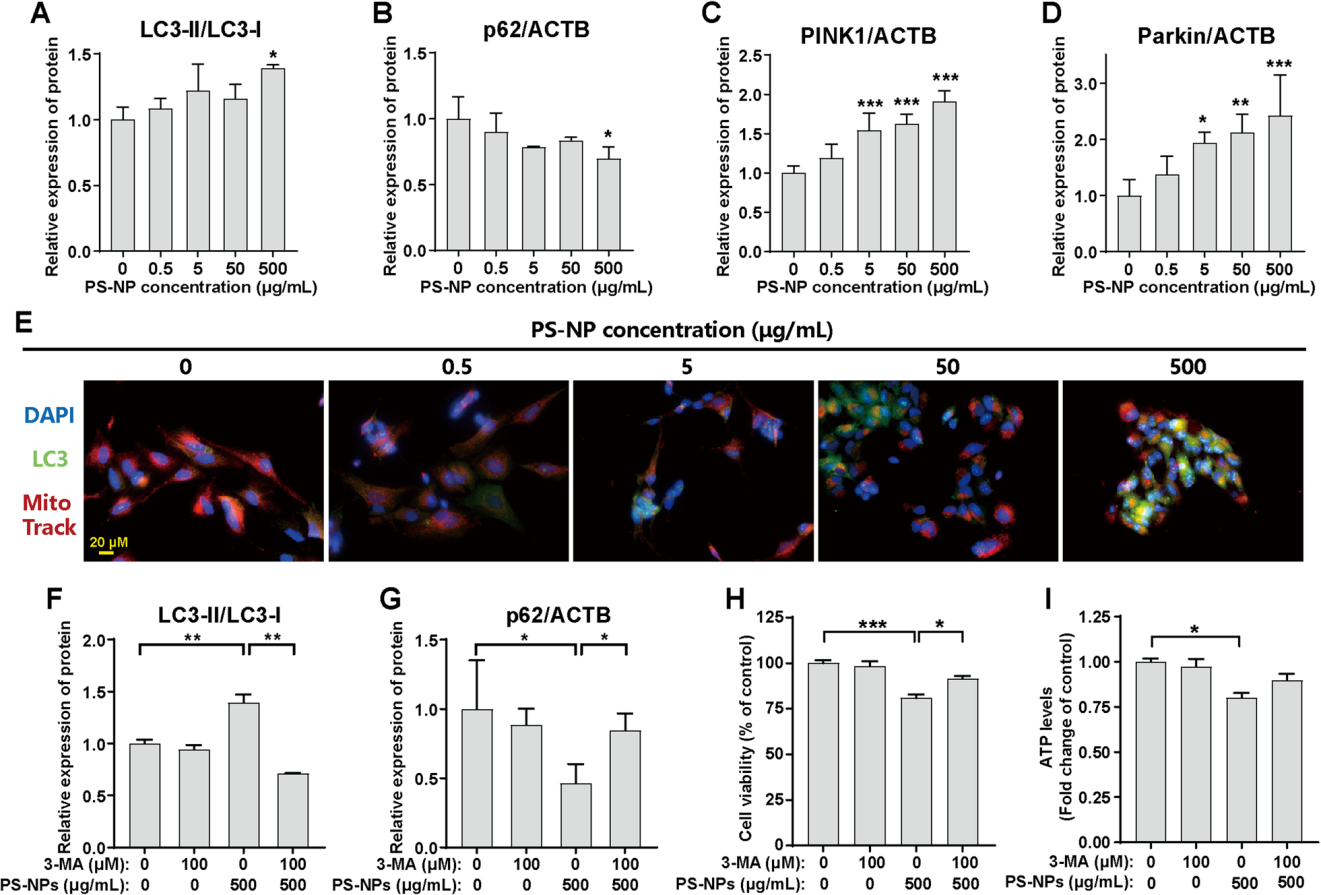
PS-NP-induced aberrant mitophagy mediated by PINK1/Parkin in differentiated SH-SY5Y cells. **A**-**D** The quantification of mitophagy-related proteins, including LC3-II/LC3-I, p62, PINK1, and Parkin. **E** Representative IF images showing colocalization of LC3 (green) and Mito tracker (red). **F**-**I** Differentiated SH-SY5Y cells were exposed to 500 μg/mL PS-NPs with or without 3-MA (100 μM) for 48 h. The quantification of protein expression levels of (**F**) LC3-II/LC3-I and (**G**) p62. (**H**) Cell viability and (**I**) ATP levels. Results are presented as mean ± SD (*n* = 3). * *P* < 0.05, ** *P* < 0.01, *** *P* < 0.001, compared to the 0 μg/mL group or indicative group

We subsequently investigated the implications of impaired mitophagy in PS-NP-triggered mitochondrial dysfunction. Increasing PS-NP exposure upregulated microtubule-associated protein 1 light chain 3 (LC3)-II protein (Additional file 1: Fig. S7A and Fig. 4A) and downregulated p62 proteins (Additional file 1: Fig. S7A and Fig. 4B), indicating enhanced autophagy and lysosomal degradation. Key mitophagy-related molecules, PINK1 and Parkin, were also upregulated (Additional file 1: Fig. S7A and Fig. 4C, D), even at the dose of 5 μg/mL PS-NPs. Immunofluorescence (IF) staining showed extensive colocalization of LC3 and the Mito tracker in PS-NP-exposed cells (Fig. 4E), and TEM analysis confirmed mitophagosomes (Fig. 2A). Furthermore, we assessed the influence of mitophagy on PS-NP-induced toxicity by assessing the rescue effects of autophagy inhibitors. Autophagy inhibitor 3-methyladenine (3-MA) reversed PS-NP-activated mitophagy (Additional file 1: Fig. S7B and Fig. 4F, G) and restored cell viability (Fig. 4H), but not ATP levels (Fig. 4I), indicating persistent mitochondrial dysfunction. Mitochondrial aberrant quality control, including impaired mitophagy, may further contribute to PS-NP-induced mitochondrial damage in differentiated SH-SY5Y cells.

### PS-NPs activate mitophagy via AMPK/ULK1 pathway in differentiated SH-SY5Y cells

**Fig. 5:**
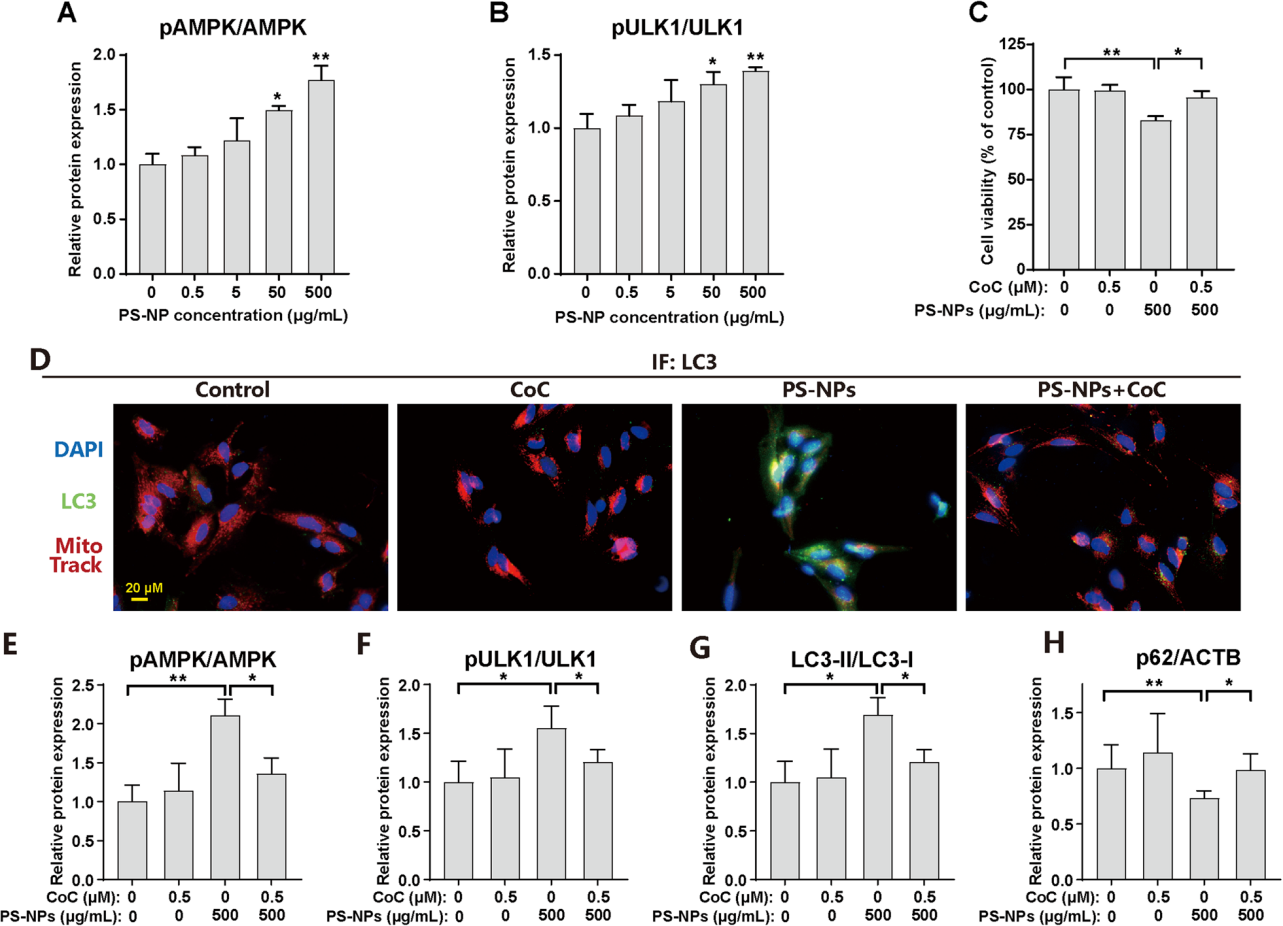
Regulation of AMPK/ULK1 signaling pathway in PS-NP-activated mitophagy in differentiated SH-SY5Y cells. **A**, **B** The quantification of protein expression levels of the AMPK/ULK1 pathway, including phosphorylated and total AMPK and ULK1. **C**-**H** Differentiated SH-SY5Y cells were exposed to 500 μg/mL PS-NPs with or without CoC (0.5 μM) for 48 h. **C** Cell viability, and **D** representative IF images showing colocalization of LC3 (green) and Mito tracker (red). The quantification of protein expression levels of **E** pAMPK/AMPK, **F** pULK1/ULK1, **G** LC3-II/LC3-I, and **H** p62. Results are presented as mean ± SD (*n* = 3). * *P* < 0.05, ** *P* < 0.01, compared to the 0 μg/mL group or indicative group

As previously indicated, the disruption of the mitochondrial ETC leads to an imbalance in cellular energy metabolism, resulting in decreased ATP content. Thus, we investigated the involvement of AMP-activated protein kinase (AMPK), a regulator of metabolic homeostasis, in initiating mitophagy. Dose-dependent increases in phosphorylated AMPK (pAMPK) and phosphorylated ULK1 (pULK1), key proteins in the AMPK/ULK1 pathway, were observed (Additional file 1: Fig. S8A and Fig. 5A, B), indicating activation of this cascade in PS-NP-exposed SH-SY5Y cells. To further explore the role of AMPK, we used compound C (CoC), an AMPK pathway inhibitor. CoC treatment significantly enhanced cellular survival compared to cells exposed to 500 μg/mL PS-NPs alone (Fig. 5C), suggesting that PS-NP-induced damage is linked to AMPK pathway activation. CoC also reduced PS-NP-induced mitophagy (Fig. 5D) and altered protein levels of pAMPK, pULK1, LC3-II, and p62 (Additional file 1: Fig. S8B and Fig. 5E–H). These findings suggested that PS-NPs induce excessive mitophagy via the AMPK signaling pathway, resulting in cellular damage.

### Melatonin reverses PS-NP-induced mitophagy in differentiated SH-SY5Y cells

**Fig. 6:**
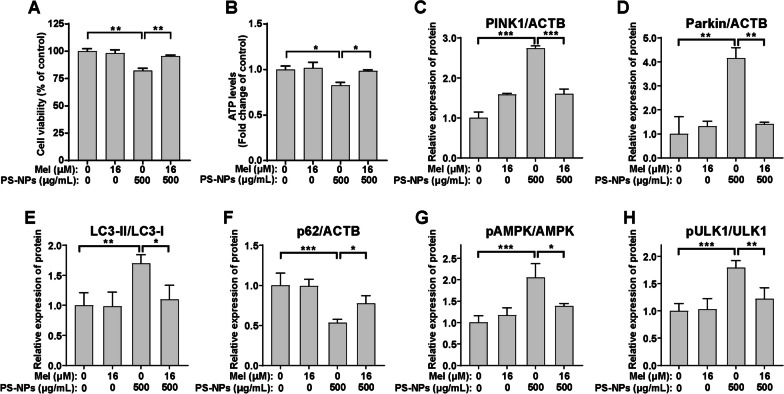
Protective effects of melatonin on PS-NP-induced mitochondrial dysfunction and excessive mitophagy in differentiated SH-SY5Y cells. Differentiated SH-SY5Y cells were exposed to 500 μg/mL PS-NPs with or without Mel (16 μM) for 48 h. **A** Cell viability and **B** ATP levels. The quantification of protein expression levels of **C** PINK1, **D** Parkin, **E** LC3-II/LC3-I, **F** p62, **G** pAMPK/AMPK, and **H** pULK1/ULK1. Results are presented as mean ± SD (*n* = 3). * *P* < 0.05, ** *P* < 0.01, *** *P* < 0.001, compared to the 0 μg/mL group or indicative group. Note: Mel, melatonin

Melatonin, a hormone known for regulating mitochondrial homeostasis, has emerged as a potential protective agent [45]. We investigated its impact on neuronal mitophagy and mitochondrial function after PS-NP exposure. Notably, melatonin (16 μM) restored mitochondrial dysfunction induced by PS-NPs and rescued the viability in differentiated SH-SY5Y cells (Fig. 6A, B). We further examined its effects on ROS levels and determined that it did not significantly reduce ROS levels (Additional file 1: Fig. S9), effectively excluding a primary intervention mechanism involving ROS level reduction. Additionally, melatonin effectively counteracted PS-NP-induced disruption of mitophagy-related proteins (Additional file 1: Fig. S10 and Fig. 6C–F). We explored melatonin's mechanism of action and found that it attenuates the activation of the AMPK/ULK1 pathway triggered by PS-NPs, downregulating pAMPK and pULK1 levels (Additional file 1: Fig. S10 and Fig. 6G, H). These findings collectively indicated that melatonin can ameliorate PS-NP-induced mitophagy disturbances via the AMPK/ULK1 pathway in differentiated SH-SY5Y cells.

### Melatonin reverses PS-NP-induced dopaminergic neuron mitophagy in vivo

**Fig. 7:**
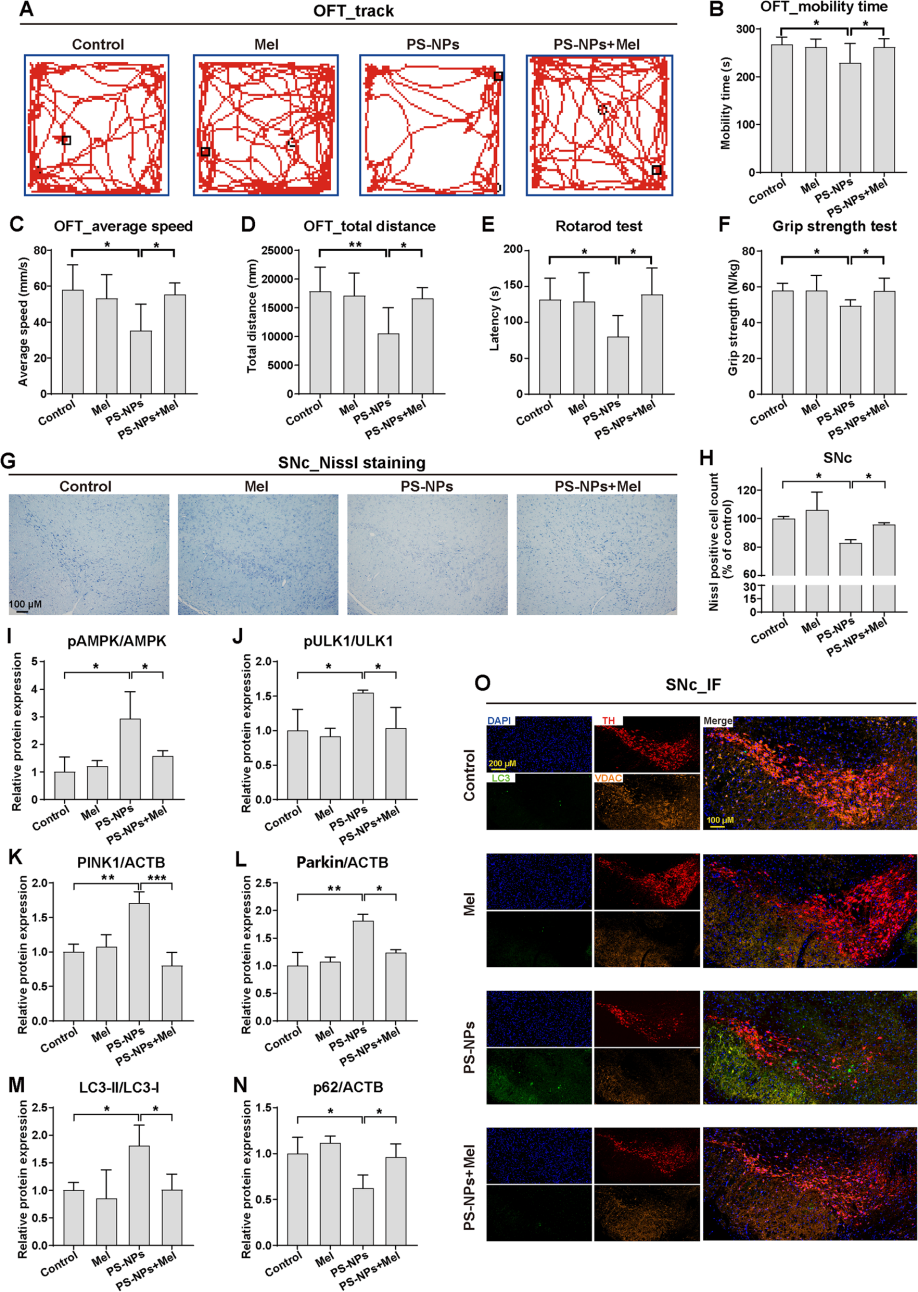
Protective effects of melatonin on PS-NP-induced motor and coordination impairments in mice by mitigating dopaminergic neurons' mitophagy. 250 mg/kg/day PS-NPs and 10 mg/kg/day melatonin were applied during 28-day exposure. **A** Activity trajectory, **B** mobility time, **C** average speed, and **D** total distance in the open field test (OFT). **E** Latency time in the rotarod test, and **F** grip strength in the grip strength test. **G**-**H** Representative images of Nissl staining and quantitative analyses in SNc neurons. Detection and quantification of protein expression levels of **I** pAMPK/AMPK, **J** pULK1/ULK1, **K** PINK1, **L** Parkin, **M** LC3-II/LC3-I, and **N** p62 in mouse midbrain. **O** Representative image of triple IF for LC3 (green, labeling autophagosomes), TH (red, labeling dopaminergic neurons), VDAC (orange, labeling mitochondria) and their merged images with DAPI (blue, labeling cell nucleus) in the SNc. *n* = 10 per group for neurobehavioral tests, and *n* = 5 per group for other experiments. Results are presented as mean ± SD. *P < 0.05, **P < 0.01, ***P < 0.001, compared to the indicative group. Note: Mel, melatonin

To validate our findings in vitro, we conducted an animal study. Melatonin supplementation significantly alleviated PS-NP-induced PD-like neurodegeneration, manifested by the amelioration of motor and coordination disorders, as evidenced by restored locomotor activity levels (Fig. 7A–D), increased latency time for falling off the rod (Fig. 7E), and improved grip strength (Fig. 7F). It also mitigated the loss of neurons in the nigra pars compacta (SNc) and striatum caused by PS-NPs, as seen in Nissl staining (Fig. 7G, H and Additional file 1: Fig. S12A, B). Melatonin attenuated the activation of the AMPK/ULK1 signaling pathway in the SNc and striatum following PS-NP exposure (Fig. 7I–J, Additional file 1: Fig. S11 and Fig. S12C-E). It restored aberrant mitophagy (Fig. 7K–N, Additional file 1: Fig. S11 and Fig. S12C, F-I) and mitigated mitochondrial loss in dopaminergic neurons in the SNc and striatum induced by PS-NPs (Fig. 7O and Additional file 1: Fig. S12J). Overall, our results demonstrated that melatonin prevents PS-NP-induced motor and coordination impairments in mice by mitigating dopaminergic neurons' mitophagy via the regulation of the AMPK/ULK1 pathway.

## Discussion

**Fig. 8:**
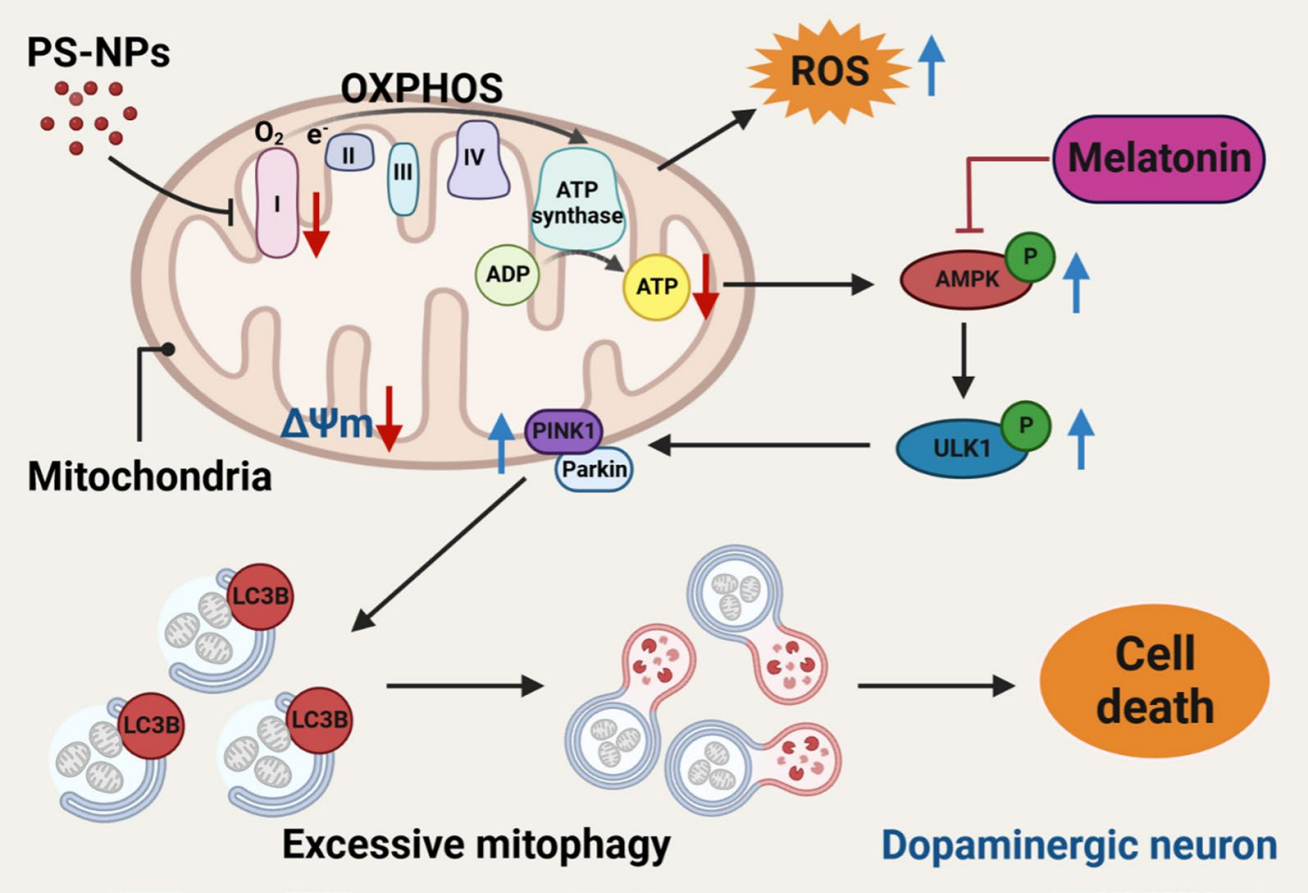
Graphical abstract. Dopaminergic neurons exposure to PS-NPs induces mitochondrial dysfunction and triggers excessive mitophagy, which can be restored by melatonin treatment

In our previous work, we observed PS-NP-induced PD-like neurodegeneration in the SNc and striatum of mouse brains, linked to disrupted mitochondrial energy metabolism [15, 33]. To elucidate the precise effects, we utilized dopaminergic-differentiated SH-SY5Y cells. PS-NPs may directly impact mitochondrial OXPHOS, particularly CI, leading to reduced ATP synthesis. This activated the AMPK/ULK1 pathway, causing excessive mitophagy through PINK1/Parkin regulation, intensifying mitochondrial damage and neuronal cell death. Notably, melatonin showed promise in mitigating PS-NP-induced mitochondrial dysfunction and motor impairments by restoring mitophagy regulation (Fig. 8).

Mitochondria are essential for intracellular processes, including ATP production, calcium regulation, iron metabolism, immunity, and redox reactions [46]. Mitochondrial dysfunction disrupts cellular homeostasis, and PS-NPs have been implicated in mitochondrial damage [47–50]. Our study revealed PS-NP-induced mitochondrial dysfunction, reducing ΔΨm, oxygen consumption rate and ATP synthesis. These changes are also found in other tissue cells, such as human liver cells and lung cells [47]. The mechanisms underlying PS-NP-induced mitochondrial damage remain unclear. In our animal experiment, we have observed impaired OXPHOS in mouse brain dopaminergic neurons [33]. Other studies also reported NP exposure interfering with OXPHOS in different organisms [19, 51, 52]. Therefore, we hypothesized that PS-NPs directly affect mitochondrial OXPHOS, resulting in mitochondrial damage. Increasing PS-NP exposure led to reduced mitochondrial OXPHOS, particularly CI. Mitochondrial CI serves as the initial site for electron entry into the respiratory chain, facilitating the transfer of electrons from nicotinamide adenine dinucleotide (NADH) to Q10 and proton translocation across the inner mitochondrial membrane for ATP synthesis [53, 54]. Inhibition of CI has been linked to mitochondrial permeability transition pore (mPTP) opening and depolarization of ΔΨm [55], and dopaminergic neuron death in PD [56]. Therefore, we speculated that PS-NPs may directly bind to the CI's active site, thereby obstructing electron transfer.

To validate the PS-NPs' interaction with mitochondrial complexes, we performed molecular docking, a commonly used method for assessing the direct impact of plastic particles on biomacromolecules. Previous studies have employed this approach to evaluate esterase activity [57], cytochrome P450 17A1 interactions [58], and neurotransmitter receptor effects [59]. Our findings demonstrated significant interactions between PS-NPs and CI, with preferential binding to subunits NDUFA9 and MT-ND4L, suggesting potential specific binding effects. Notably, the NDUFA9 subunit is a crucial binding site for Q10, a key independent component of ETC [44]. The competitive binding of PS-NPs at the active site may inhibit electron transfer function within CI. In addition, we compared molecular docking simulation results of PS-NPs with nanoplastics composed of other materials, such as PP, PE, and PVC. This comparison underscored the specific binding ability of PS-NPs to CI. Cell experiments indicated that the impact of PE-NPs on mitochondrial function is smaller than that of PS-NPs, further supporting this observation. Although the size of the simulated PS-NPs may not be directly comparable to the experimental size due to computational limitations, our additional simulations showed that larger PS-NPs also exhibit high binding capacity. Thus, larger PS-NPs are expected to interact with CI in a similar manner. In other studies, different-sized nanoplastics have displayed similar behavior when simulating their interactions with other molecules [60]. Collectively, although our in silico data strongly support the hypothesis that PS-NPs can inhibit mitochondrial CI, further experimental investigations are necessary to conclusively establish a direct causal relationship.

Mitophagy, the selective degradation of damaged mitochondria, can promote cell survival in response to mitochondrial dysfunction. While it can promote cell survival by removing damaged mitochondria [61, 62], excessive mitophagy can degrade functional mitochondria and contribute to cell death [55, 63, 64]. Our study demonstrated that PS-NPs activates the PINK1/Parkin-mediated mitophagy both in vivo and in vitro. To dissect the nature of PS-NP-induced mitophagy, we used the autophagy inhibitor 3-MA. Our findings underscore that suppressing autophagy partially restores cell viability, indicating excessive mitophagy may occur after mitochondrial dysfunction onset, leading to neuronal death.

AMPK regulates cellular energy balance and mitophagy [65]. In ATP-depleted conditions, AMPK becomes activated and triggers the AMPK/ULK1 axis, which promotes selective degradation of dysfunctional mitochondria through mitophagy [66, 67]. In alignment with prior investigations, our findings showed that PS-NP-induced mitochondrial dysfunction and energy deficiency activate the AMPK/ULK1 pathway, resulting in excessive mitophagy. This was supported by CoC experiments, which inhibited excessive mitophagy. However, manipulating mitophagy alone partially restored cell viability but not ATP synthesis. Our results elucidated the sequence of events following PS-NP exposure: disrupted OXPHOS, persistent low ATP levels, and subsequent activation of the AMPK/ULK1 pathway driving excessive mitochondrial autophagy.

Considering the inevitable exposure of humans to MNPs [68], their neurotoxicity is a significant concern. Therefore, identifying neuroprotective agents to mitigate PS-NP-related risks is crucial. Our study emphasized the central role of mitochondrial complex dysfunction in PS-NP-induced neurotoxicity. Remarkably, melatonin, with its amphiphilic nature and strong affinity for mitochondria, emerges as a promising candidate. Melatonin easily penetrates biofilms and accumulates in mitochondria, exerting beneficial effects [45, 69]. Prior researches have shown that melatonin enhances the activity of mitochondrial respiratory complexes I and IV, resulting in fostering heightened ATP generation [70, 71]. Moreover, melatonin activates protective signaling pathways in mitochondria, making it an ideal candidate to mitigate the neurotoxic effects of PS-NPs. Importantly, the dose and timing of melatonin administration have been previously validated for both efficacy and safety, with no significant adverse effects observed in healthy animals [69, 72]. Therefore, we adopted this established treatment protocol and administered melatonin to PS-NP-exposed mice during the daytime. Our findings demonstrated that melatonin treatment effectively mitigates neuronal loss in the striatum and SNc and alleviates motor and coordination impairments induced by PS-NPs in mice. While melatonin is often employed for its antioxidant properties [73], our in vitro experiments revealed that the melatonin intervention had a minimal impact on ROS levels. Additionally, the classic antioxidant inhibitor NAC treatment failed to mitigate the cellular damage induced by PS-NPs in our in vitro experiments. Thus, it appears that the primary mode of action of melatonin in this study is not related to its antioxidant properties. Furthermore, melatonin treatment attenuated excessive mitophagy by inhibiting the AMPK/ULK1 pathway, underscoring its crucial role in neuronal mitophagy regulation. Several lines of evidence have demonstrated the neuroprotective role of melatonin in modulating mitophagy in reaction to environmental stimuli [74, 75]. In addition to its effects on mitochondrial autophagy, melatonin stimulates the process of mitochondrial biogenesis [76, 77] and regulates fission/fusion dynamics [78], suggesting its reparative effects on mitochondria. Thus, melatonin shows promising neuroprotective effects in mitigating PS-NP-induced neurotoxicity by repairing mitochondrial function and inhibiting excessive mitophagy.

To comprehensively study PS-NP toxic mechanisms in vitro, we used doses ranging from 0.5–500 μg/mL, covering environmentally relevant and human exposure levels. For differentiated SH-SY5Y cells, we used 0.5, 5, and 50 μg/mL based on human blood MNP levels (~ 1.6 μg/mL) [11]. We also set a high-dose group of 500 μg/mL with a tenfold difference for dose-dependent hazard identification. In C57BL/6 J mice, oral administration of 500 mg/kg of PS-NPs for 24 h results in blood and brain concentrations of 33.96 μg/mL and 29.27 μg/g, respectively [15]. In *ApoE*^−/−^ mice, the blood PS-NP levels of the three exposed groups are 0.17, 0.51, and 3.37 μg/mL, respectively, after 24 h of oral gavage with PS-NPs at doses of 2.5, 25, and 250 mg/kg [79]. Our previous studies indicate that PS-NPs exhibit limited biodistribution in the blood and entry into the brain [15, 79]. Therefore, although a dose of 250 mg/kg of PS-NPs was used in the present 28-day animal study, the resulting PS-NP level in the mouse blood remained relevant to the MNP level found in human blood (~ 1.6 μg/mL). Notably, the doses of nanoplastics, established according to the mass concentration of microplastics exposed to the human body, may result in a considerable quantity of nanoplastics due to differences in particle size. Nevertheless, considering the significant increase in particle numbers following the further breakdown of microplastics [80, 81], and the enhanced likelihood of nanoplastics entering tissues due to their minute size [82], it's important to recognize these aspects. Although accurate data on human exposure to nanoplastics are lacking, it is reasonable to speculate that the internal exposure level of nanoplastics could surpass that of microplastics. Furthermore, our animal experiment focused on melatonin's efficacy in mitigating PS-NP-induced neurotoxicity, using a high-dose approach to induce consistent PD-like neurotoxicity by PS-NPs [33]. This design assessed melatonin's benefits in an established neurotoxic model.

However, several limitations should be acknowledged in our study. Firstly, our investigation only focused on a specific size of PS-NPs for exposure, and further research is warranted to delve into the effects of other types of MNPs and their interactions with different pollutants to obtain a more comprehensive understanding of their impact on human health. Secondly, the exposure doses of PS-NPs in this study were primarily determined based on the mass concentration of microplastics found in the blood, which is relevant to human exposure. However, it is important to acknowledge that differences between mass concentration and number concentration of MNPs may introduce certain limitations to this comparison. Furthermore, while differentiated SH-SY5Y cells are commonly used as dopaminergic neuron models, the inclusion of additional *ex vitro* models, such as primary dopaminergic neurons, would enhance and corroborate the credibility of our present results. Lastly, although we have provided initial evidence for the protective impact of melatonin against PS-NP-induced neurotoxicity, further studies are required to fully elucidate the underlying molecular mechanisms involved.

## Conclusions

Our study demonstrated that PS-NPs disrupt mitochondrial function by affecting CI, leading to reduced ATP synthesis and excessive mitophagy through the AMPK/ULK1 pathway. This mitochondrial dysfunction causes dopaminergic neuron death. Importantly, melatonin can counteract PS-NP-induced mitochondrial dysfunction and motor impairments by regulating mitochondrial autophagy. These findings enhance our understanding of the PS-NP-induced PD-like neurodegeneration mechanisms and highlight melatonin's protective potential in mitigating the MNP’s environmental risk.

## Methods

### Nanoplastics and reagents

NF and GF pristine PS-NPs with a diameter of 50 nm and concentrations of 10% and 4% (w/v), respectively, were obtained from Magsphere (Pasadena, CA, USA). The PS-NPs had a density of 1.05 g/cm^3^. It's important to note that, in this study, only experiments using fluorescent nanoplastics are explicitly denoted as GF PS-NPs, while the term PS-NPs generally refers to non-fluorescent nanoplastics. PE-NPs (nominal size: 100 nm) were generously provided by Professor Dayong Wang from the Medical School of Southeast University, Nanjing, China. The chemical reagents NAC, 3-MA, CoC, melatonin, retinoic acid (RA) and phorbol 12-myristate 13-acetate (PMA) were purchased from MedChemExpress (MCE, Shanghai, China). The 2′,7′-dichlorodihydrofluorescein diacetate (DCFH-DA), enhanced ATP assay kits, PI dye, and enhanced mitochondrial membrane potential assay kit with 5,5′,6,6′-tetrachloro-1,1′,3,3′-tetraethylbenzimidazolocarbo-cyanine iodide (JC-1) were obtained from Beyotime (Shanghai, China). The Mito tracker (deep Red) was supplied by Thermo Fisher (USA). All remaining chemicals employed were of high purity.

### Nanoplastic characterization

Considering the experimental context, we undertook the dilution of the NF PS-NP, GF PS-NP, or PE-NP stock solution utilizing double-distilled water (for the in vivo experiments) or MEM complete medium (for the in vitro experiments). Subsequently, we applied a 5-min ultrasound treatment prior to usage. The size distribution and zeta potential of the PS-NP suspension in various solution systems were assessed through dynamic light scattering using a Zetasizer Nano ZS (Malvern Panalytical GmbH, Kassel, Germany).

### Cell culture

The human SH-SY5Y neuroblastoma cell line was procured from Procell Life Science & Technology Co., Ltd. (Wuhan, China). The cells were cultivated in Minimum Essential Medium/Ham's F12 nutrient medium (Procell), enriched with 10% fetal bovine serum (FBS), penicillin (100 IU/mL), and streptomycin (100 μg/mL), and maintained at 37 °C in a 5% CO_2_ incubator. To induce the dopaminergic neuronal phenotype, an established differentiation protocol was applied to SH-SY5Y cells [83, 84]. Initially, the cells were plated in complete MEM/F12 medium supplemented with 10 μM RA and cultured for a period of 3 days. Subsequently, the complete medium was supplemented with 80 nM PMA, and the cells were nurtured for an additional 3 days. Following a 48-h exposure to PS-NPs, the cells were collected and utilized for subsequent experiments. The PS-NP doses used were 0, 0.5, 5, 50 and 500 μg/mL.

### Cellular uptake of GF PS-NPs

SH-SY5Y cells were plated onto 12-well culture plate at a density of 1 × 10^5^ cells per well and differentiated into dopaminergic neuronal phenotype (*n* = 3 per group). The cells were then exposed to GF PS-NPs at doses of 0, 0.5, 5, 50, and 500 μg/mL for 48 h. Post-treatment, the cells underwent triple washing with phosphate-buffered solution (PBS) for 3 min each. Finally, fluorescence microscope (Nikon Eclipse C1, Tokyo, Japan) was employed to capture images of the cells.

### Cell viability assay

The SH-SY5Y cells were seeded on 96-well culture plates at a density of 1 × 10^4^ cells per well and differentiated into dopaminergic neuronal phenotype. The cells were then exposed to PS-NPs solutions with gradient doses of 0, 0.5, 5, 50 and 500 μg/mL. Post 48 h of incubation, cell viability was assessed using a CCK-8 assay (BestBio, Beijing, China). To ensure minimal interference from PS-NPs, the supernatant was carefully transferred to an empty plate, and the absorbance was quantified at 450 nm utilizing a Spark® multimode microplate reader (TECAN, Männedorf, Switzerland). To validate the impact of PS-NPs on cell viability, PI staining was adopted to identify cells with compromised membrane integrity. SH-SY5Y cells were cultured in 6-well plates and differentiated into dopaminergic neuronal phenotype. Then, the cells were exposed to PS-NPs in a similar manner, and then detached using trypsin without ethylenediaminetetraacetic acid and treated with a 3 μM PI staining solution. Subsequent to a 20-min incubation at 37 °C, flow cytometry analysis was conducted to assess the stained cells. To further investigate the mechanistic aspects of PS-NP-induced cell death, antioxidant NAC (0.5 mM), autophagy inhibitor 3-MA (100 μM), AMPK pathway inhibitor CoC (0.5 μM) or melatonin (16 μM) was separately added for stimulation in the rescue experiment, respectively. PE-NPs were also assessed for cytotoxicity using the aforementioned CCK-8 assay.

### TEM

The cell microstructure and mitochondrial integrity were investigated utilizing TEM. After a 48-h exposure to PS-NPs, the differentiated cells were fixed in 2.5% glutaraldehyde at 4 °C for 30 min. The fixed cells were then washed, treated with osmium tetroxide, and dehydrated using ethanol solutions. Ultrathin cell Sects. (70–90 nm) were obtained using a Leica EM UC735 ultramicrotome and subjected to TEM analysis using a JEM1200EX microscopy (JEOL Ltd., Tokyo, Japan) according a previous protocol [85].

### ROS assay

ROS generation was assessed through a cell-permeable DCFH-DA dye assay. After a 48-h PS-NP exposure, the differentiated cells were treated with 10 μM DCFH-DA at 37 °C for 30 min. After this, the cells underwent three washes with PBS for 3 min each to remove any excess dye, and the fluorescence intensity indicating ROS production was visualized and quantified using a fluorescent microscopy (Nikon Eclipse C1).

### Mitochondrial dysfunction assessment

The assessment of ΔΨm was conducted using a JC-1 assay, following the methodology outlined in our previous research [86, 87]. Cells were treated with a 5 μg/mL JC-1 staining solution (1 mL) for 30 min, followed by PBS rinses. Fluorescent microscopy was used to visualize ΔΨm. Intracellular ATP content was measured in accordance with the manufacturer's instructions.

### Mitochondrial stress test

Mitochondrial respiration assessment was executed using the Seahorse XF extracellular flux analyzer (Agilent, Santa Clara, CA, USA). SH-SY5Y cells were cultured in XF24 cell culture microplates at a density of 8 × 10^3^ cells per well and differentiated into dopaminergic neuronal phenotype. After a 48-h PS-NP exposure, mitochondrial respiration analysis was conducted following the manufacturer's guidelines. In brief, the culture medium was substituted with Agilent Seahorse XF Assay medium containing glucose (10 mM), sodium pyruvate (1 mM), and L-glutamine (2 mM). The Seahorse cartridge was loaded to sequentially dispense specific metabolic compounds at designated intervals: first oligomycin (1.5 μM), followed by FCCP (5 μM), and subsequently rotenone and antimycin A (0.5 μM).

### Molecular docking

Molecular docking was initially employed to analyze the binding interactions between PS-NPs and mitochondrial respiratory complexes. The protein structures corresponding to the complexes (PDB ID: 5XTD, 5XTE, 5Z62, 8GS8, and 8H9S) were acquired from the RSCB Protein Data Bank (https://www.rcsb.org/). Four types of 10-monomer nanoplastics (PS, PP, PE, and PVC) were sourced from the ATB website (https://atb.uq.edu.au), and a 20-monomer PS (n20) nanoplastic was modeled using the same procedure. Molecular docking was conducted using the molecular operating environment (MOE) software [88]. Subsequently, the nanoplastics and respective protein structures were imported into the MOE software, followed by the removal of water molecules and excess ligands. The protonate 3D method assigned implicit hydrogens and ionization states to the processed protein structures. The binding sites were determined using the Site Finder tool, and the initial placement of the nanoplastics was evaluated using the ASE scoring function. Refinement of the initial placements into five poses was conducted using the dG affinity scoring function. Among these candidates, the optimal docking structure with the highest docking score was selected for further docking analysis and MD simulation.

### MD simulations

MD simulations were conducted to dissect the trajectories and interaction energies within the PS-protein complex. The optimal structures obtained from the molecular docking served as the starting conformations for MD simulations. The OPLS-AA force field was utilized to establish topology parameters for both the PS molecules and proteins. The simulation system consisted of a cubic box with initial dimensions of 26 nm, containing a total of 535,402 water molecules. The PS-protein complex was positioned at the center of the water box, and 21 chloride ions were randomly added to ensure net charge neutrality. To achieve a suitable molecular arrangement, an initial energy minimization step was performed to alleviate unfavorable contacts. Subsequently, the system underwent equilibration within the canonical ensemble (NVT) and the isothermal-isobaric ensemble (NPT). After equilibration, a 20 ns MD simulation was conducted with a time step of 2 fs. The temperature was maintained at 310 K and the pressure at 1 bar throughout the simulation. GROMACS v2020.4 was employed for all MD simulations [89]. The gmx_MMPBSA package calculated the binding free energy between the protein and PS molecule [90], and the VMD software was employed for visualization of the simulation results [91].

### Animal experiment design

This investigation was conducted in strict adherence to the guidelines and regulations of the Southern Medical University Scientific Research Committee on Ethics in the Care and Use of Laboratory Animals. The animal experiment protocol was approved and assigned the permit number SMUL2020157. A total of 40 adult male C57BL/6 J mice (18–20 g) were sourced from the Guangdong Medical Laboratory Animal Center (Foshan, China). These mice were housed under controlled conditions featuring a temperature of 23–25 °C, humidity of 50–60%, and a 12-h light/dark cycle (lights on from 8:00 a.m. to 8:00 p.m.). The mice were subjected to random allocation into four groups (*n* = 10/group): control, PS-NPs, melatonin, and PS-NPs + melatonin. Melatonin was dissolved in normal saline at a concentration of 1 mg/mL and stored in a light-shielded environment at 4 °C. All animals in the study underwent two fundamental procedures: intraperitoneal injection and gavage. Specifically, the melatonin group and the PS-NPs + melatonin group received an intraperitoneal injection of melatonin (10 mg/kg/day), while the control group and PS-NPs group received an intraperitoneal injection of physiological saline. After one hour, the PS-NPs group and the PS-NPs + melatonin group received oral gavage of PS-NPs (250 mg/kg/day), while the control group and melatonin group received oral gavage of double distilled water. Following the 28-day treatment duration, all mice were anesthetized using 3% pentobarbital and subsequently perfused with ice-cold PBS. Euthanasia was accomplished via cervical dislocation, and their brains of the mice were collected for further analysis. To delve deeper into the mechanism of PS-NP-induced PD-like neurodegeneration in mice, we dissected the midbrain and striatum regions, which are closely associated with PD, for subsequent analysis.

### Behavioral tests

At the end of the 28-day exposure, we performed three behavioral tests, encompassing the open field test (OFT), rotarod test, and grip strength test, on the animals (*n* = 10 per group), as previously described in our earlier investigations [33]. (1) The open field test, a method employed to evaluate spontaneous activity and locomotion, involved placing each mouse within a square arena (Flyde, Guangzhou, China), enabling free movement for a duration of 5 min. An overhead digital CCD camera meticulously recorded their movements, while their duration in the center of the arena was analyzed through a maze video tracking system (Chucai Electronic Technology Co., Ltd, Shanghai, China). (2) The rotarod test was utilized to assess motor coordination abilities. After a training phase to familiarize the mice with the apparatus, the test commenced, with the speed of the rotarod progressively escalating from 4 to 40 rpm over a 3-min interval. The test data were recorded as the mean latency time (averaged over three trials). (3) The grip strength test measured neuromuscular strength through five attempts to grip a metal grid, with peak holding strength recorded in Newtons (N). All behavioral tests were carried out in the neurobehavioral laboratory between 9:00 and 17:00, under established optimal conditions.

### Nissl staining

The brains were post-fixed in a 4% (w/v) paraformaldehyde solution overnight at 4 °C, followed by embedding in paraffin. Sections of the brain containing the substantia SNc and striata were cut at a thickness of 4 μm. Nissl staining, as per established protocols [33], was conducted. Paraffin sections were then deparaffinized and stained with toluidine blue. The stained sections were meticulously examined and captured using a microscope for the quantifying of Nissl-positive neurons in the striatum and SNc regions. Three images were acquired per target brain region to ensure comprehensive quantification for each brain section.

### IF assay

For differentiated SH-SY5Y cells: After a 48-h PS-NP exposure, cells were treated with Mito track (5 μM) for 30 min, followed by the blocking and incubation overnight at 4 °C with primary LC3 antibody (1:500 dilution). After three washes with PBST, the cells were subjected to incubation with FITC-conjugated goat anti-rabbit IgG and counterstained with DAPI for 1 h at room temperature. The resulting images were captured employing a fluorescence microscope (Carl Zeiss LSM780). For brain tissue: Utilizing the identical paraffin-embedded brain specimens as in the Nissl staining procedure, we proceeded with IF assays co-incubated with anti-tyrosine hydroxylase (TH), anti-voltage-dependent anion channel (VDAC) and anti-LC3 antibodies. The IF experiments followed a previously established protocol [33]. The brain slices were incubated with the primary antibodies, and IF analysis was performed utilizing a fluorescence microscope (Carl Zeiss LSM780). Additional information about the antibodies used can be found in Additional file 1: Table S1.

### Protein extraction and western blotting

3-MA (100 μM), CoC (0.5 μM) and melatonin (16 μM) were applied in SH-SY5Y cells for different stimulations. The collected cells and mouse brain tissues were used for protein extraction and separated by electrophoresis, following established protocols [92]. In brief, the harvested cell samples were incubated with RIPA buffer (Beyotime, Shanghai, China), supplemented with a 1:100 dilution of phosphatase and protease inhibitor cocktail (Keygen, Nanjing, China). For tissue protein extraction, the midbrain and striatum were lysed using RIPA buffer supplemented with a 1:100 dilution of phosphatase and protease inhibitor cocktail. The tissue lysates were further homogenized using a High-speed low-temperature tissue homogenizer (Servicebio, Wuhan, China) at 60 Hz for 3 min to ensure complete disruption of tissue structures and efficient protein extraction. The total proteins were separated by electrophoresis on 8 to 15% SDS–polyacrylamide gels and subsequently transferred to 4.5 µm polyvinylidene fluoride membranes. After a blocking step involving 5% nonfat milk, the membranes underwent an overnight incubation at 4 °C with primary antibodies, followed by a subsequent 1-h incubation with the secondary antibody at 37 °C. ECL (Millipore, Billerica, MA, USA) and a Tanon-5200 chemical luminescence developing system (Tanon, Shanghai, China) were employed to visualize protein bands (Additional file 2). ImageJ software was used to analyze the optical densities. Comprehensive details regarding the antibodies used are available in the Additional file 1: Table S2.

### Statistical analysis

The presented data are expressed as mean ± standard deviation (SD), unless stated otherwise. Statistical analysis was carried out using SPSS 22.0 (IBM, Armonk, NY, USA) and Prism 8.0 (GraphPad Software, Inc., San Diego, CA, USA). To assess the statistical significance of distinctions between two groups, a Student's *t*-test was applied. For comparisons involving multiple exposure groups and their corresponding controls in each exposure experiment, a one-way analysis of variance (ANOVA) was employed, followed by Tukey's multiple comparisons test. A *P*-value below 0.05 was deemed statistically significant (Additional file 2).

### Supplementary Information

**Additional file 1.** Supplementary table and figures.

**Additional file 2.** Original bands of Western blots used in this study.

## Data Availability

The datasets during and/or analyzed during the current study are available from the corresponding author on reasonable request.

## Abbreviations

3-MA: 3-Methyladenine
AMPK: AMP-activated protein kinase
ANOVA: Analysis of variance
BBB: Blood–brain barrier
CCK-8: Cell counting kit-8
CI: Complex I
CoC: Compound C
DCFH-DA: 2′,7′-Dichlorodihydrofluorescein diacetate
ETC: Electron transport chain
GF: Green fluorescent
IF: Immunofluorescence
JC-1: 5,5′,6,6′-Tetrachloro-1,1′,3,3′-tetraethylbenzimidazolocarbo-cyanine iodide
LC3: Microtubule-associated protein 1 light chain
MD: Molecular dynamics
MNP: Microplastic and nanoplastic
MOE: Molecular operating environment
mPTP: Mitochondrial permeability transition pore
NAC: N-acetyl-Lcysteine
NADH: Nicotinamide adenine dinucleotide
NF: Non-fluorescent
OFT: Open field test
OXPHOS: Oxidative phosphorylation
PBS: Phosphate-buffered solution
PD: Parkinson's disease
PDI: Polymer dispersion index
PE: Polyethylene
PE-NP: Polyethylene nanoplastic
PI: Propidium iodide
PMA: Phorbol 12-myristate 13-acetate
PP: Polypropylene
PS-NP: Polystyrene nanoplastics
PVC: Polyvinyl chloride
Q10: Ubiquinone-10
RA: Retinoic acid
SD: Standard deviation
SEM: Scanning electron microscope
SNc: Substantia nigra pars compacta
TEM: Transmission electron microscopy
TH: Tyrosine hydroxylase
VDAC: Voltage-dependent anion channe
ΔΨm: Mitochondrial membrane potential
ΔEvdw: Van der Waals energy

## References

[1] Amato-Lourenco LF, Galvao LD, Wiebeck H, Carvalho-Oliveira R, Mauad T (2022). Atmospheric microplastic fallout in outdoor and indoor environments in Sao Paulo megacity. Sci Total Environ.

[2] Chen H, Chen YH, Xu YB, Xiao CQ, Liu JC, Wu RR (2022). Different functional areas and human activities significantly affect the occurrence and characteristics of microplastics in soils of the Xi'an metropolitan area. Sci Total Environ.

[3] Gao SK, Yan K, Liang BG, Shu RL, Wang N, Zhang S (2023). The different ways microplastics from the water column and sediment accumulate in fish in Haizhou Bay. Sci Total Environ.

[4] Yuan WK, Christie-Oleza JA, Xu EG, Li JW, Zhang HB, Wang WF (2022). Environmental fate of microplastics in the world's third-largest river: basin-wide investigation and microplastic community analysis. Water Res.

[5] Wen SY, Zhao Y, Wang MQ, Yuan HB, Xu HY. Micro(nano)plastics in food system: potential health impacts on human intestinal system. Crit Rev Food Sci. 2022:1–19. 10.1080/10408398.2022.2116559 36066327

[6] Chaudhari S, Samnani P (2023). Determination of microplastics in pond water. Mater Today Proc..

[7] Wu PF, Lin SY, Cao GD, Wu JB, Jin HB, Wang C (2022). Absorption, distribution, metabolism, excretion and toxicity of microplastics in the human body and health implications. J Hazard Mater.

[8] Gall SC, Thompson RC (2015). The impact of debris on marine life. Mar Pollut Bull.

[9] Schwabl P, Köppel S, Königshofer P, Bucsics T, Trauner M, Reiberger T (2019). Detection of various microplastics in human stool: a prospective case series. Ann Intern Med.

[10] Amato-Lourenco LF, Carvalho-Oliveira R, Junior GR, Dos Santos GL, Ando RA, Mauad T (2021). Presence of airborne microplastics in human lung tissue. J Hazard Mater.

[11] Leslie HA, van Velzen MJM, Brandsma SH, Vethaak AD, Garcia-Vallejo JJ, Lamoree MH (2022). Discovery and quantification of plastic particle pollution in human blood. Environ Int.

[12] Wu D, Feng Y, Wang R, Jiang J, Guan Q, Yang X (2023). Pigment microparticles and microplastics found in human thrombi based on Raman spectral evidence. J Adv Res.

[13] Ragusa A, Svelato A, Santacroce C, Catalano P, Notarstefano V, Carnevali O (2021). Plasticenta: first evidence of microplastics in human placenta. Environ Int.

[14] Zhao Q, Zhu L, Weng J, Jin Z, Cao Y, Jiang H (2023). Detection and characterization of microplastics in the human testis and semen. Sci Total Environ.

[15] Liang B, Zhong Y, Huang Y, Lin X, Liu J, Lin L (2021). Underestimated health risks: polystyrene micro- and nanoplastics jointly induce intestinal barrier dysfunction by ROS-mediated epithelial cell apoptosis. Part Fibre Toxicol.

[16] Huang ZZ, Weng Y, Shen QC, Zhao Y, Jin YX (2021). Microplastic: A potential threat to human and animal health by interfering with the intestinal barrier function and changing the intestinal microenvironment. Sci Total Environ.

[17] Li Y, Shi T, Li X, Sun H, Xia X, Ji X (2022). Inhaled tire-wear microplastic particles induced pulmonary fibrotic injury via epithelial cytoskeleton rearrangement. Environ Int.

[18] Li S, Shi M, Wang Y, Xiao Y, Cai D, Xiao F (2021). Keap1-Nrf2 pathway up-regulation via hydrogen sulfide mitigates polystyrene microplastics induced-hepatotoxic effects. J Hazard Mater.

[19] Wang Y-L, Lee Y-H, Hsu Y-H, Chiu IJ, Huang CC-Y, Huang C-C, et al. The kidney-related effects of polystyrene microplastics on human kidney proximal tubular epithelial cells HK-2 and male C57BL/6 mice. Environ Health Perspect. 2021;129(5):57003. 10.1289/EHP7612 PMC8101928 33956507

[20] Zhu X, Wang C, Duan X, Liang B, Genbo XuE, Huang Z (2023). Micro- and nanoplastics: a new cardiovascular risk factor?. Environ Int.

[21] Li ZK, Zhu SX, Liu Q, Wei JL, Jin YC, Wang XF (2020). Polystyrene microplastics cause cardiac fibrosis by activating Wnt/β-catenin signaling pathway and promoting cardiomyocyte apoptosis in rats. Environ Pollut.

[22] Prüst M, Meijer J, Westerink RHS. The plastic brain: neurotoxicity of micro- and nanoplastics. Part Fibre Toxicol. 2020;17(1). 10.1186/s12989-020-00358-y PMC7282048 32513186

[23] Yang D, Zhu J, Zhou X, Pan D, Nan S, Yin R (2022). Polystyrene micro- and nano-particle coexposure injures fetal thalamus by inducing ROS-mediated cell apoptosis. Environ Int.

[24] Kopatz V, Wen KV, Kovacs T, Keimowitz AS, Pichler V, Widder J, et al. Micro- and nanoplastics breach the blood-brain barrier (BBB): biomolecular corona's role revealed. Nanomaterials. 2023;13(8). 10.3390/nano13081404 PMC10141840 37110989

[25] Zeng W, Yang F, Shen WL, Zhan C, Zheng P, Hu J (2022). Interactions between central nervous system and peripheral metabolic organs. Sci China Life Sci.

[26] Zhao Y-L, Qu Y, Ou Y-N, Zhang Y-R, Tan L, Yu J-T (2021). Environmental factors and risks of cognitive impairment and dementia: a systematic review and meta-analysis. Ageing Res Rev.

[27] Khan A, Plana-Ripoll O, Antonsen S, Brandt J, Geels C, Landecker H (2019). Environmental pollution is associated with increased risk of psychiatric disorders in the US and Denmark. PLoS Biol.

[28] Ascherio A, Schwarzschild MA (2016). The epidemiology of Parkinson's disease: risk factors and prevention. Lancet Neurol.

[29] Calderón-Garcidueñas L, Ayala A (2022). Air pollution, ultrafine particles, and your brain: are combustion nanoparticle emissions and engineered nanoparticles causing preventable fatal neurodegenerative diseases and common neuropsychiatric outcomes?. Environ Sci Technol.

[30] Kik K, Bukowska B, Sicinska P. Polystyrene nanoparticles: Sources, occurrence in the environment, distribution in tissues, accumulation and toxicity to various organisms. Environmental Pollution. 2020;262. 10.1016/j.envpol.2020.114297 32155552

[31] Liu XY, Zhao YC, Dou JB, Hou QH, Cheng JX, Jiang XY (2022). Bioeffects of inhaled nanoplastics on neurons and alteration of animal behaviors through deposition in the brain. Nano Lett.

[32] Shan S, Zhang YF, Zhao HW, Zeng T, Zhao XL (2022). Polystyrene nanoplastics penetrate across the blood-brain barrier and induce activation of microglia in the brain of mice. Chemosphere.

[33] Liang B, Huang Y, Zhong Y, Li Z, Ye R, Wang B (2022). Brain single-nucleus transcriptomics highlights that polystyrene nanoplastics potentially induce Parkinson's disease-like neurodegeneration by causing energy metabolism disorders in mice. J Hazard Mater.

[34] Nunnari J, Suomalainen A (2012). Mitochondria: in sickness and in health. Cell.

[35] Subramaniam SR, Chesselet M-F (2013). Mitochondrial dysfunction and oxidative stress in Parkinson's disease. Prog Neurobiol.

[36] Lou G, Palikaras K, Lautrup S, Scheibye-Knudsen M, Tavernarakis N, Fang EF (2020). Mitophagy and neuroprotection. Trends Mol Med.

[37] Yan T, Zhao Y, Jiang Z, Chen J (2022). Acetaldehyde induces cytotoxicity via triggering mitochondrial dysfunction and overactive mitophagy. Mol Neurobiol.

[38] Li J, Lai M, Zhang X, Li Z, Yang D, Zhao M (2022). PINK1-parkin-mediated neuronal mitophagy deficiency in prion disease. Cell Death Dis.

[39] Chitimus DM, Popescu MR, Voiculescu SE, Panaitescu AM, Pavel B, Zagrean L, et al. Melatonin's impact on antioxidative and anti-Inflammatory reprogramming in homeostasis and disease. Biomolecules. 2020;10(9). 10.3390/biom10091211 PMC7563541 32825327

[40] Tan HY, Ng KY, Koh RY, Chye SM (2020). pharmacological effects of melatonin as neuroprotectant in rodent model: a review on the current biological evidence. Cell Mol Neurobiol.

[41] Chen C, Yang C, Wang J, Huang X, Yu H, Li S (2021). Melatonin ameliorates cognitive deficits through improving mitophagy in a mouse model of Alzheimer's disease. J Pineal Res.

[42] Jiménez-Delgado A, Ortiz GG, Delgado-Lara DL, González-Usigli HA, González-Ortiz LJ, Cid-Hernández M, et al. Effect of melatonin administration on mitochondrial activity and oxidative stress markers in patients with Parkinson's disease. Oxid Med Cell Longev. 2021:5577541. 10.1155/2021/5577541 PMC8545577 34707777

[43] Liu Z, Hua X, Zhao Y, Bian Q, Wang D. Polyethylene nanoplastics cause reproductive toxicity associated with activation of both estrogenic hormone receptor NHR-14 and DNA damage checkpoints in C. elegans. Sci Total Environ. 2023;906:167471. 10.1016/j.scitotenv.2023.167471 37778542

[44] Gu JK, Liu TY, Guo RY, Zhang LX, Yang MJ (2022). The coupling mechanism of mammalian mitochondrial complex I. Nat Struct Mol Biol.

[45] Beaupre LMM, Brown GM, Goncalves VF, Kennedy JL (2021). Melatonin's neuroprotective role in mitochondria and its potential as a biomarker in aging, cognition and psychiatric disorders. Transl Psychiat.

[46] Pfanner N, Warscheid B, Wiedemann N (2019). Mitochondrial proteins: from biogenesis to functional networks. Nat Rev Mol Cell Biol.

[47] Lin SY, Zhang HN, Wang C, Su XL, Song YY, Wu PF (2022). Metabolomics reveal nanoplastic-induced mitochondrial damage in human liver and lung cells. Environ Sci Technol.

[48] Liu L, Liu BY, Zhang BW, Ye YY, Jiang W (2022). Polystyrene micro(nano)plastics damage the organelles of RBL-2H3 cells and promote MOAP-1 to induce apoptosis. J Hazard Mater.

[49] Li YJ, Guo MH, Niu SY, Shang MT, Chang XR, Sun ZY (2023). ROS and DRP1 interactions accelerate the mitochondrial injury induced by polystyrene nanoplastics in human liver HepG2 cells. Chem-Biol Interact.

[50] Lee SE, Yi Y, Moon S, Yoon H, Park YS (2022). Impact of micro- and nanoplastics on mitochondria. Metabolites.

[51] Trevisan R, Voy C, Chen SX, Di Giulio RT (2019). Nanoplastics decrease the toxicity of a complex PAH mixture but impair mitochondrial energy production in developing zebrafish. Environ Sci Technol.

[52] Wang SY, Liu MH, Wang JM, Huang JS, Wang J (2020). Polystyrene nanoplastics cause growth inhibition, morphological damage and physiological disturbance in the marine microalga Platymonas helgolandica. Mar Pollut Bull.

[53] Parey K, Wirth C, Vonck J, Zickermann V (2020). Respiratory complex I - structure, mechanism and evolution. Curr Opin Struct Biol.

[54] Sazanov LA (2015). A giant molecular proton pump: structure and mechanism of respiratory complex I. Nat Rev Mol Cell Biol.

[55] Basit F, van Oppen LMPE, Schockel L, Bossenbroek HM, van Emst-de Vries SE, Hermeling JCW (2017). Mitochondrial complex I inhibition triggers a mitophagy-dependent ROS increase leading to necroptosis and ferroptosis in melanoma cells. Cell Death Dis.

[56] Marella M, Seo BB, Nakamaru-Ogiso E, Greenamyre JT, Matsuno-Yagi A, Yagi T (2008). Protection by the NDI1 gene against neurodegeneration in a rotenone rat model of Parkinson's disease. PLoS ONE.

[57] Rajendran D, Chandrasekaran N, Waychal Y, Mukherjee A (2022). Nanoplastics alter the conformation and activity of human serum albumin. Nanoimpact.

[58] Li WW, Pan Z, Xu J, Liu QL, Zou QP, Lin H (2022). Microplastics in a pelagic dolphinfish (Coryphaena hippurus) fromthe Eastern Pacific Ocean and the implications for fish health. Sci Total Environ.

[59] Teng MM, Zhao XL, Wu FC, Wang CJ, Wang C, White JC (2022). Charge-specific adverse effects of polystyrene nanoplastics on zebrafish (Danio rerio) development and behavior. Environ Int.

[60] Wei W, Li YH, Lee M, Andrikopoulos N, Lin SJ, Chen CY, et al. Anionic nanoplastic exposure induces endothelial leakiness. Nature Communications. 2022;13(1). 10.1038/s41467-022-32532-5 PMC9376074 35963861

[61] Choi GE, Lee HJ, Chae CW, Cho JH, Jung YH, Kim JS (2021). BNIP3L/NIX-mediated mitophagy protects against glucocorticoid-induced synapse defects. Nat Commun.

[62] Wang Y, Tang CY, Cai J, Chen GC, Zhang DS, Zhang ZH (2018). PINK1/Parkin-mediated mitophagy is activated in cisplatin nephrotoxicity to protect against kidney injury. Cell Death Dis.

[63] Guo X, Sun XY, Hu D, Wang YJ, Fujioka H, Vyas R (2016). VCP recruitment to mitochondria causes mitophagy impairment and neurodegeneration in models of Huntington's disease. Nat Commun.

[64] Jin QH, Li RB, Hu N, Xin T, Zhu PJ, Hu SY (2018). DUSP1 alleviates cardiac ischemia/reperfusion injury by suppressing the Mff-required mitochondrial fission and Bnip3-related mitophagy via the JNK pathways. Redox Biol.

[65] Herzig S, Shaw RJ (2018). AMPK: guardian of metabolism and mitochondrial homeostasis. Nat Rev Mol Cell Biol.

[66] Liang JY, Xu ZX, Ding ZY, Lu YL, Yu QH, Werle KD (2015). Myristoylation confers noncanonical AMPK functions in autophagy selectivity and mitochondrial surveillance. Nat Commun.

[67] Tian WL, Li W, Chen YQ, Yan ZM, Huang X, Zhuang HX (2015). Phosphorylation of ULK1 by AMPK regulates translocation of ULK1 to mitochondria and mitophagy. Febs Lett.

[68] Zhang Q, Xu EG, Li JN, Chen QQ, Ma LP, Zeng EY (2020). A review of microplastics in table salt, drinking water, and air:direct human exposure. Environ Sci Technol.

[69] Pei HF, Hou JN, Wei FP, Xue Q, Zhang F, Peng CF (2017). Melatonin attenuates postmyocardial infarction injury via increasing Tom70 expression. J Pineal Res.

[70] Martin M, Macias M, Escames G, Reiter RJ, Agapito MT, Ortiz GG (2000). Melatonin-induced increased activity of the respiratory chain complexes I and IV can prevent mitochondrial damage induced by ruthenium red in vivo. J Pineal Res.

[71] Martin M, Macias M, Leon J, Escames G, Khaldy H, Acuna-Castroviejo D (2002). Melatonin increases the activity of the oxidative phosphorylation enzymes and the production of ATP in rat brain and liver mitochondria. Int J Biochem Cell Biol.

[72] Ding S, Lin N, Sheng XC, Zhao YC, Su YY, Xu LW (2019). Melatonin stabilizes rupture-prone vulnerable plaques via regulating macrophage polarization in a nuclear circadian receptor ROR alpha-dependent manner. J Pineal Res.

[73] Zhang HM, Zhang YQ (2014). Melatonin: a well-documented antioxidant with conditional pro-oxidant actions. J Pineal Res.

[74] Dong LX, Sun Q, Qiu HX, Yang KC, Xiao BY, Xia T (2023). Melatonin protects against developmental PBDE-47 neurotoxicity by targeting the AMPK/mitophagy axis. J Pineal Res.

[75] Jiang Y, Shen M, Chen YY, Wei YH, Tao JL, Liu HL (2021). Melatonin represses mitophagy to protect mouse granulosa cells from oxidative damage. Biomolecules.

[76] Niu YJ, Zhou WJ, Nie ZW, Shin KT, Cui XS (2020). Melatonin enhances mitochondrial biogenesis and protects against rotenone-induced mitochondrial deficiency in early porcine embryos. J Pineal Res.

[77] Kato H, Tanaka G, Masuda S, Ogasawara J, Sakurai T, Kizaki T (2015). Melatonin promotes adipogenesis and mitochondrial biogenesis in 3T3-L1 preadipocytes. J Pineal Res.

[78] Tan DX, Manchester LC, Qin LL, Reiter RJ (2016). Melatonin: a mitochondrial targeting molecule involving mitochondrial protection and dynamics. Int J Mol Sci.

[79] Wang B, Liang B, Huang Y, Li Z, Zhang B, Du J (2023). Long-chain acyl carnitines aggravate polystyrene nanoplastics-induced atherosclerosis by upregulating MARCO. Adv Sci (Weinh).

[80] Mattsson K, Hansson LA, Cedervall T (2015). Nano-plastics in the aquatic environment. Environ Sci Process Impacts.

[81] Lambert S, Wagner M (2016). Characterisation of nanoplastics during the degradation of polystyrene. Chemosphere.

[82] Sharma VK, Ma XM, Lichtfouse E, Robert D (2023). Nanoplastics are potentially more dangerous than microplastics. Environ Chem Lett.

[83] Presgraves SP, Ahmed T, Borwege S, Joyce JN (2004). Terminally differentiated SH-SY5Y cells provide a model system for studying neuroprotective effects of dopamine agonists. Neurotox Res.

[84] Ferreira PS, Nogueira TB, Costa VM, Branco PS, Ferreira LM, Fernandes E (2013). Neurotoxicity of "ecstasy" and its metabolites in human dopaminergic differentiated SH-SY5Y cells. Toxicol Lett.

[85] Liu ZX, Lv XY, Xu L, Liu XT, Zhu XY, Song EQ (2020). Zinc oxide nanoparticles effectively regulate autophagic cell death by activating autophagosome formation and interfering with their maturation. Part Fibre Toxicol.

[86] Hu MJ, Zhong YZ, Liu J, Zheng SZ, Lin L, Lin X (2022). An adverse outcome pathway-based approach to assess aurantio-obtusin-induced hepatotoxicity. Toxicology.

[87] Liang BX, Zhong YZ, Wang B, Lin L, Liu J, Lin X (2021). 1,2-Dichloroethane induces apoptosis in the cerebral cortexes of NIH Swiss mice through microRNA-182-5p targeting phospholipase D1 via a mitochondria-dependent pathway. Toxicol Appl Pharm.

[88] Vilar S, Cozza G, Moro S (2008). Medicinal chemistry and the molecular operating environment (MOE): application of QSAR and molecular docking to drug discovery. Curr Top Med Chem.

[89] Van der Spoel D, Lindahl E, Hess B, Groenhof G, Mark AE, Berendsen HJC (2005). GROMACS: fast, flexible, and free. J Comput Chem.

[90] Valdes-Tresanco MS, Valdes-Tresanco ME, Valiente PA, Moreno E (2021). Gmx_MMPBSA: a new tool to perform end-state free energy calculations with GROMACS. J Chem Theory Comput.

[91] Humphrey W, Dalke A, Schulten K (1996). VMD: visual molecular dynamics. J Mol Graph..

[92] Zhong YZ, Liang BX, Meng H, Ye RY, Li ZM, Du JX (2022). 1,2-Dichloroethane induces cortex demyelination by depressing myelin basic protein via inhibiting aquaporin 4 in mice. Ecotox Environ Safe.

