# Supplementary material for: Polystyrene nanoplastic exposure induces excessive mitophagy by activating AMPK/ULK1 pathway in differentiated SH-SY5Y cells and dopaminergic neurons in vivo
Source: Part Fibre Toxicol. 2023 Nov 22;20:44. doi: 10.1186/s12989-023-00556-4 (PMC10664492; doi:10.1186/s12989-023-00556-4)
Supplement: Supplementary file 1 — Additional file 1. Supplementary table and figures. [file 12989_2023_556_MOESM1_ESM.docx]

**Supplementary Material for**

**Polystyrene nanoplastic exposure induces excessive mitophagy by activating AMPK/ULK1 pathway in differentiated SH-SY5Y cells and dopaminergic neurons *in vivo***

Yuji Huang ^1^, Boxuan Liang ^1^, Zhiming Li ^1^, Yizhou Zhong ^1^, Bo Wang ^1^, Bingli Zhang ^1^, Jiaxin Du ^1^, Rongyi Ye ^1^, Hongyi Xian ^1^, Weicui Min ^2^, Xiliang Yan ^3^, Yanhong Deng ^1^, Yu Feng ^1^, Ruobing Bai ^1^, Bingchi Fan ^1^, Xingfen Yang ^4^, Zhenlie Huang ^1,^*

^1^ *NMPA Key Laboratory for Safety Evaluation of Cosmetics*, *Guangdong Provincial Key Laboratory of Tropical Disease Research, Department of Toxicology, School of Public Health, Southern Medical University, Guangzhou 510515, China;*

^2^ *School of Environmental Science and Engineering, Shandong University, Qingdao 266237, China;*

^3^ *Institute of Environmental Research at Greater Bay Area, Key Laboratory for Water Quality and Conservation of the Pearl River Delta, Ministry of Education, Guangzhou University, Guangzhou 510006, China;*

^4^ *NMPA Key Laboratory for Safety Evaluation of Cosmetics*, *Food Safety and Health Research Center, School of Public Health, Southern Medical University, Guangzhou 510515, PR China;*

***Corresponding author**

huangzhenlie858252@smu.edu.cn (Zhenlie Huang)

**Summary (16 Pages, 2 Tables, 12 Figures)**

Table S1. Information about the antibodies used in immunofluorescence assay**3**

Table S2. Information about the antibodies used in western blotting **4**

Fig. S1. TEM images of differentiated SH-SY5Y cells exposure to PS-NPs **5**

Fig. S2. The effect of NAC on ROS levels in cells exposed to PS-NPs **6**

Fig. S3. Representative western blot gel images in Figure 2 **7**

Fig. S4. Molecular docking analyzing the binding interactions between PS-NPs and mitochondrial respiratory complexes **8**

Fig. S5. Molecular docking analyzing the binding interactions between complex I and different nanoplastics.**9**

Fig. S6. The influence of PE-NPs on cell viability and mitochondrial function in differentiated SH-SY5Y cells **10**

Fig. S7. Representative western blot gel images in Figure 4 **11**

Fig. S8. Representative western blot gel images in Figure 5 **12**

Fig. S9. The effect of melatonin on ROS levels in cells exposed to PS-NPs **13**

Fig. S10. Representative western blot gel images in Figure 6 **14**

Fig. S11. Representative western blot gel images in Figure 7 **15**

Fig. S12. Melatonin mitigating PS-NP-induced mitochondrial loss in dopaminergic neurons in the striatum **16**

Table S1. Information about the antibodies used in immunofluorescence assay

| Primary  antibody | Manufacturer | Catalog | Dilution | Second  antibody | Manufacturer | Catalog | Dilution |
| --- | --- | --- | --- | --- | --- | --- | --- |
| LC3 | Proteintech | 14600-1-AP | 1:1000 | Anti-rabbit | Servicebio | GB23303 | 1:500 |
| TH | Servicebio | GB12181-100 | 1:5000 | Anti- rabbit | Servicebio | GB23303 | 1:500 |
| VDAC | CST | 4661 | 1:1000 | Anti- rabbit | Servicebio | GB23303 | 1:500 |

Table S2. Information about the antibodies used in western blotting

| Primary  antibody | Manufacturer | Catalog | Dilution | Second  antibody | Manufacturer | Catalog | Dilution |
| --- | --- | --- | --- | --- | --- | --- | --- |
| LC3 | Proteintech | 14600-1-AP | 1:1000 | Anti-rabbit | CST | #7074S | 1:5000 |
| AMPK | Proteintech | 10929-2-AP | 1:1000 | Anti- rabbit | CST | #7074S | 1:5000 |
| pULK1 | Proteintech | 80218-1-RR | 1:1000 | Anti-rabbit | CST | #7074S | 1:5000 |
| PINK1 | Proteintech | 23274-1-AP | 1:1000 | Anti- rabbit | CST | #7074S | 1:5000 |
| UQCRC2 | Proteintech | 14742-1-AP | 1:1000 | Anti-rabbit | CST | #7074S | 1:5000 |
| MTCO2 | Proteintech | 55070-1-AP | 1:1000 | Anti- rabbit | CST | #7074S | 1:5000 |
| SDHB | Proteintech | 10620-1-AP | 1:1000 | Anti- rabbit | CST | #7074S | 1:5000 |
| p-AMPK | Immunoway | YP0575 | 1:1000 | Anti-rabbit | CST | #7074S | 1:5000 |
| Parkin | Immunoway | YT3591 | 1:1000 | Anti- rabbit | CST | #7074S | 1:5000 |
| ULK1 | Immunoway | YT4819 | 1:1000 | Anti-rabbit | CST | #7074S | 1:5000 |
| ATP5A | Immunoway | YT0399 | 1:1000 | Anti- rabbit | CST | #7074S | 1:5000 |
| NDUFB8 | Immunoway | YN0930 | 1:1000 | Anti- rabbit | CST | #7074S | 1:5000 |
| p62 | Servicebio | GB11531-100 | 1:1000 | Anti- rabbit | CST | #7074S | 1:5000 |
| ACTB | Sigma | A1978 | 1:5000 | Anti-mouse | CST | #7076S | 1:5000 |

**
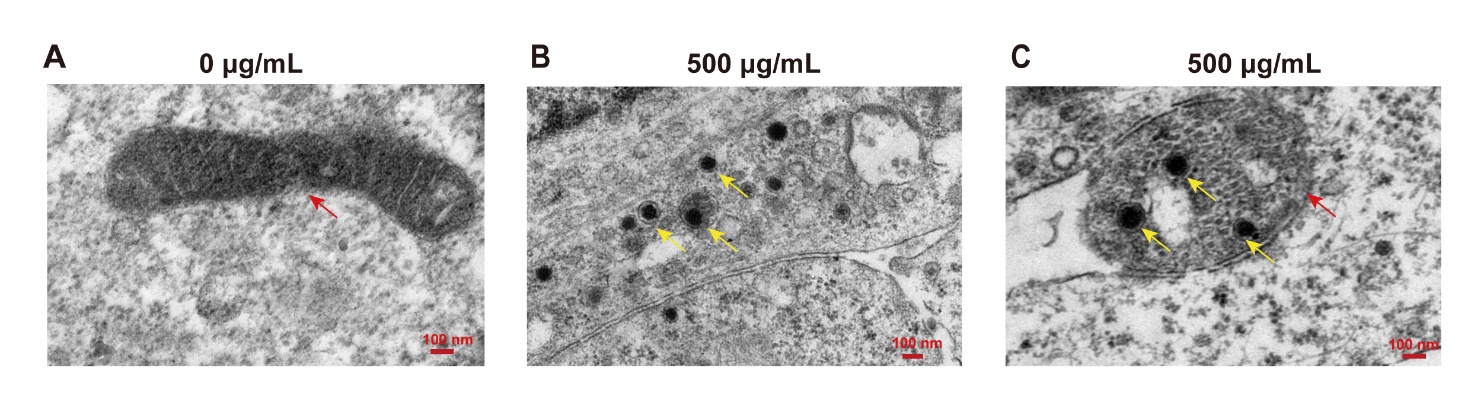
**

**Fig. S1.** TEM images of differentiated SH-SY5Y cells exposure to PS-NPs. Representative TEM images of differentiated SH-SY5Y cells exposure to PS-NPs in (A) 0 μg/mL and (B, C) 500 μg/mL. Red arrows indicate mitochondria, and yellow arrows indicate PS-NPs in mitochondria.


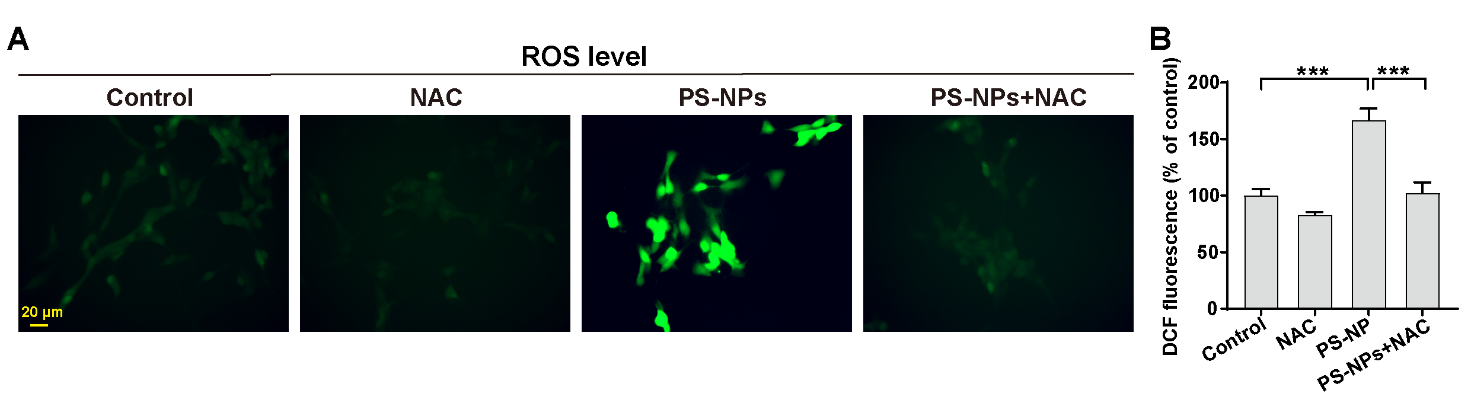


**Fig. S2.** The effect of NAC on ROS levels in cells exposed to PS-NPs. Differentiated SH-SY5Y cells were treated with 500 μg/mL PS-NPs in the presence or absence of 0.5 mM NAC for 48 h and stained with 10 μM DCFH-DA. (A) Representative images. (B) Quantification of ROS levels. *** *P* < 0.001.


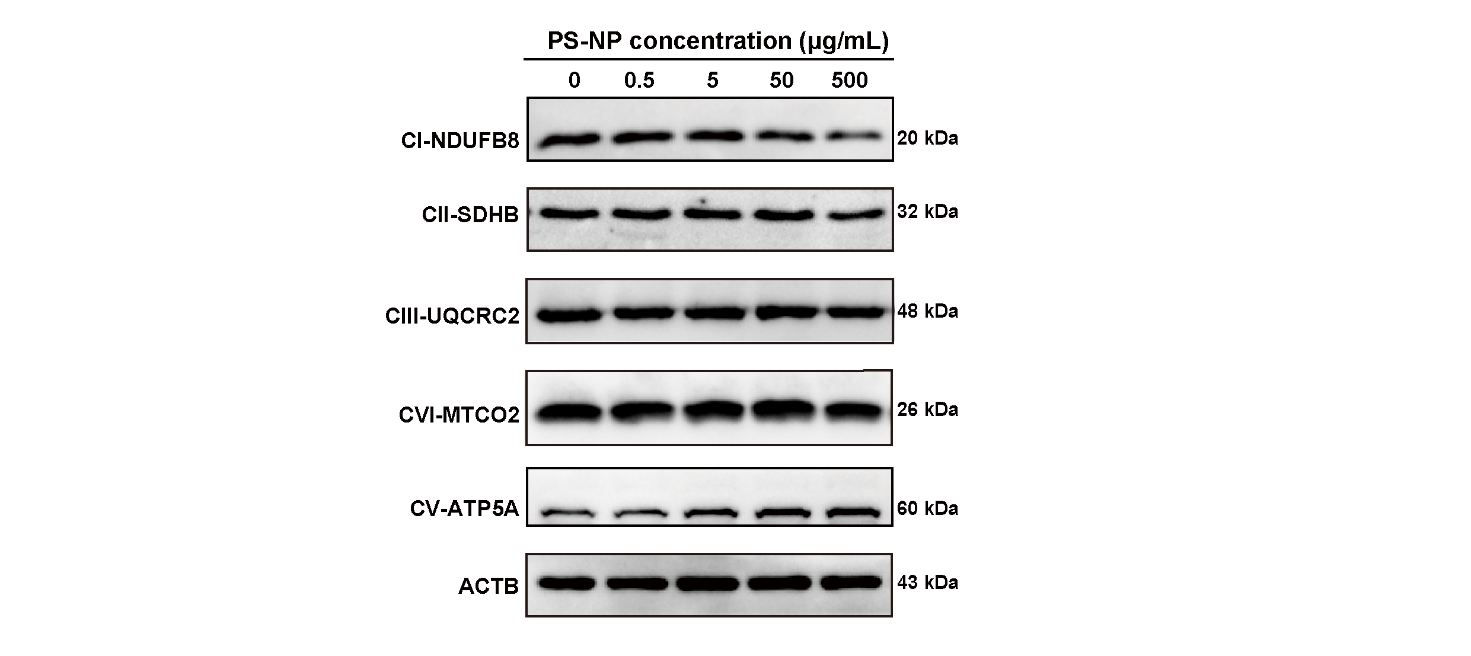


**Fig. S3.** Representative western blot gel images in Figure 2. Western blot gels of CI-NDUFB8, CII-SDHB, CIII-UQCRC2, CVI-MTCO2 and CV-ATP5A in differentiated SH-SY5Y cells treated with PS-NPs.


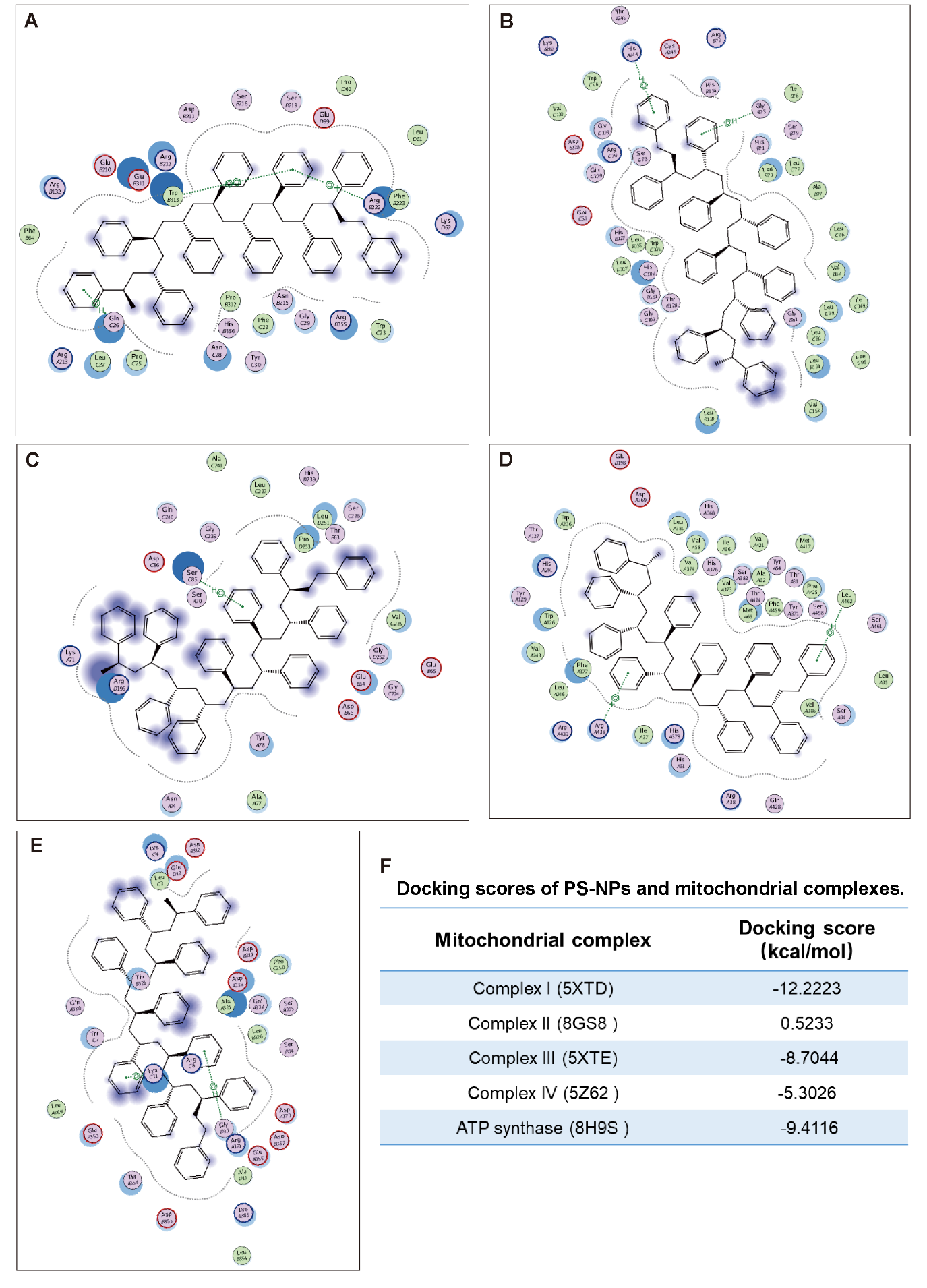


**Fig. S4.** Molecular docking analyzing the binding interactions between PS-NPs and mitochondrial respiratory complexes. Molecular docking model of PS-NPs and mitochondrial complex: (A) Complex I, (B) Complex II, (C) Complex III, (D) Complex IV, (E) ATP synthase. (F) Docking scores of PS-NPs and different mitochondrial complexes.


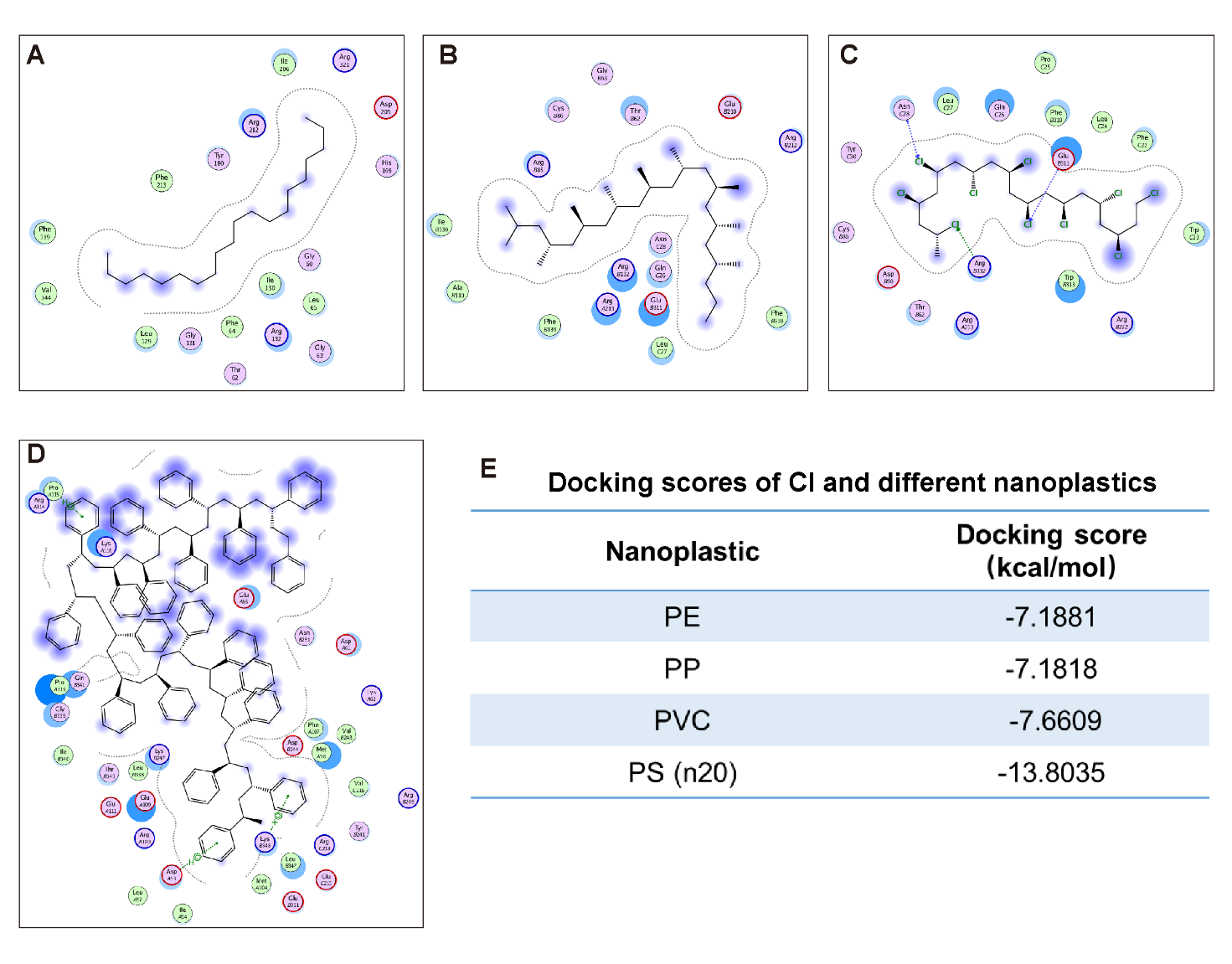


**Fig. S5.** Molecular docking analyzing the binding interactions between complex I and different nanoplastics. Molecular docking model of complex I and nanoplastics: (A) PE, (B) PP, (C) PVC, (D) PS (n20). (E) Docking scores of complex I and different nanoplastics.


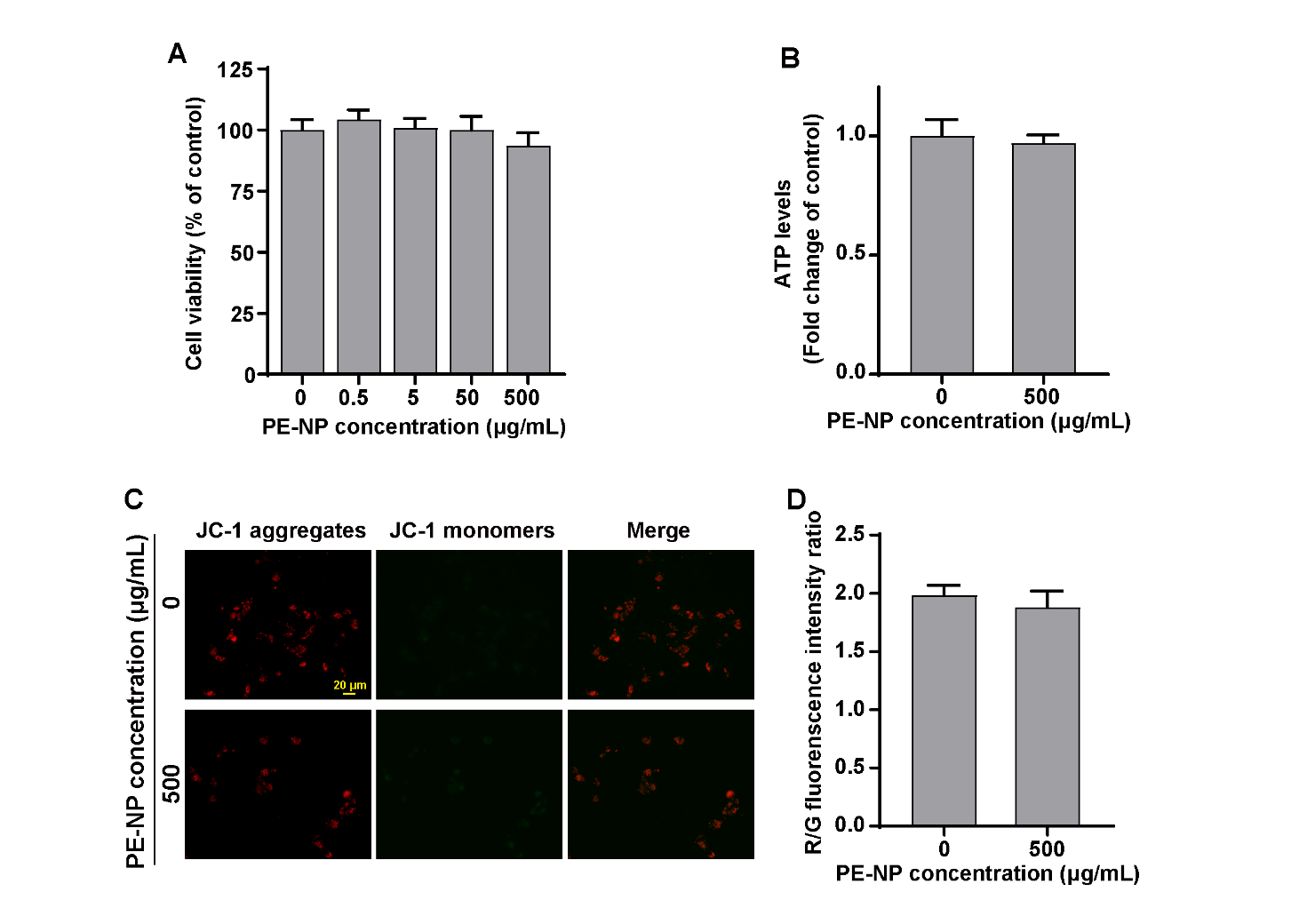


**Fig. S6.** The influence of PE-NPs on cell viability and mitochondrial function in differentiated SH-SY5Y cells. (A) Cell viability after 48 hours of exposure to PE-NPs. (B) Cellular ATP levels. (C) Representative images of the mitochondrial membrane potential (ΔΨm). (D) Quantification of the ΔΨm.


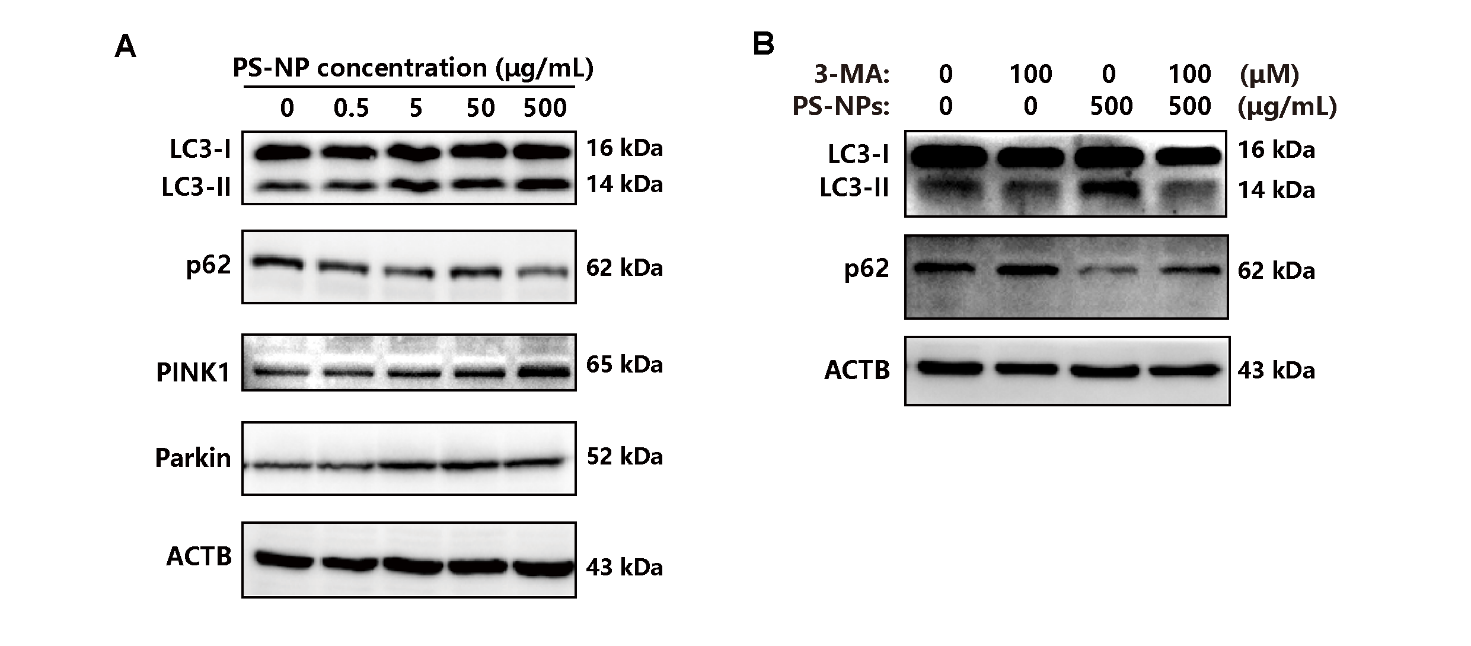


**Fig. S7.** Representative western blot gel images in Figure 4. (A) Western blot gels of LC3-II/LC3-I, p62, PINK1 and Parkin in differentiated SH-SY5Y cells treated with PS-NPs. (B) Western blot gels of LC3-II/LC3-I and p62 in differentiated SH-SY5Y cells exposed to 500 μg/mL PS-NPs with or without 3-MA (100 μM).


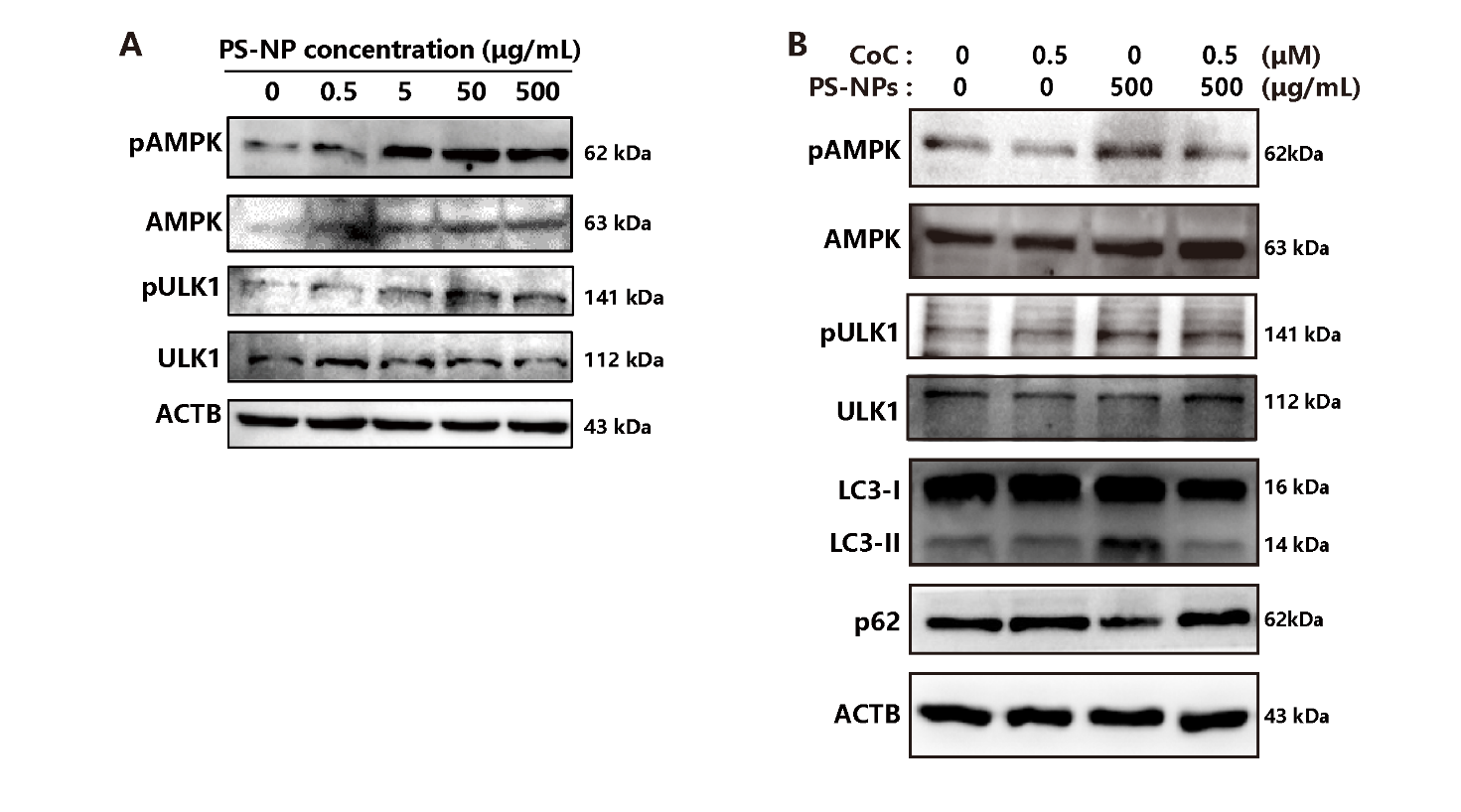


**Fig. S8.** Representative western blot gel images in Figure 5. (A) Western blot gels of pAMPK/AMPK and pULK1/ULK1 in differentiated SH-SY5Y cells treated with PS-NPs. (B) Western blot gels of pAMPK/AMPK, pULK1/ULK1, LC3-II/LC3-I and p62 in differentiated SH-SY5Y cells exposed to 500 μg/mL PS-NPs with or without 3-MA (100 μM).


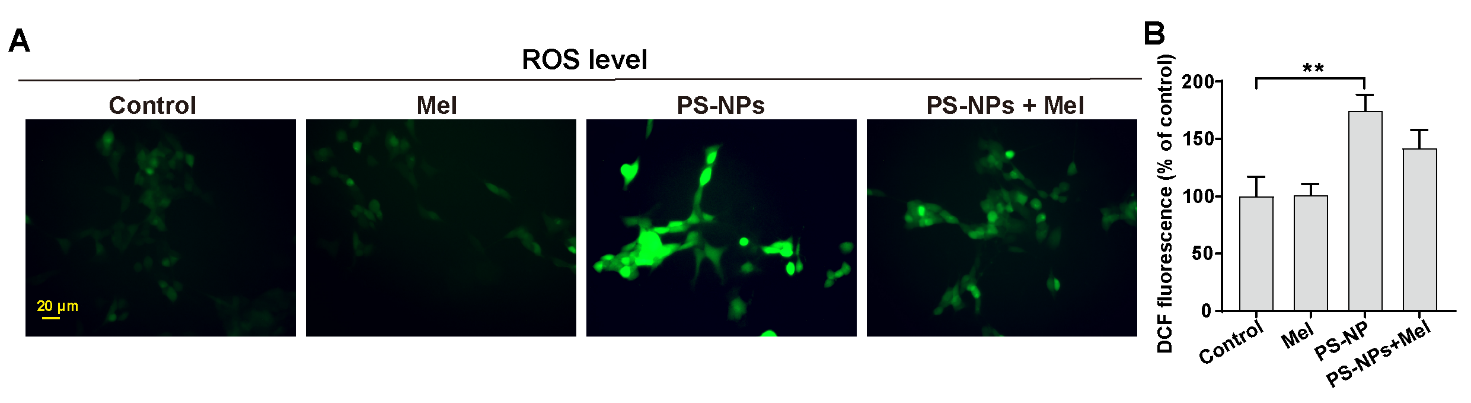


**Fig. S9.** The effect of melatonin on ROS levels in cells exposed to PS-NPs. Differentiated SH-SY5Y cells were treated with 500 μg/mL PS-NPs in the presence or absence of 16 μM Mel for 48 h and stained with 10 μM DCFH-DA. (A) Representative images. (B) Quantification of ROS levels. ** *P* < 0.01.


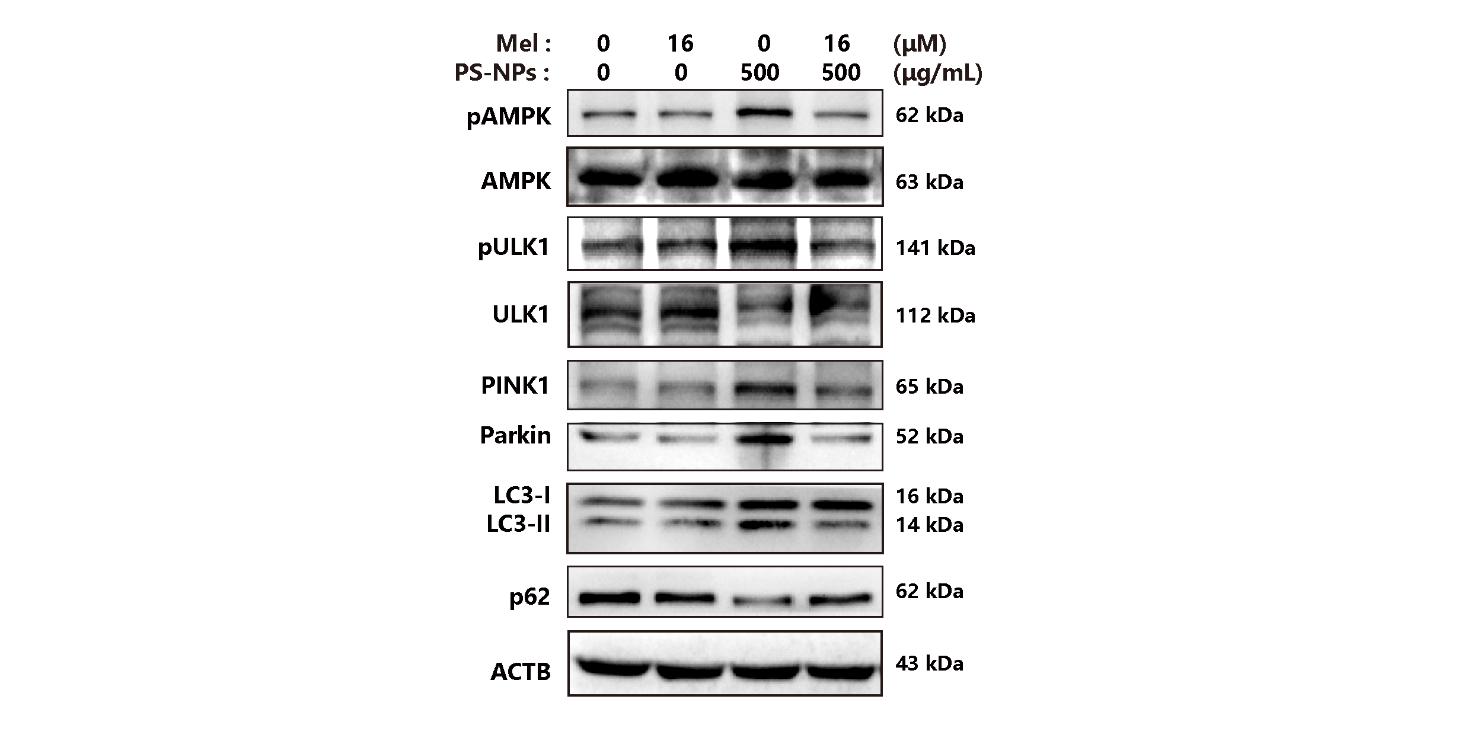


**Fig. S10.** Representative western blot gel images in Figure 6. Western blot gels of pAMPK/AMPK, pULK1/ULK1, LC3-II/LC3-I and p62 in differentiated SH-SY5Y cells exposed to 500 μg/mL PS-NPs with or without Mel (16 μM).


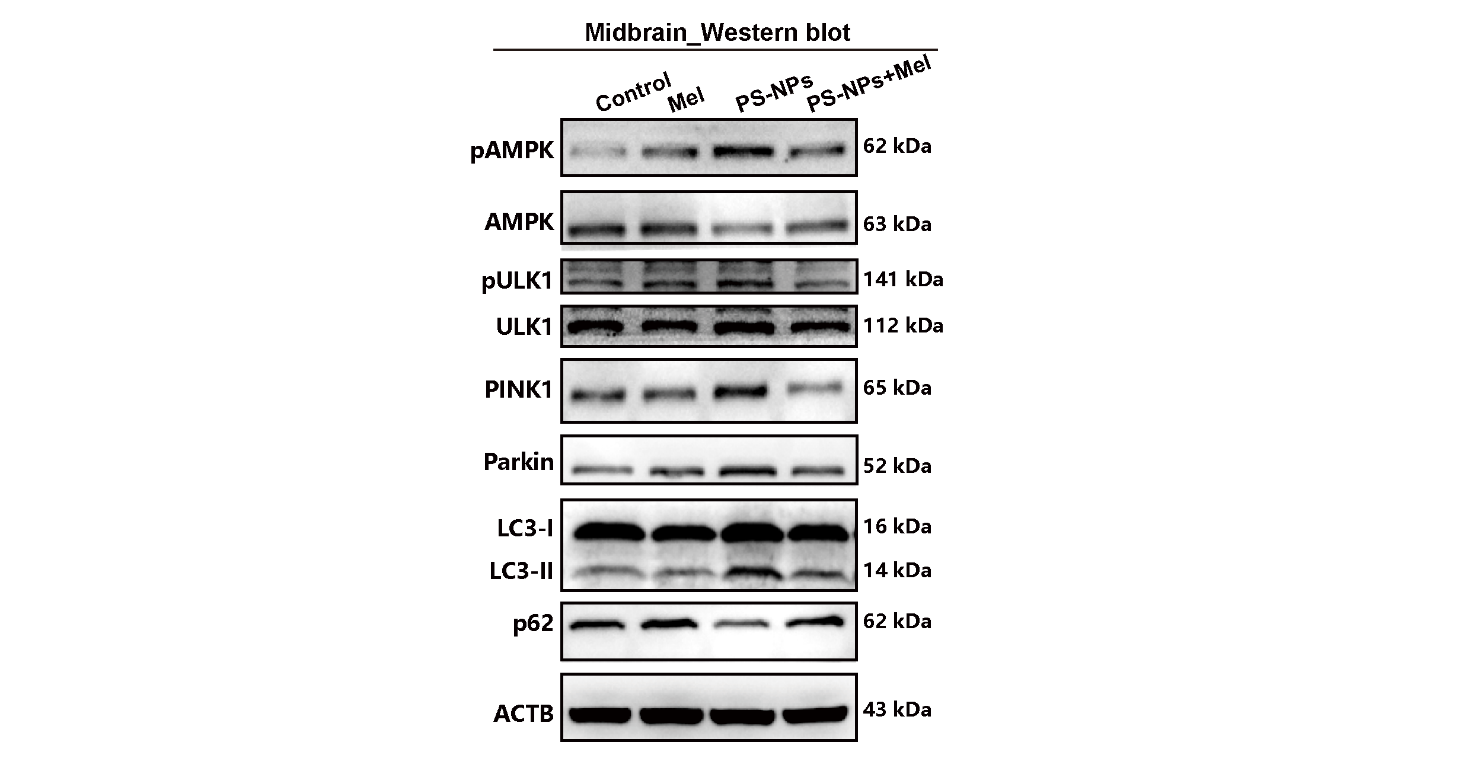


**Fig. S11.** Representative western blot gel images in Figure 7. Western blot gels of pAMPK/AMPK, pULK1/ULK1, LC3-II/LC3-I and p62 in mouse midbrain.

**
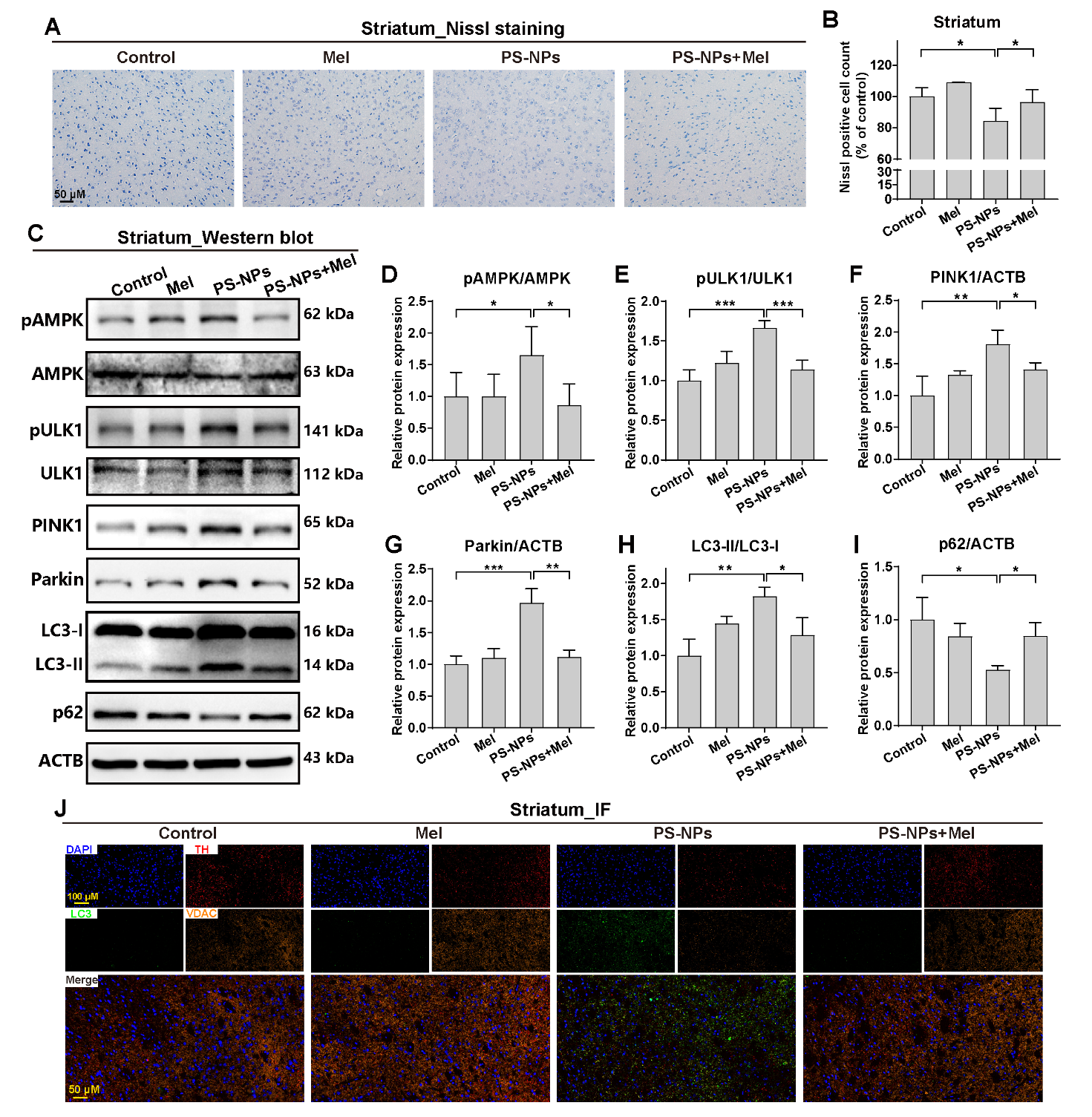
**

**Fig. S12.** Melatonin mitigating PS-NP-induced dopaminergic neuron loss in the striatum by modulating mitophagy. 250 mg/kg/day PS-NPs and 10 mg/kg/day melatonin were applied during 28-day exposure. (A-B) Representative images of Nissl staining and quantitative analyses in striatum neurons. (C-I) Detection and quantification of protein expression levels of pAMPK/AMPK, pULK1/ULK1, PINK1, Parkin, LC3-II/LC3-I, and p62 in mouse striatum. (J) Representative image of triple IF for LC3 (green), TH (red), VDAC (orange) and their merged images with DAPI (blue) in the striatum. Results are presented as mean ± SD (*n* = 5). * *P* < 0.05, ** *P* < 0.01, ** **P* < 0.001, compared to the indicative group.
