# Supplementary material for: Polystyrene nanoplastic exposure induces excessive mitophagy by activating AMPK/ULK1 pathway in differentiated SH-SY5Y cells and dopaminergic neurons in vivo
Source: Part Fibre Toxicol. 2023 Nov 22;20:44. doi: 10.1186/s12989-023-00556-4 (PMC10664492; doi:10.1186/s12989-023-00556-4)
Supplement: Supplementary file 2 — Additional file 2. Original bands of Western blots used in this study. [file 12989_2023_556_MOESM2_ESM.docx]

**Figure S2**

**PS-NP concentration (μg/mL)**

**0**

**0.5**

**5**

**50**

**500**

**PS-NP concentration (μg/mL)**

**0**

**0.5**

**5**

**50**

**500**

**PS-NP concentration (μg/mL)**

**0**

**0.5**

**5**

**50**

**500**


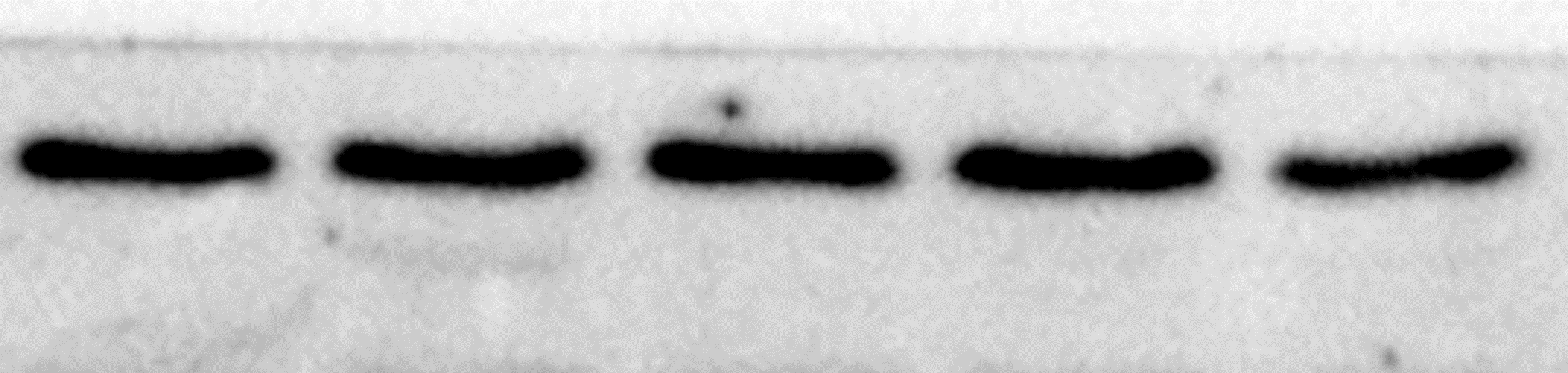

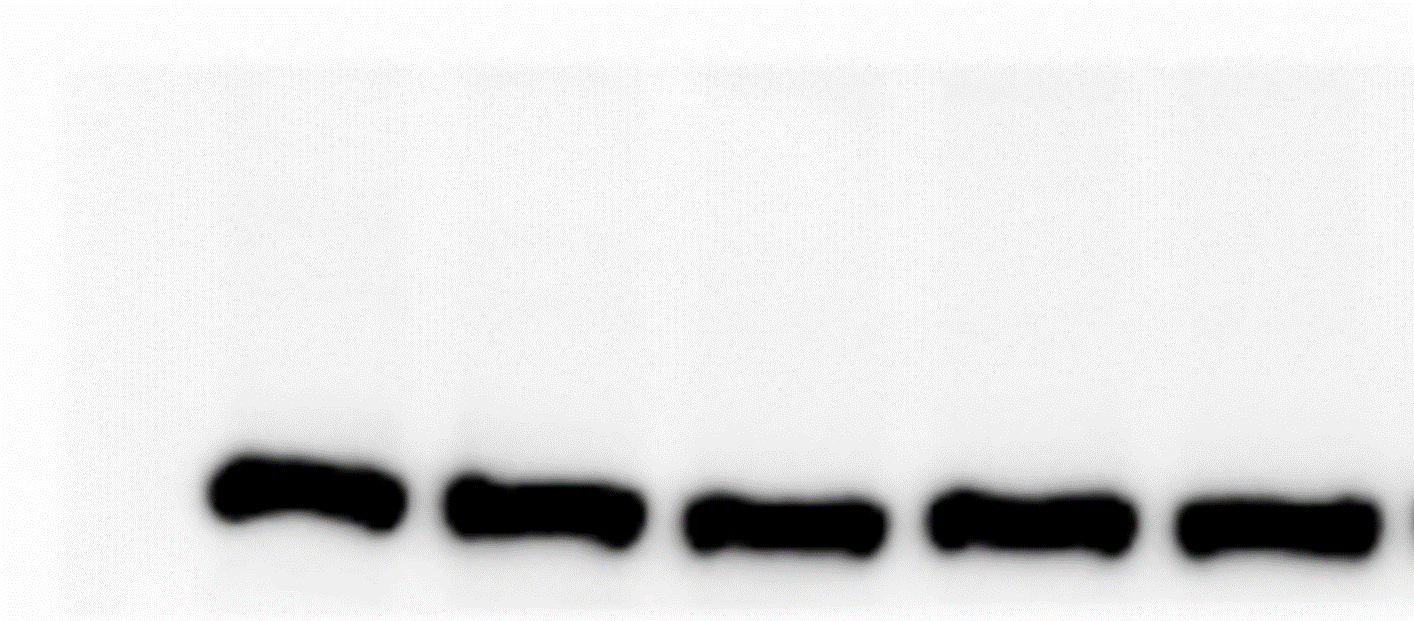

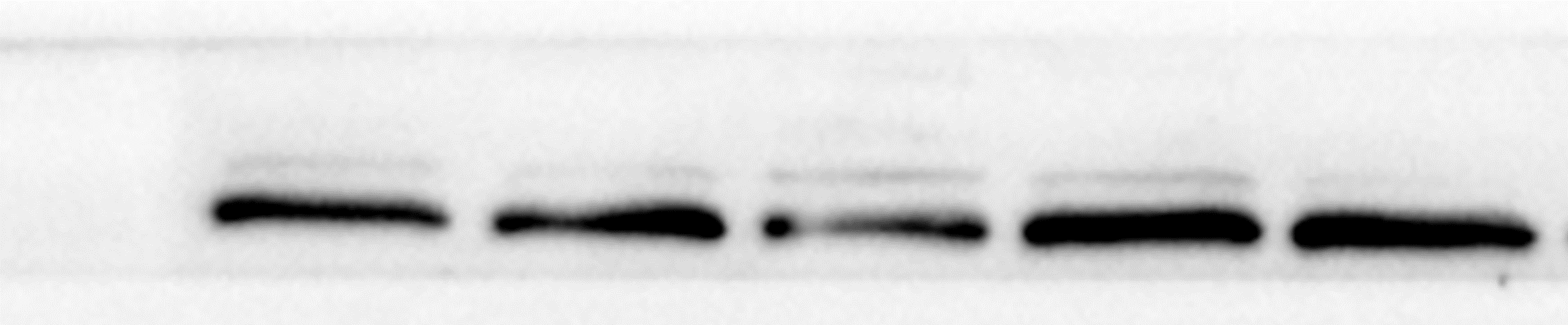

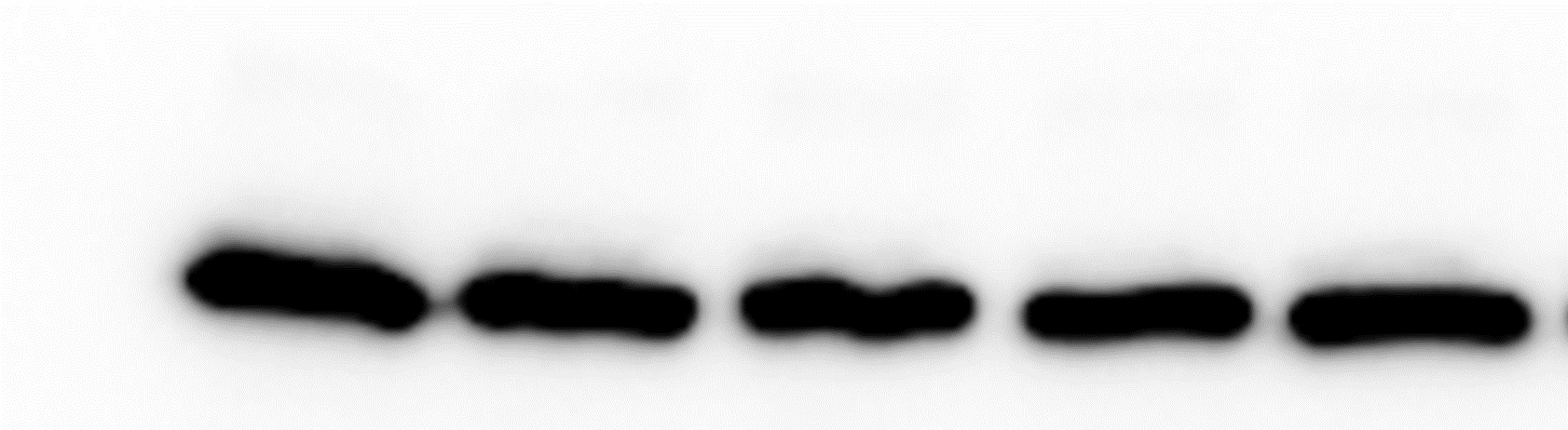

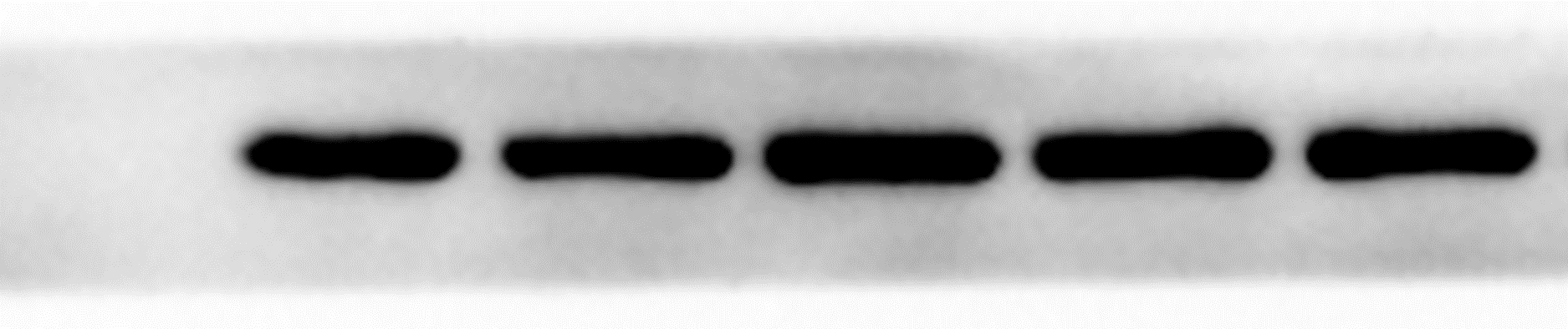

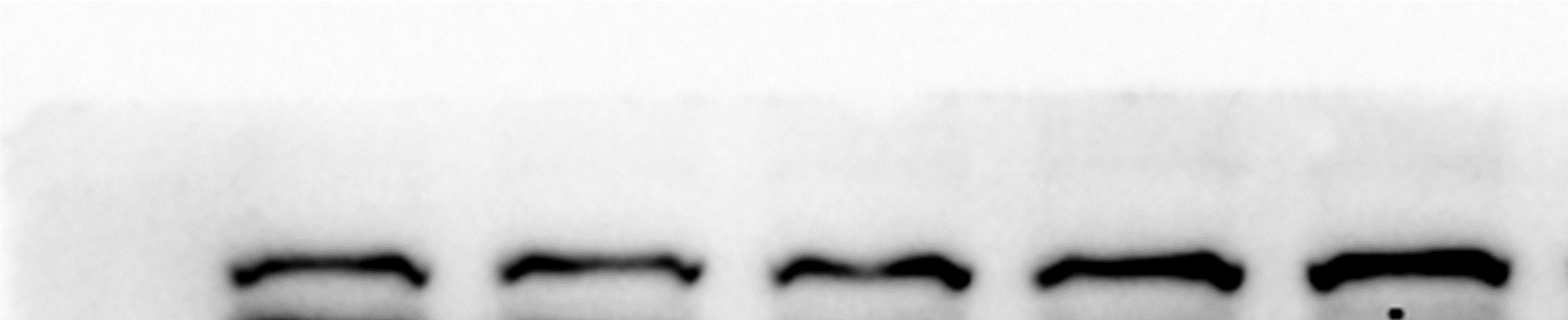

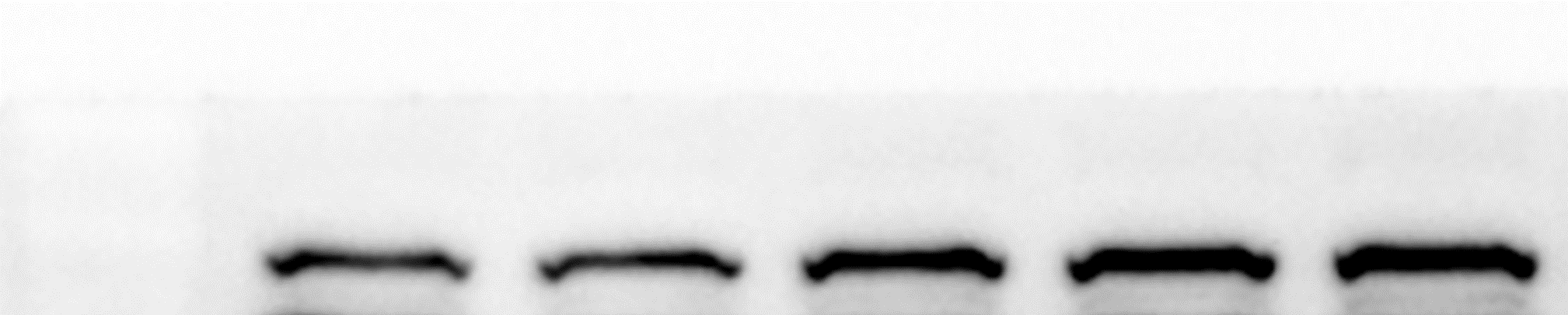

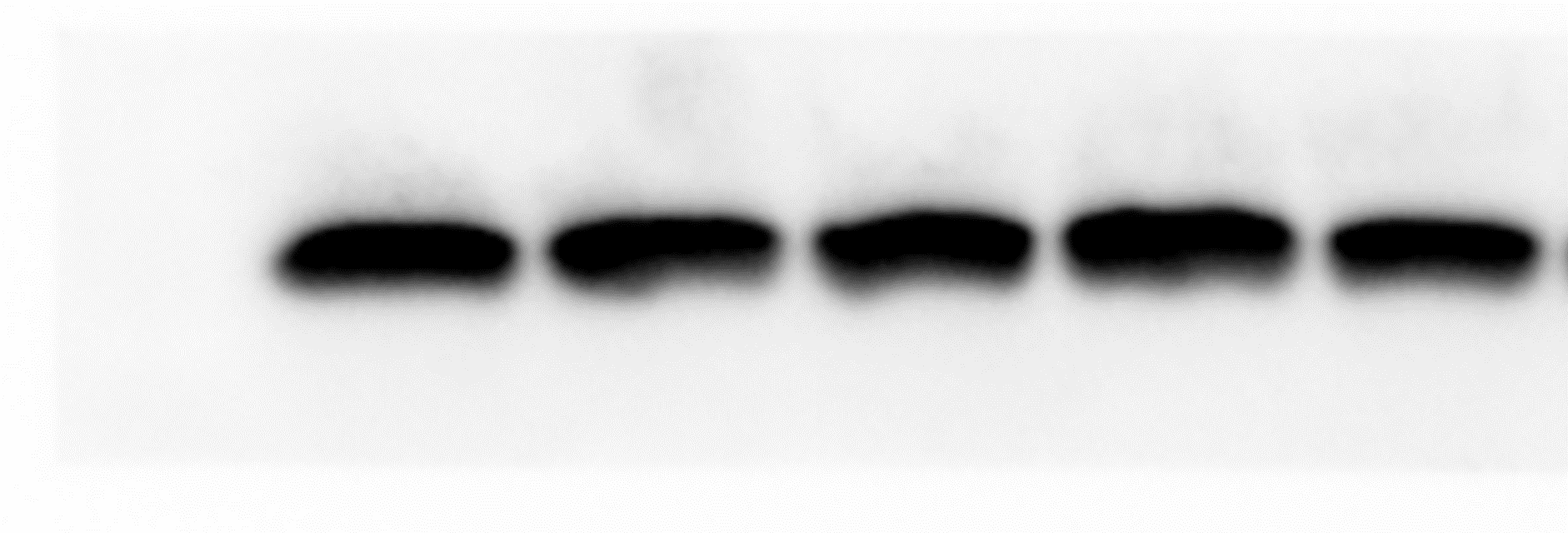

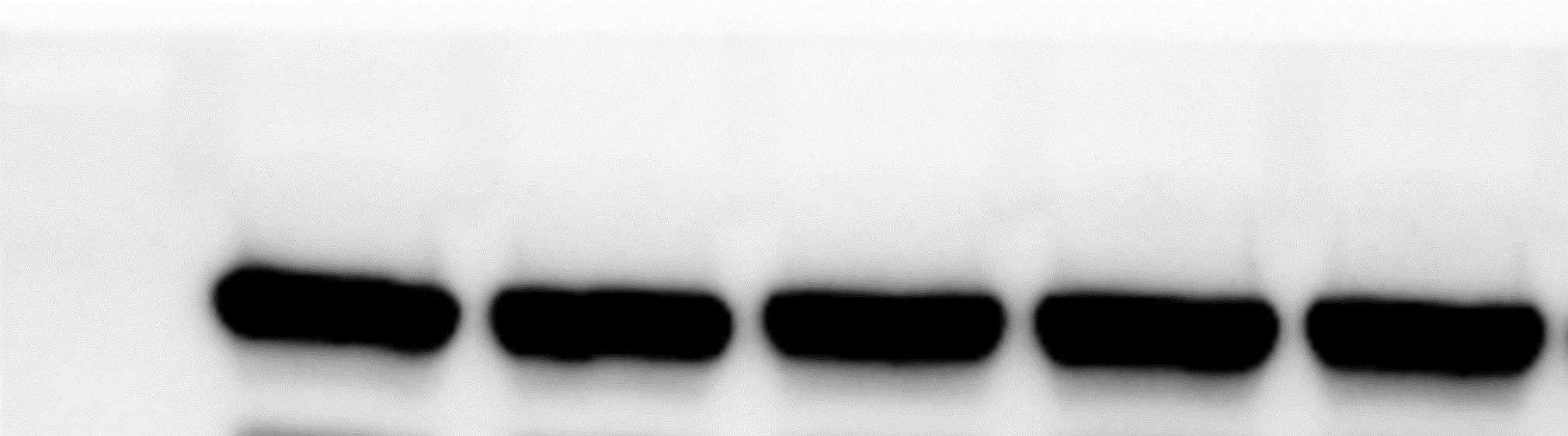

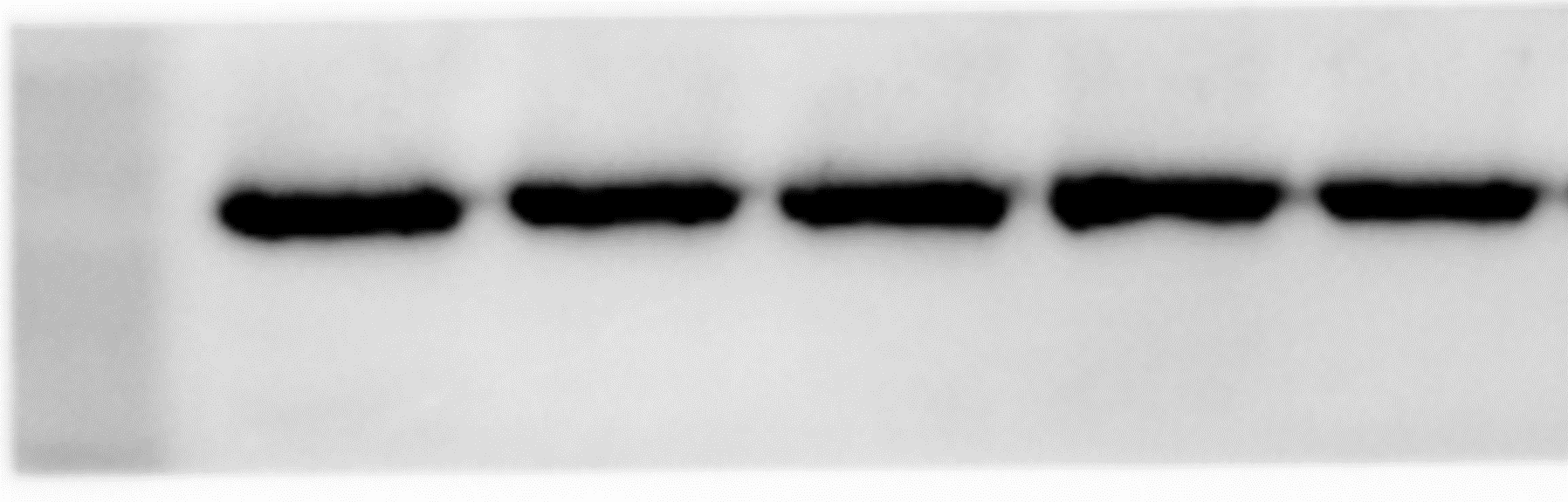

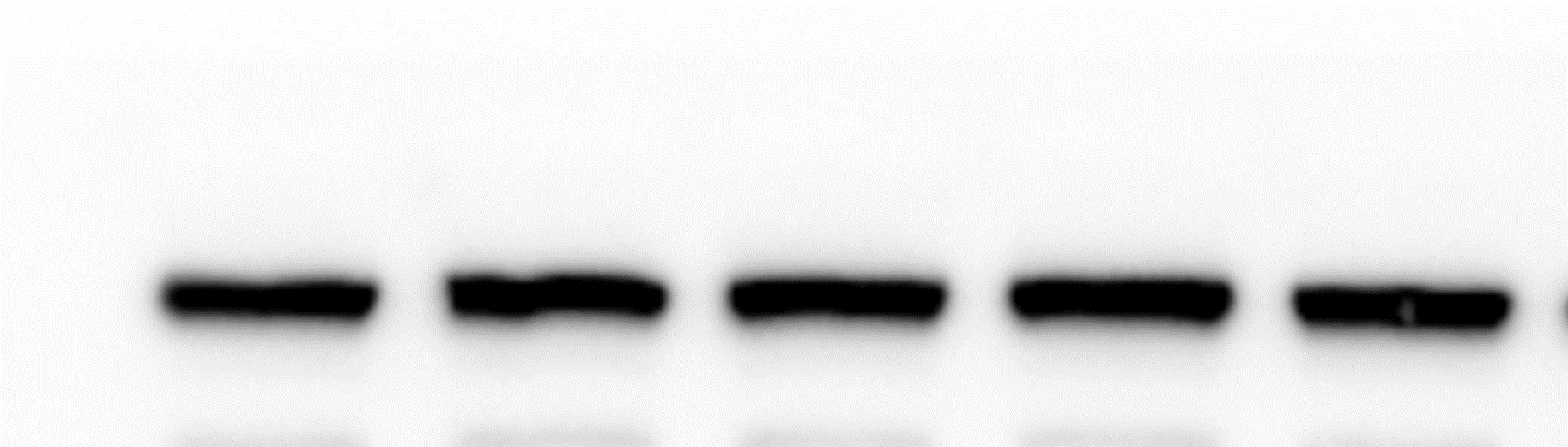

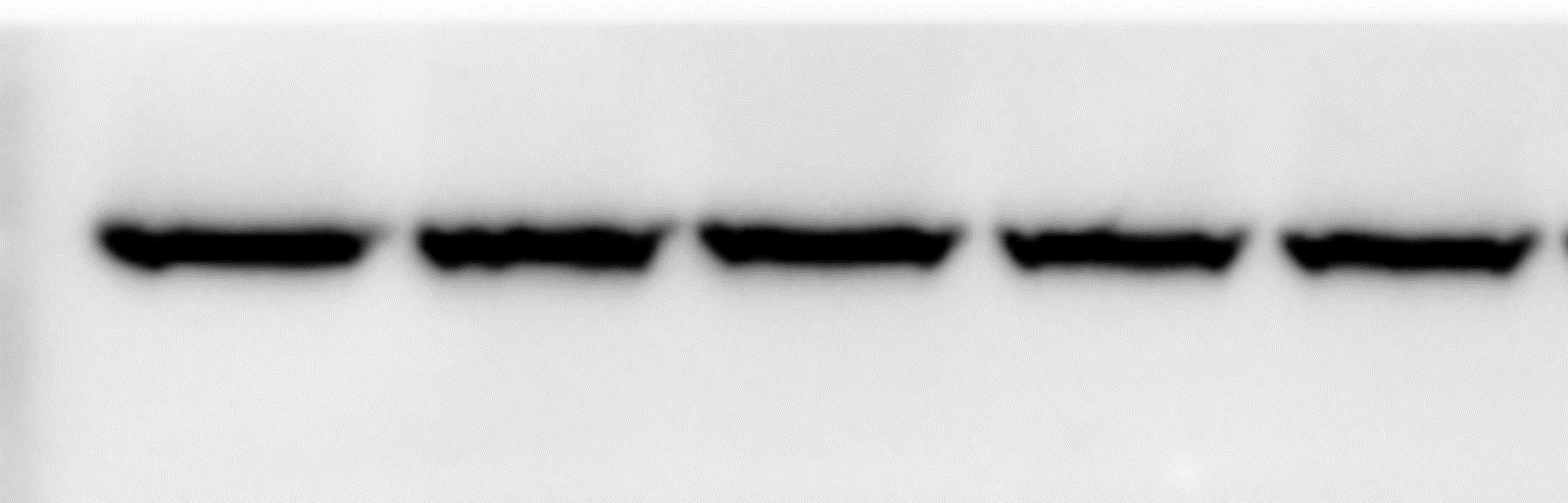

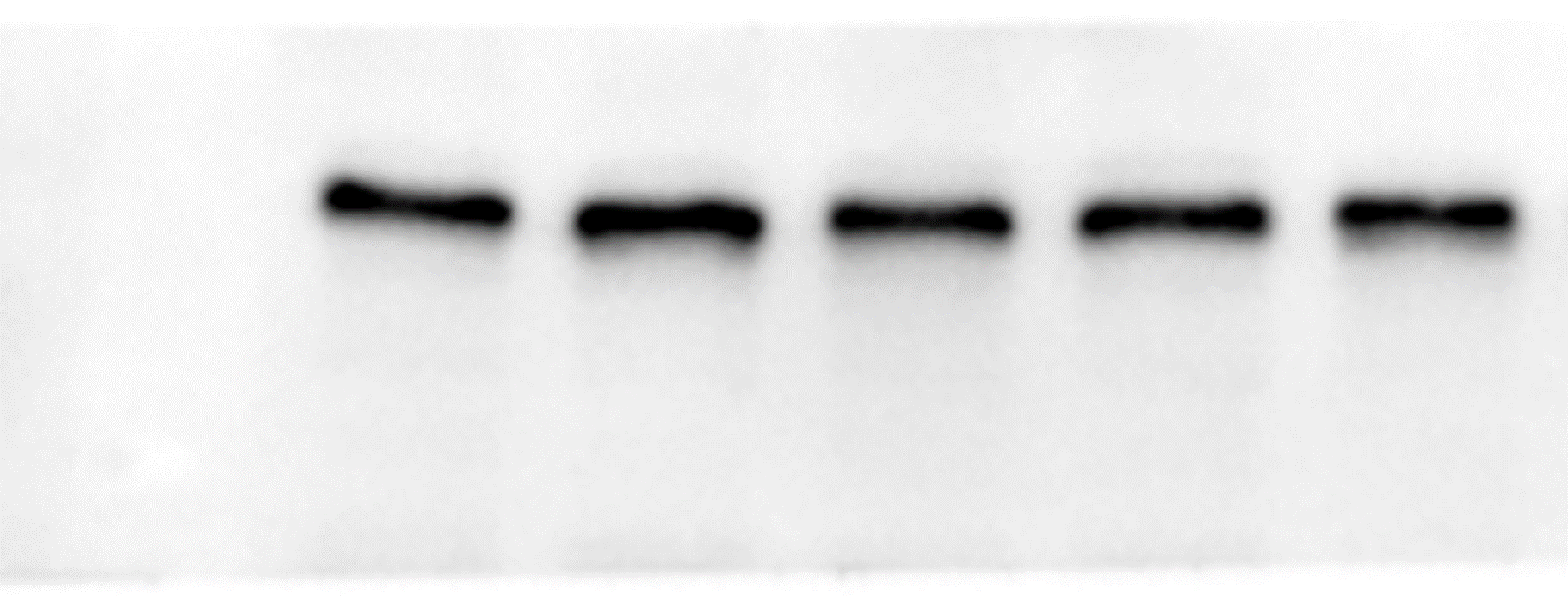

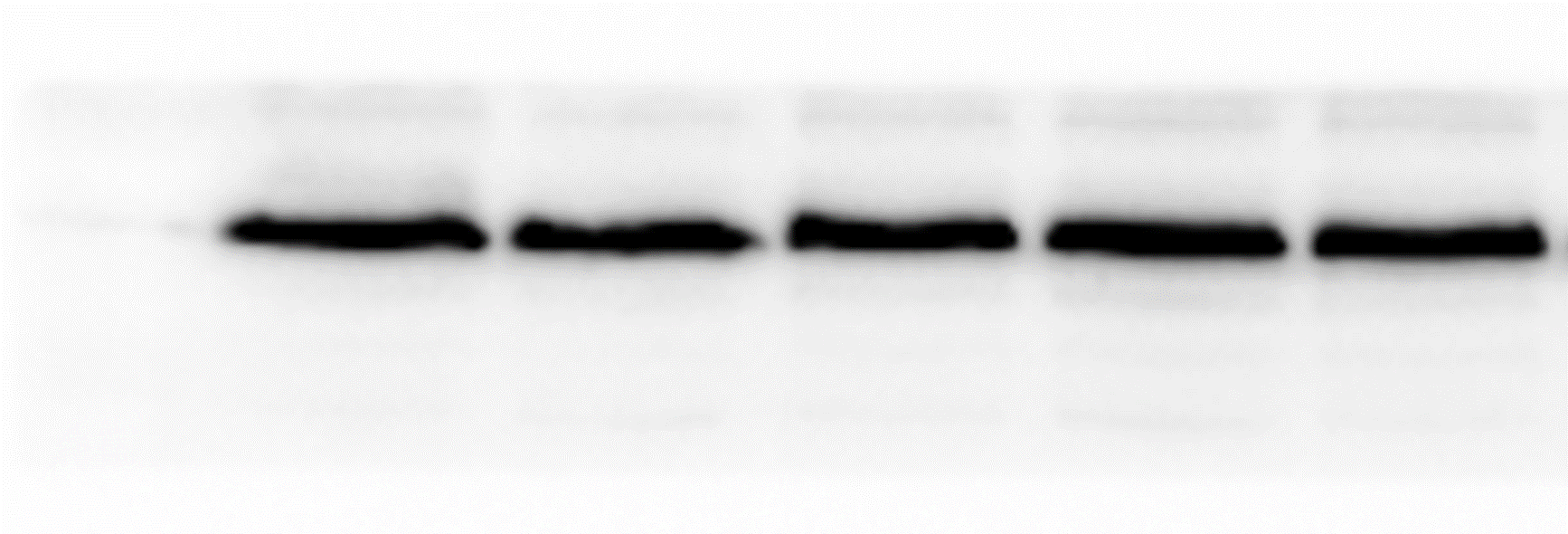

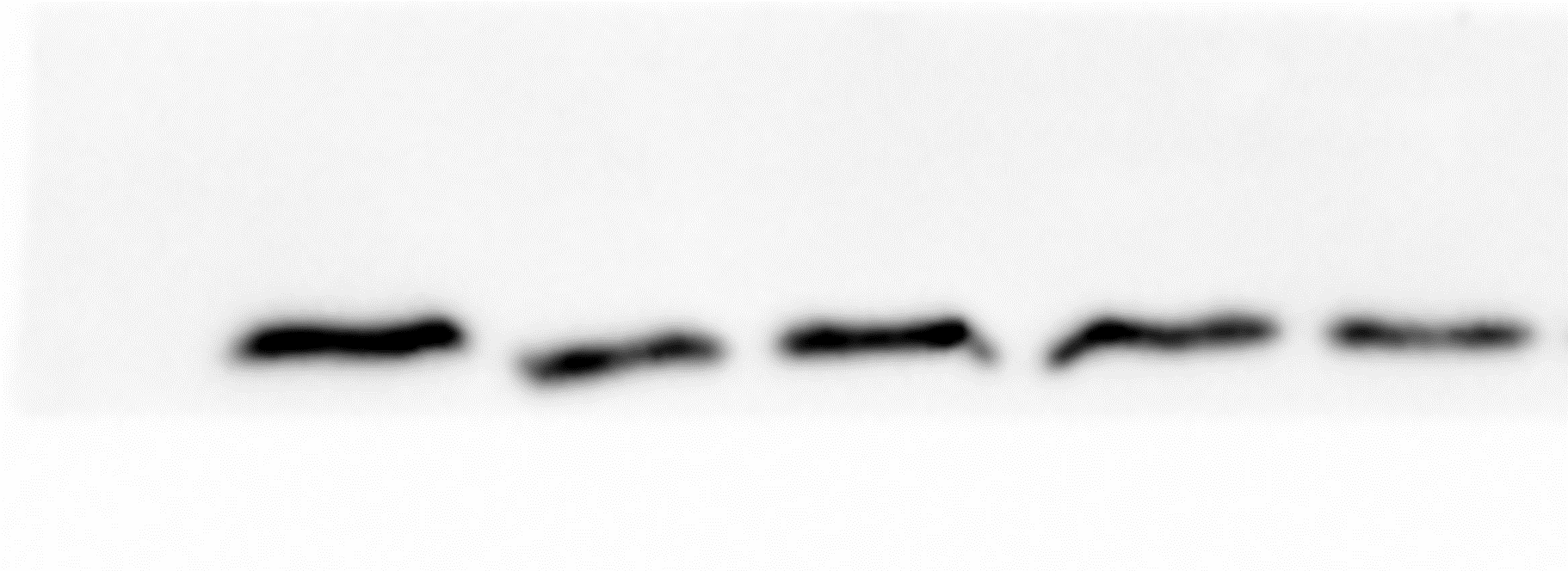

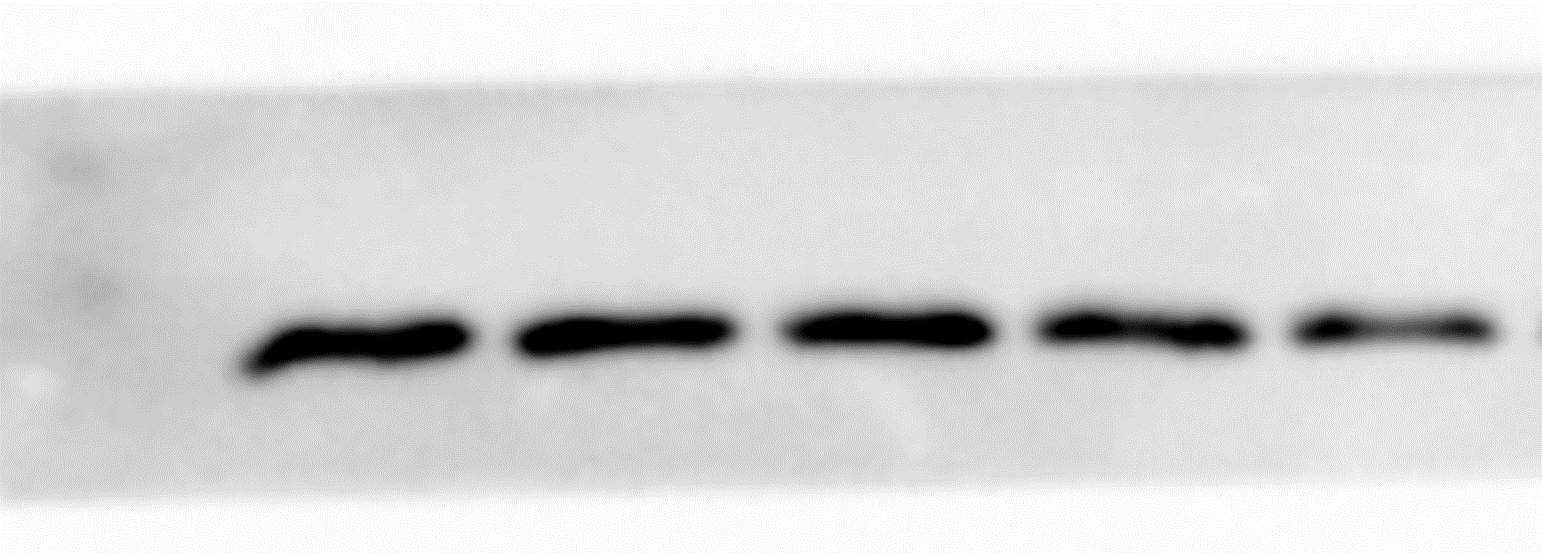

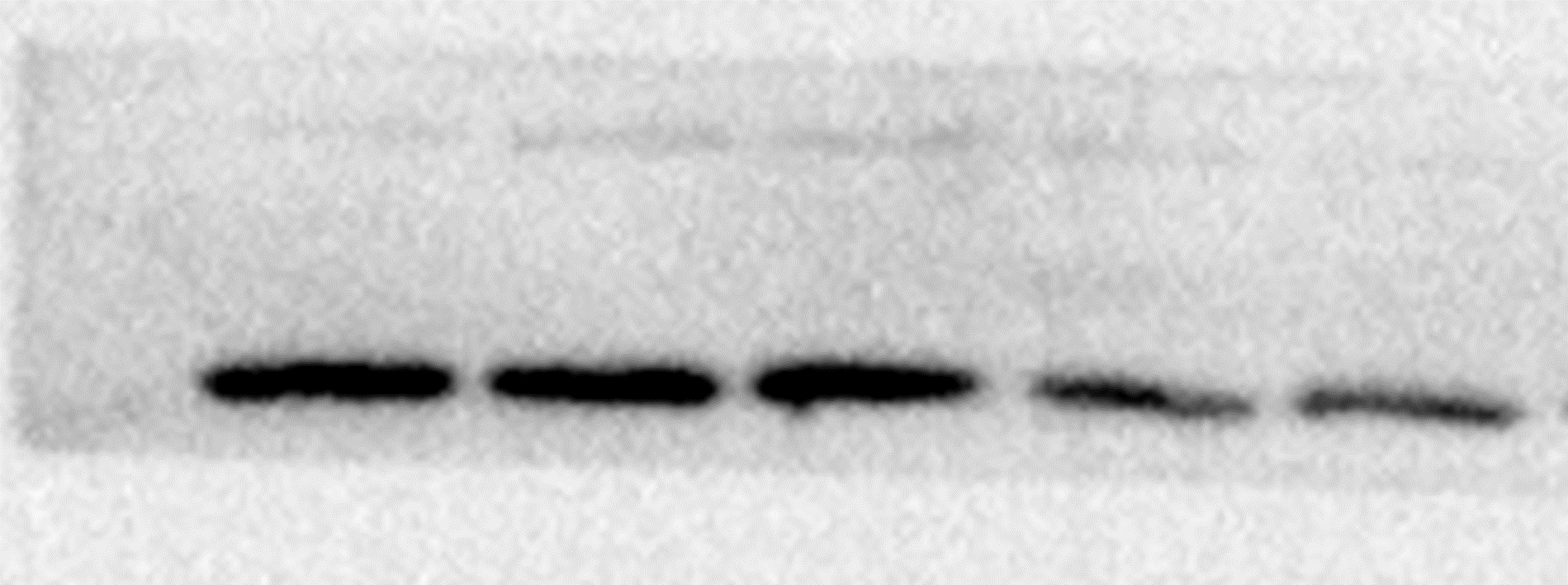

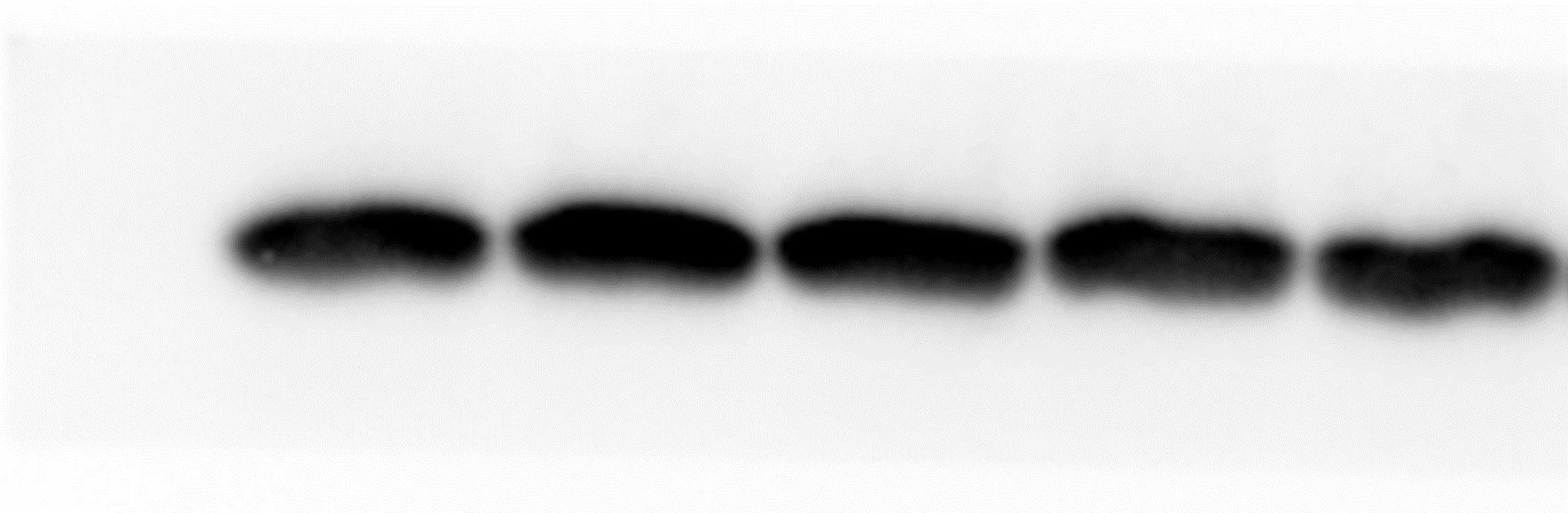


**SDHB**

**NDUFB8**

**MTCO2**

**UQCRC2**

**ATP5A**

**ACTB**

**Figure S4A**

**PS-NP concentration (μg/mL)**

**0**

**0.5**

**5**

**50**

**500**

**PS-NP concentration (μg/mL)**

**0**

**0.5**

**5**

**50**

**500**

**PS-NP concentration (μg/mL)**

**0**

**0.5**

**5**

**50**

**500**


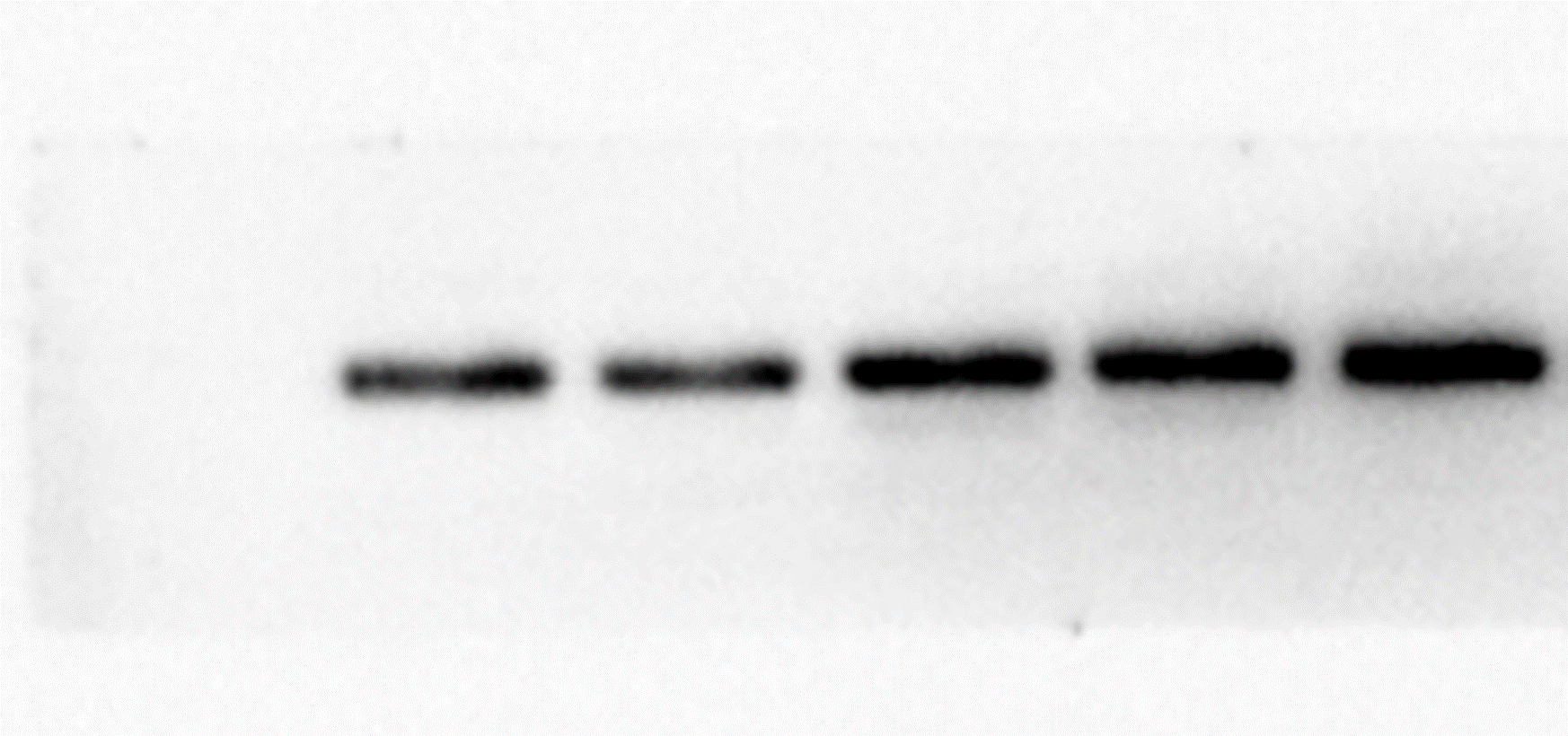

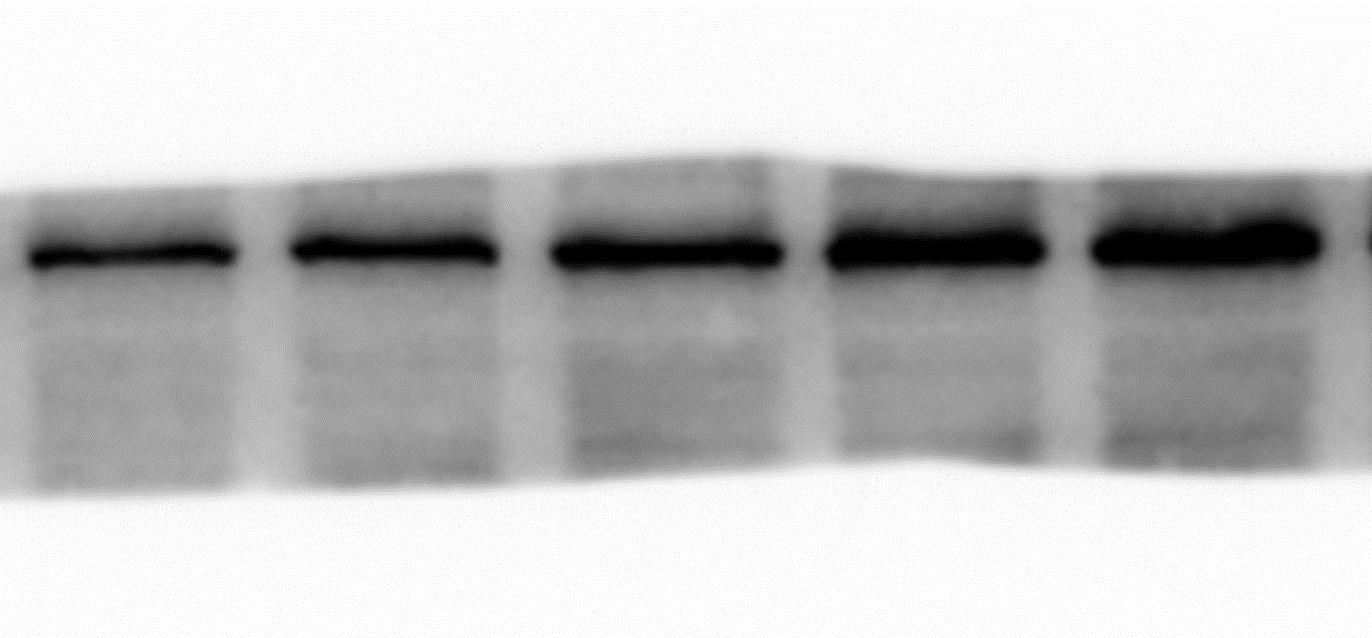

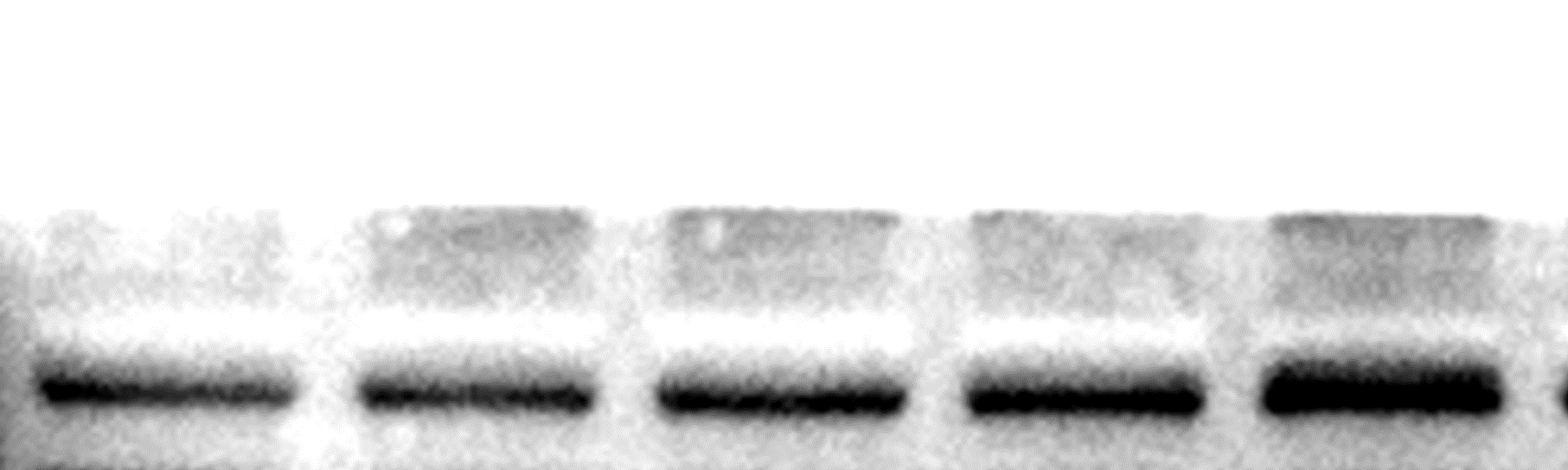


**PINK1**


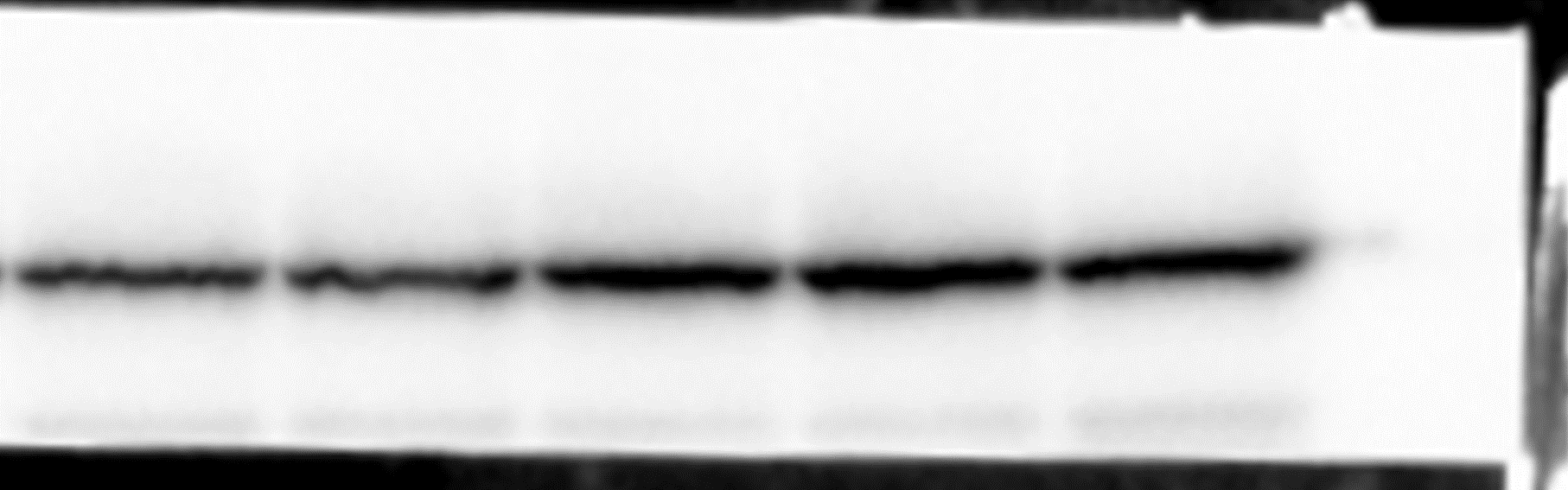


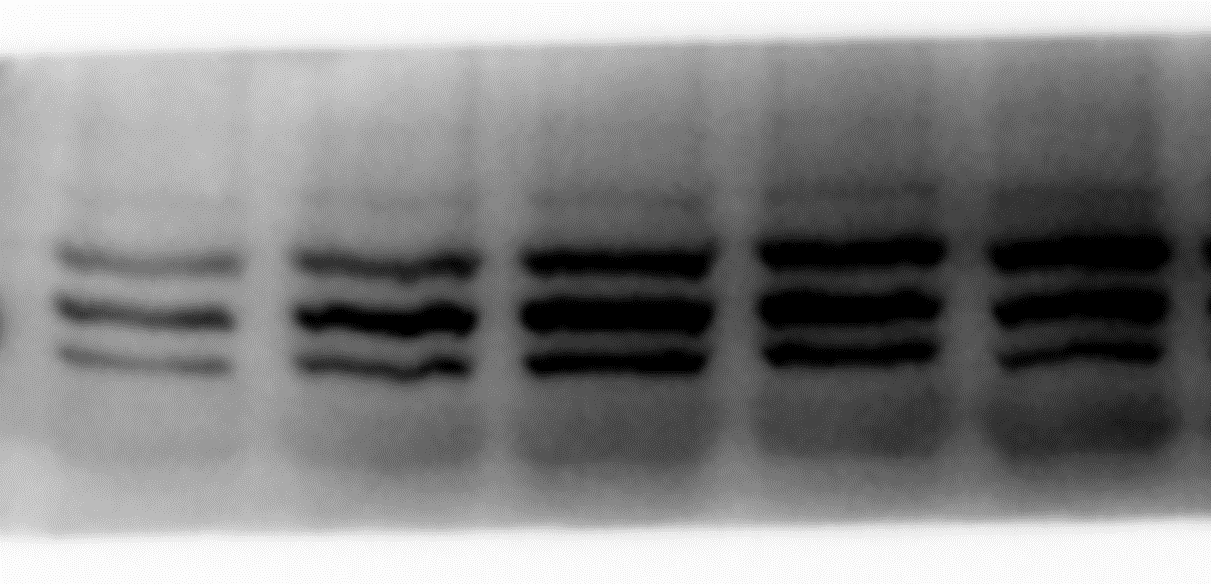

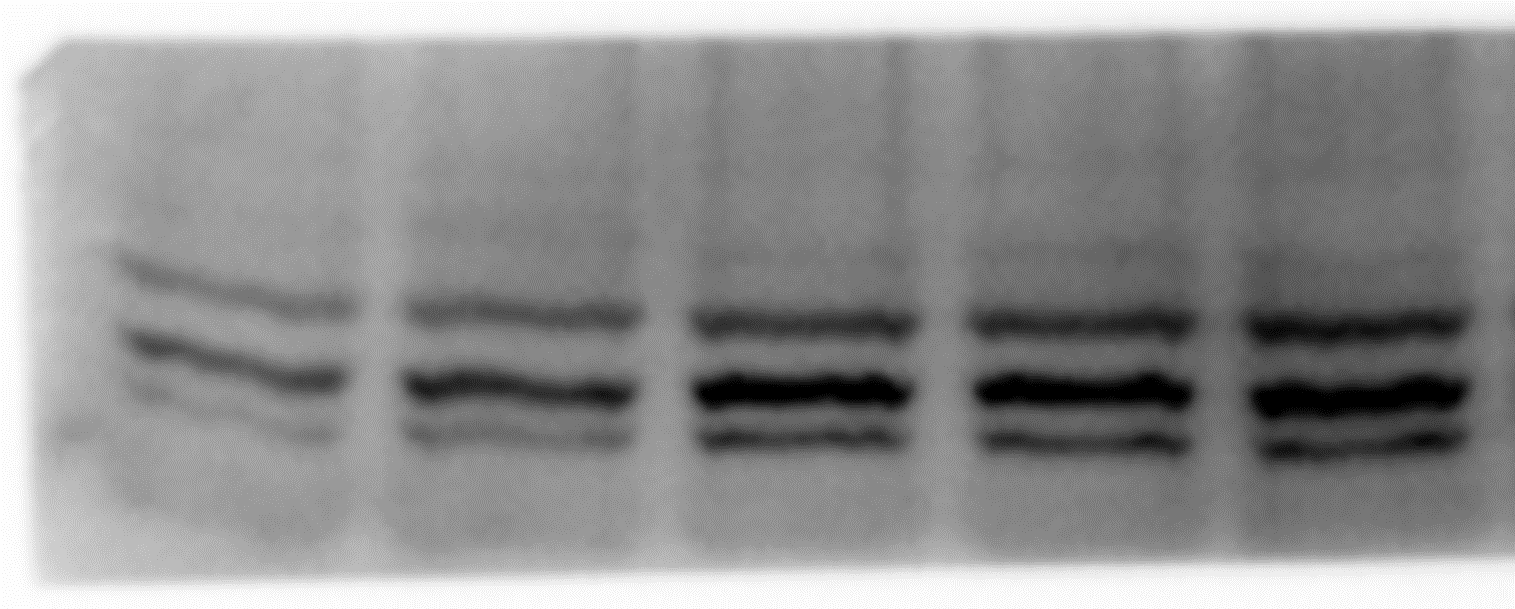


**Parkin**

**LC3-I**


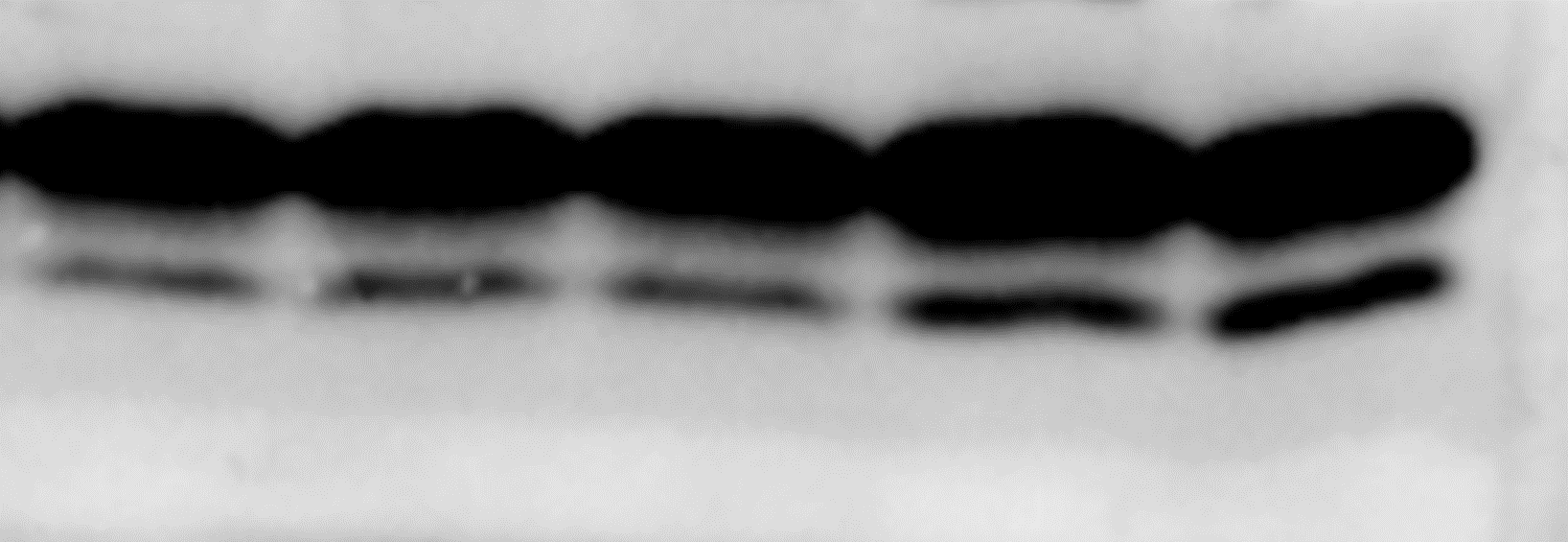

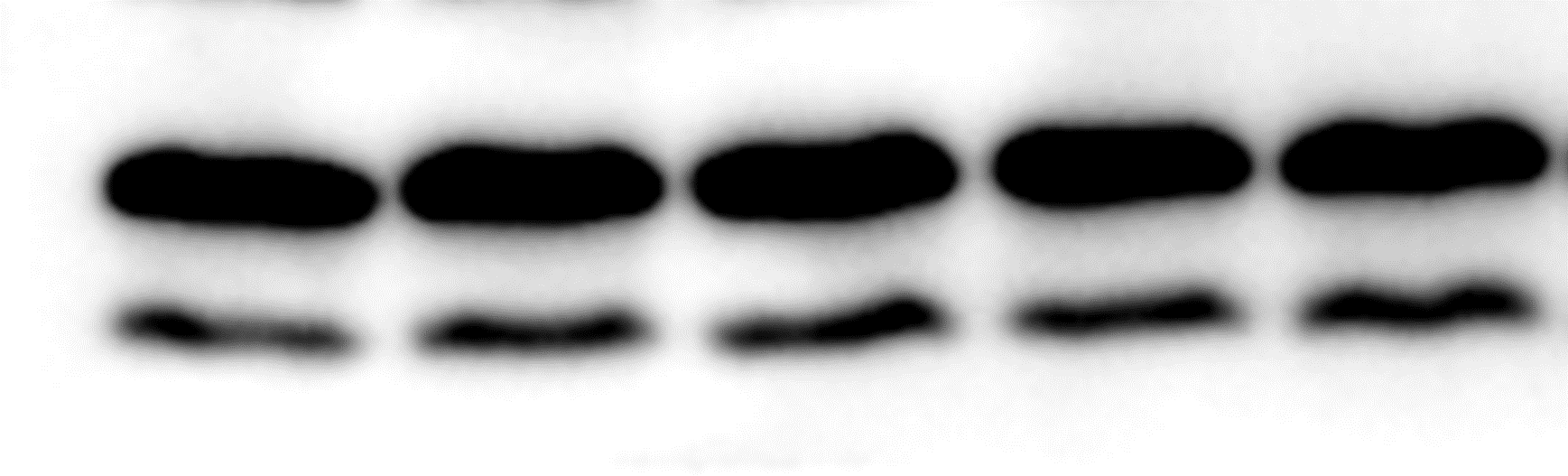

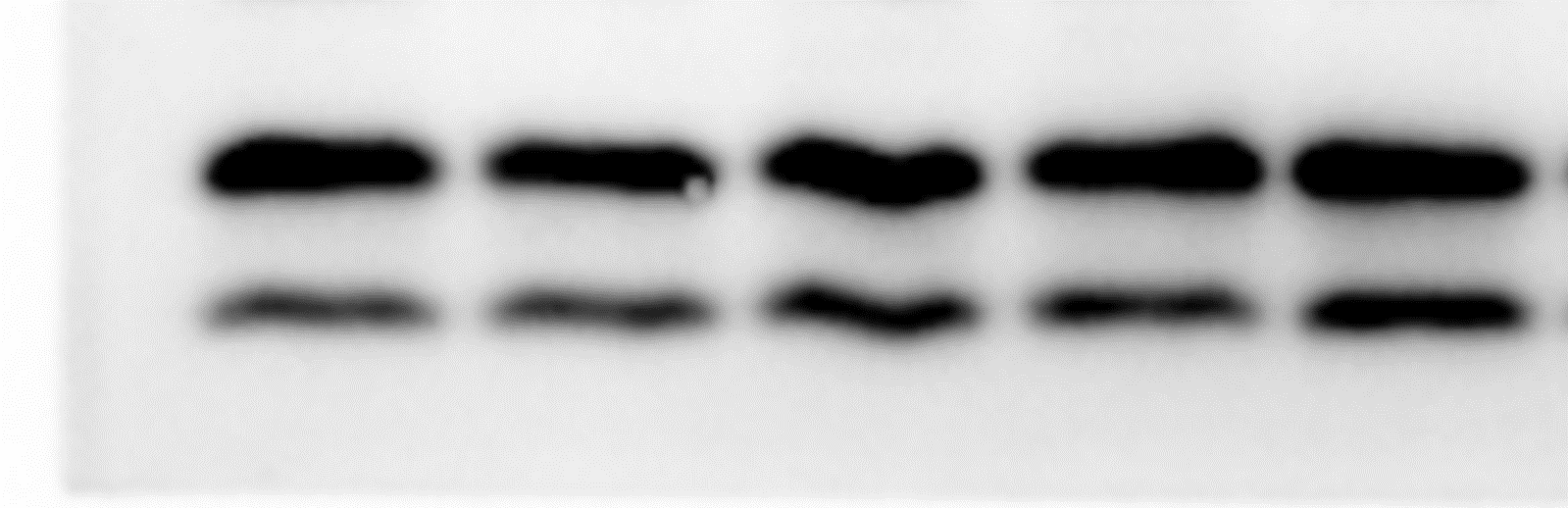


**LC3-II**


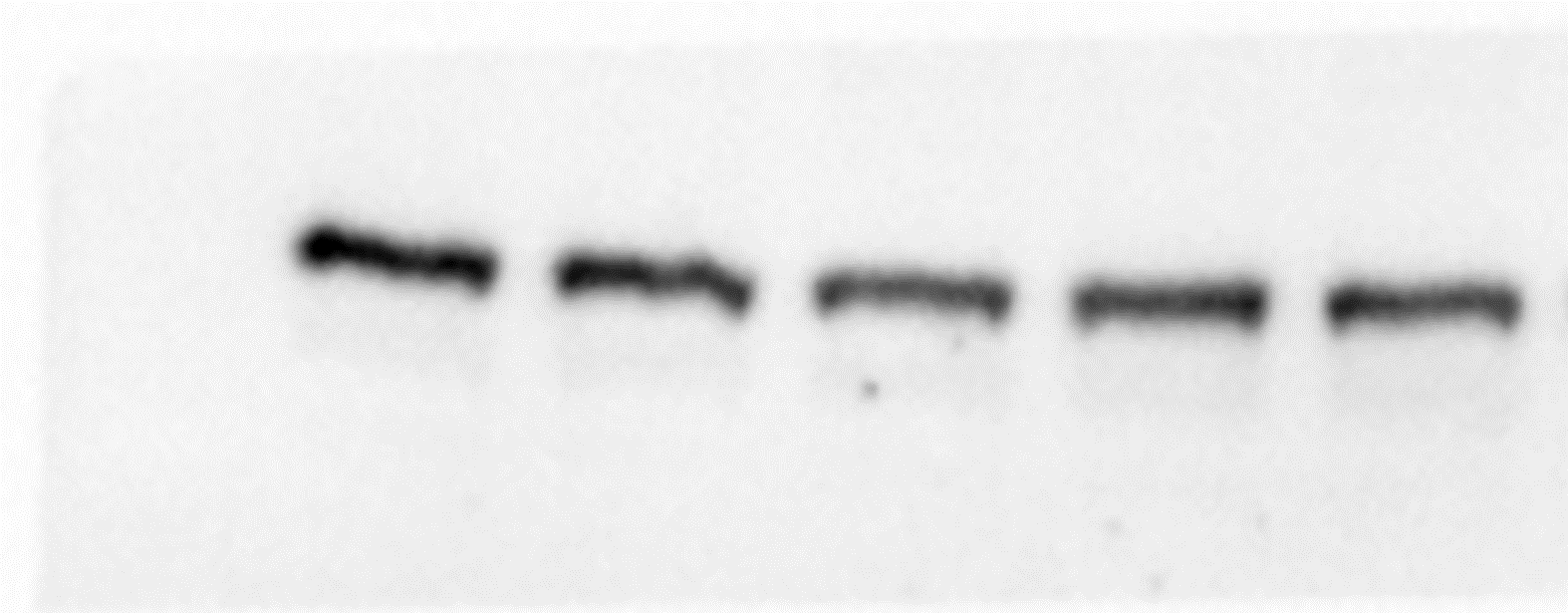

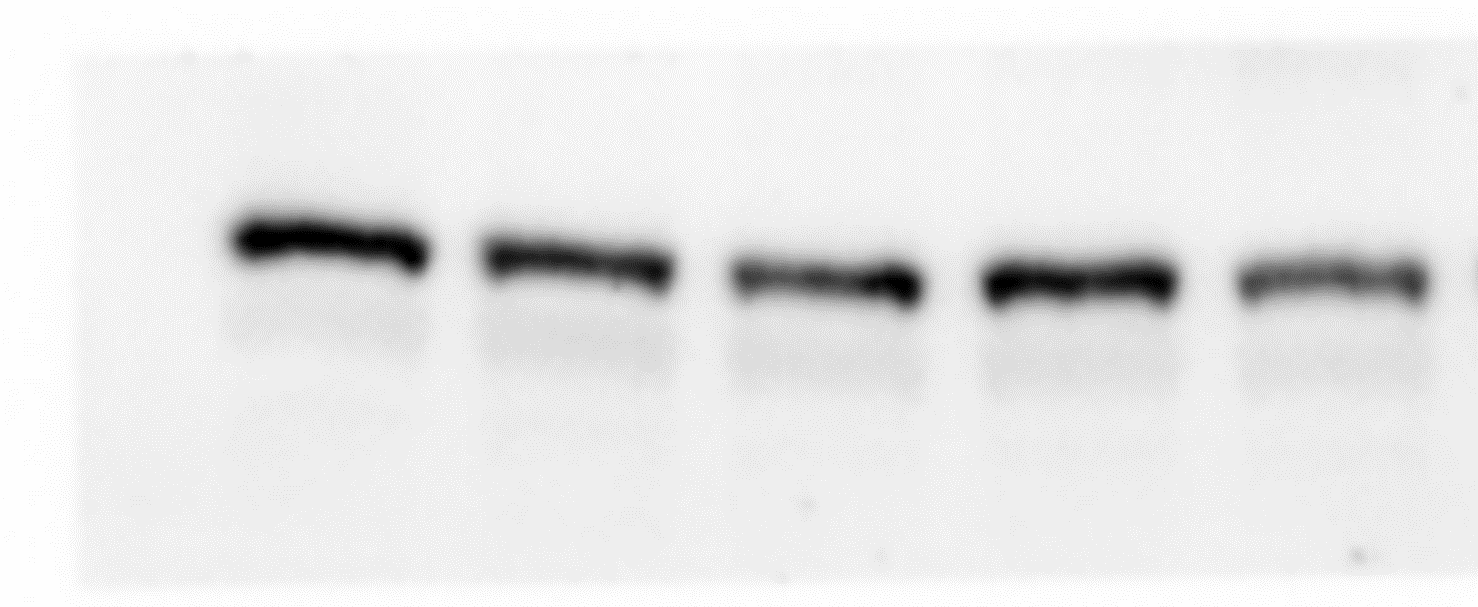


**p62**


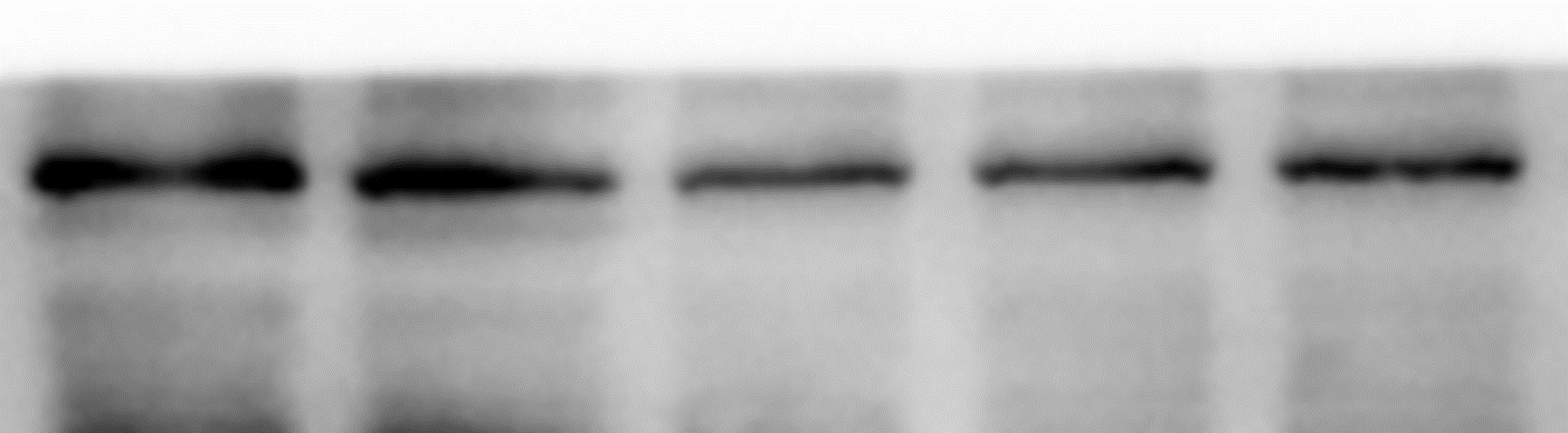


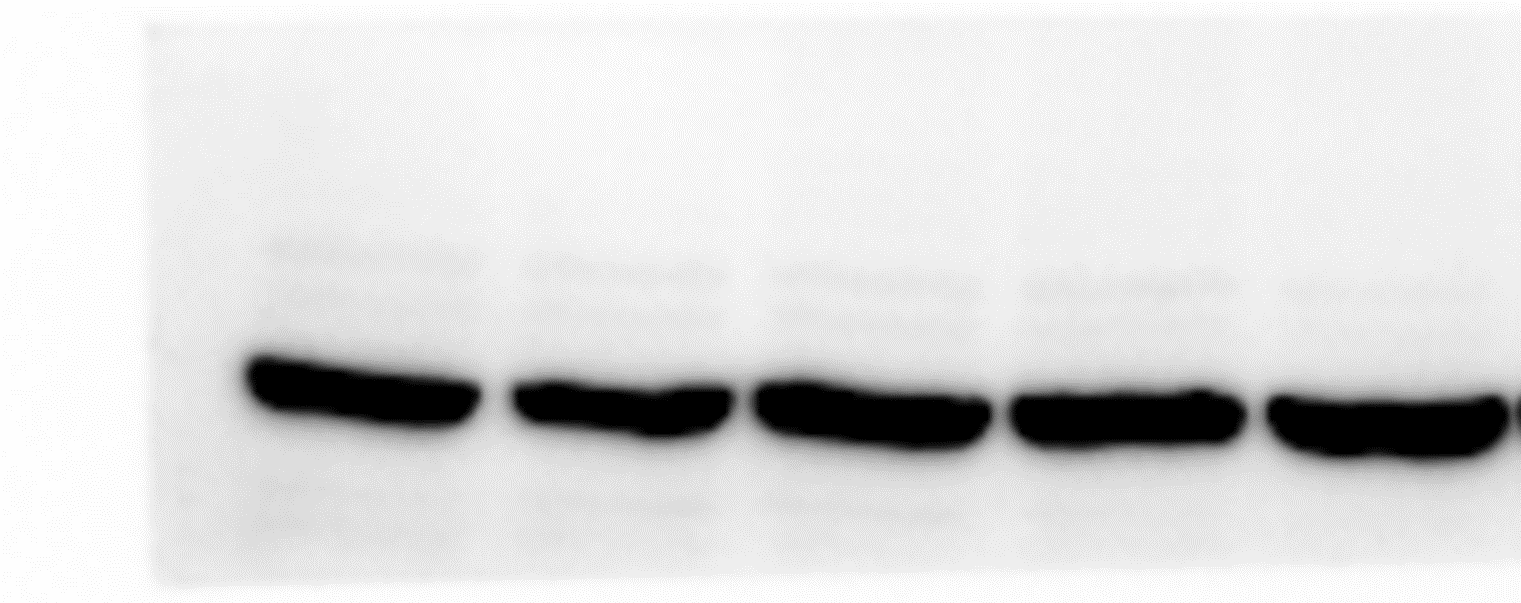

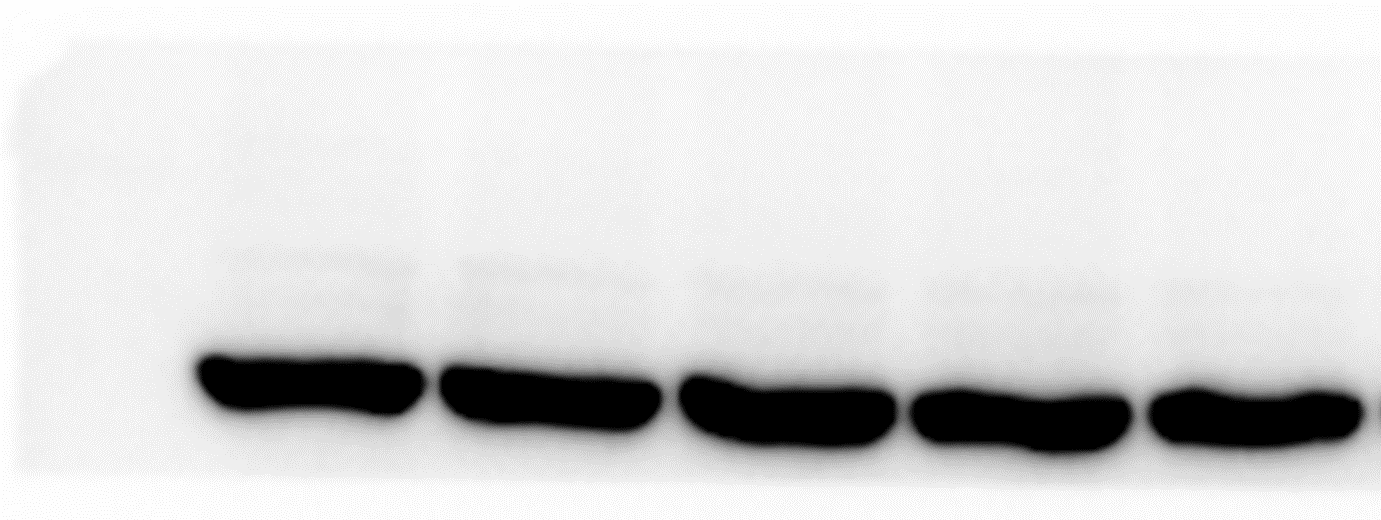


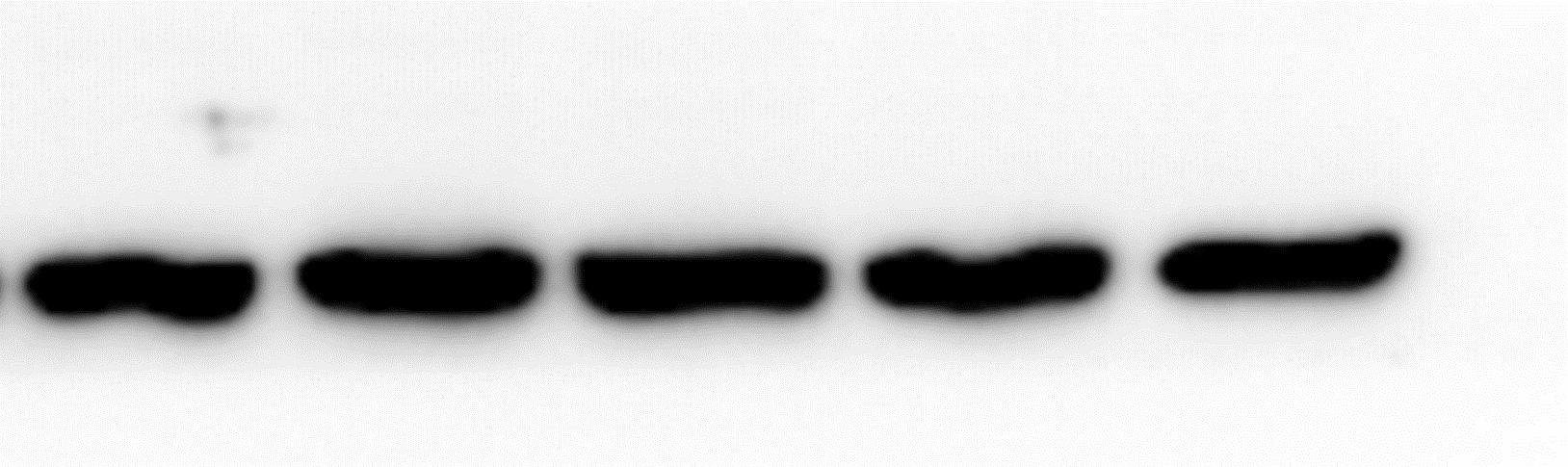


**ACTB**

**Figure S4B**


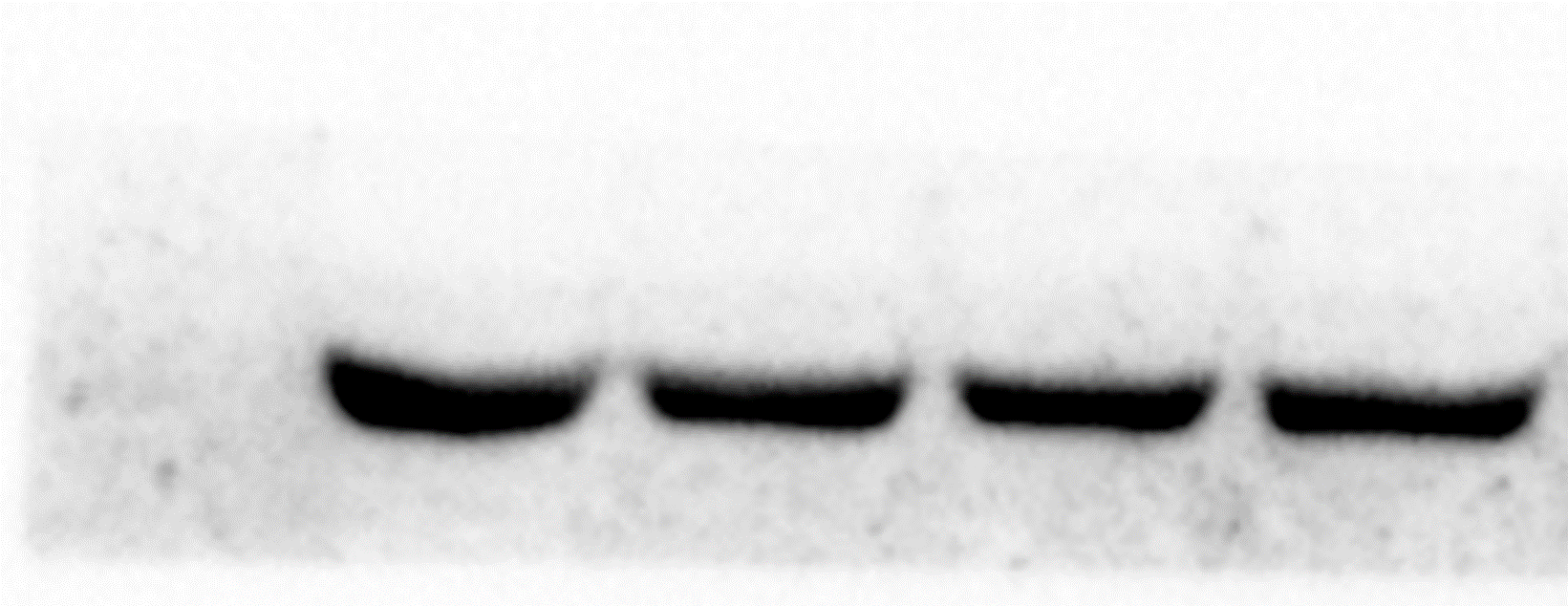

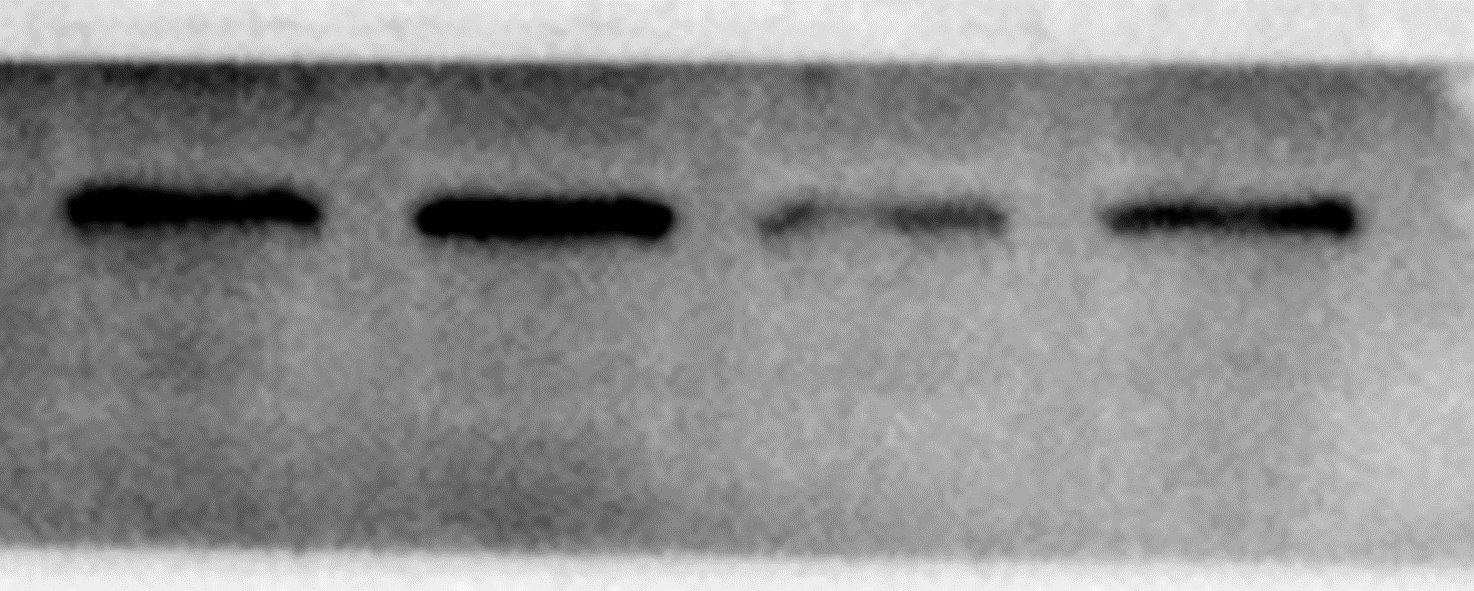

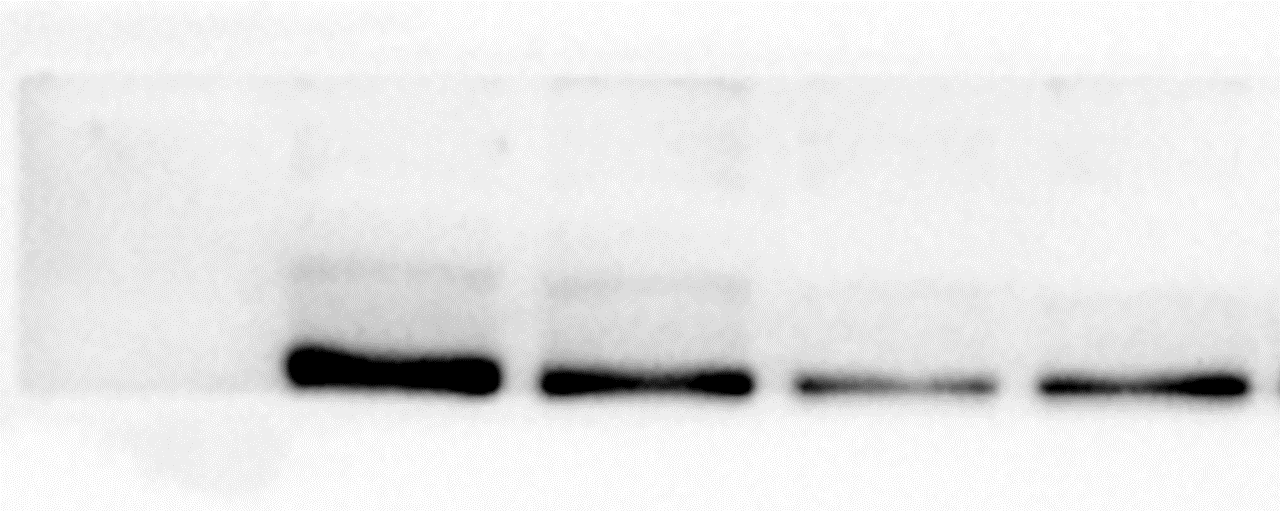

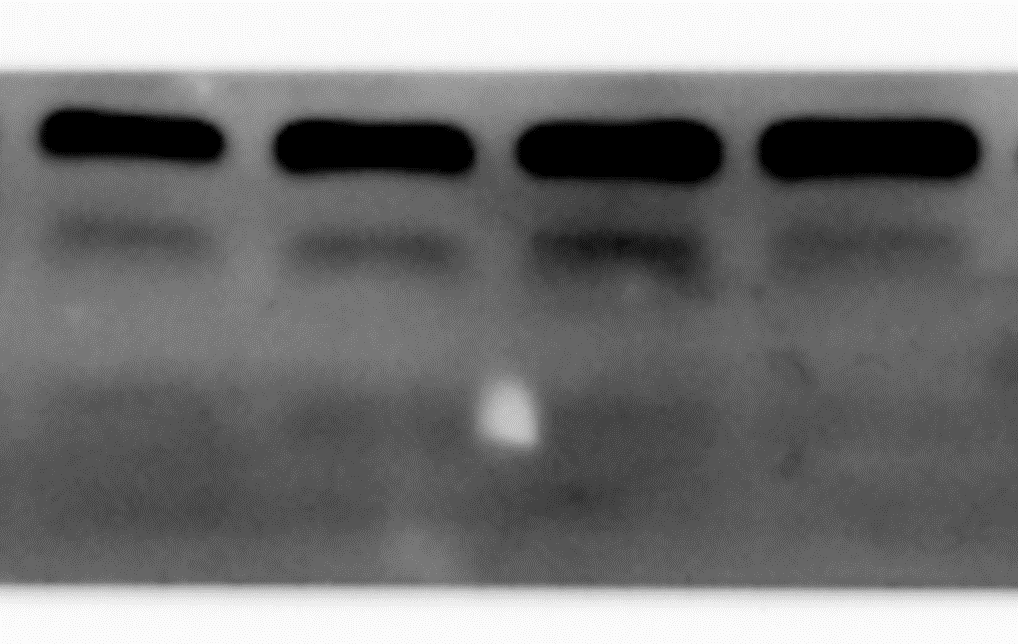

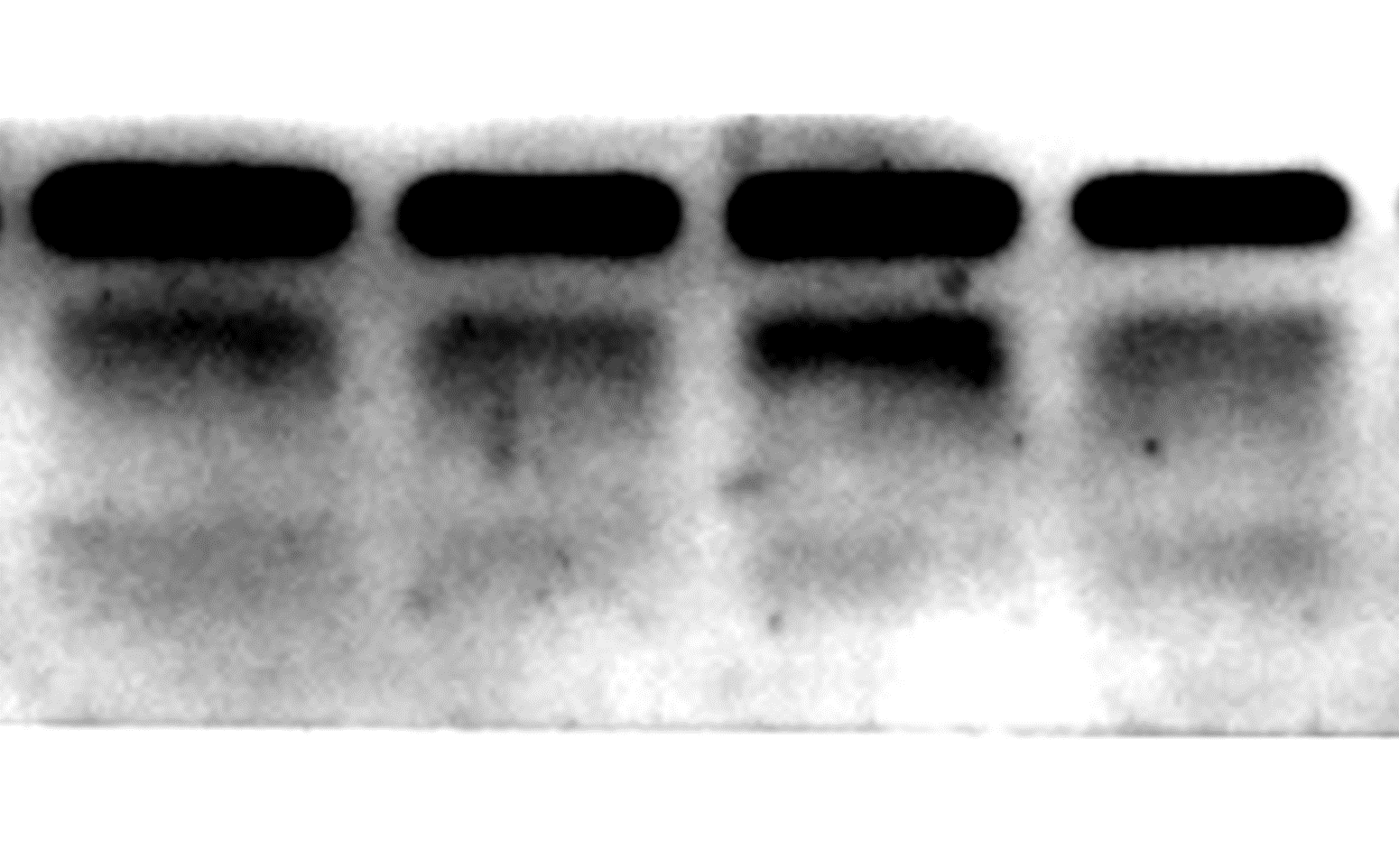

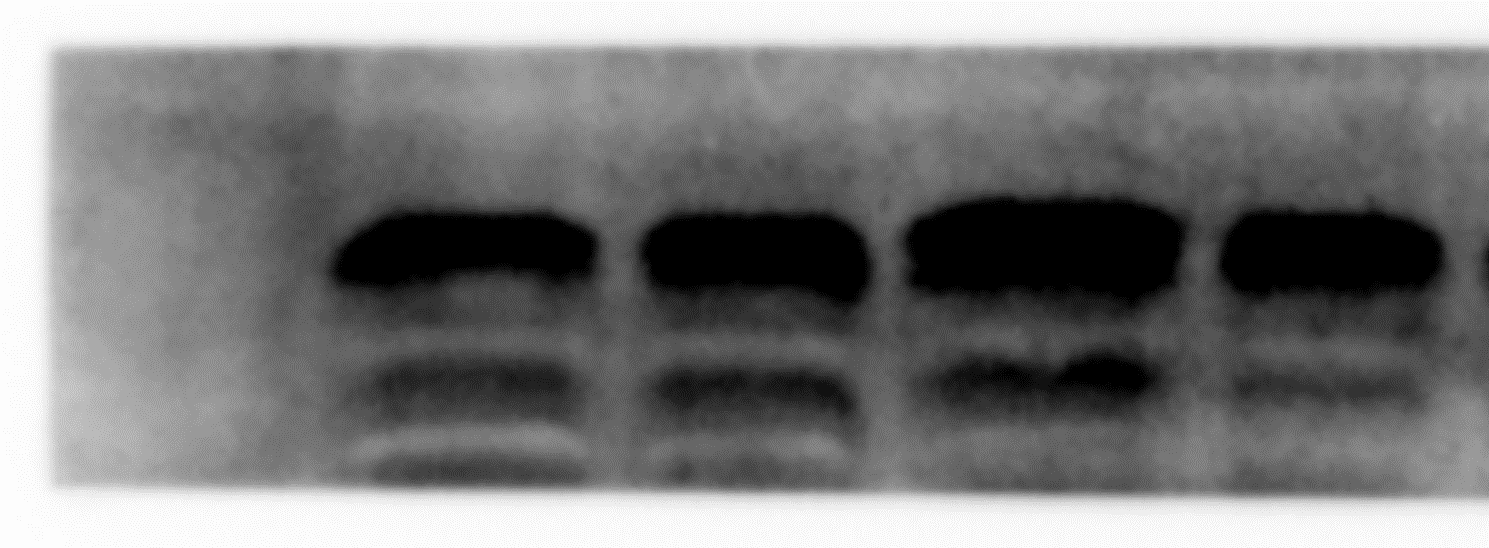

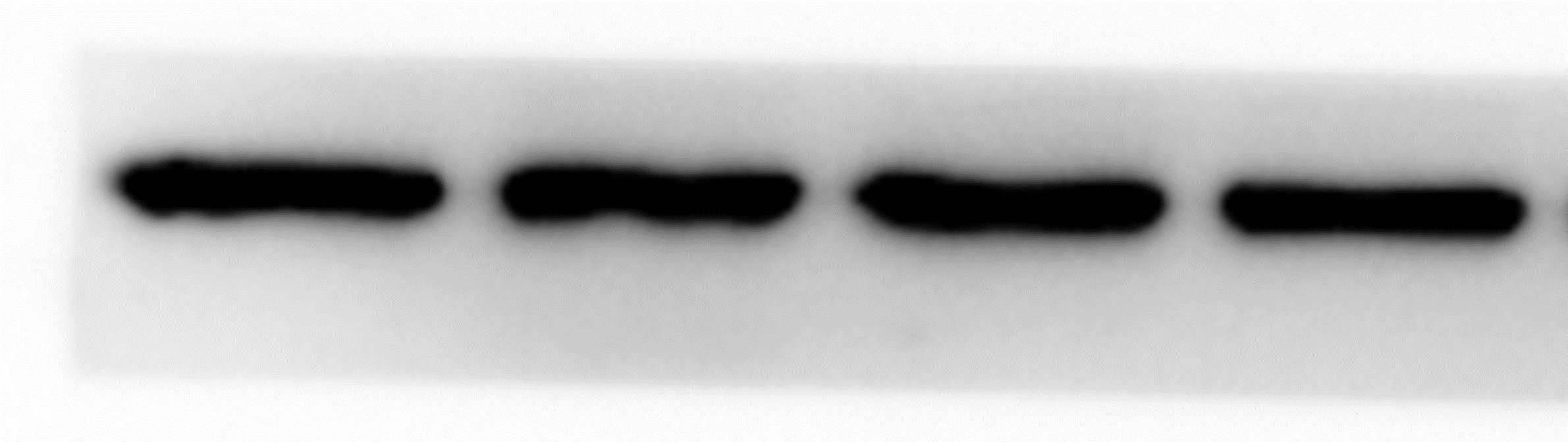

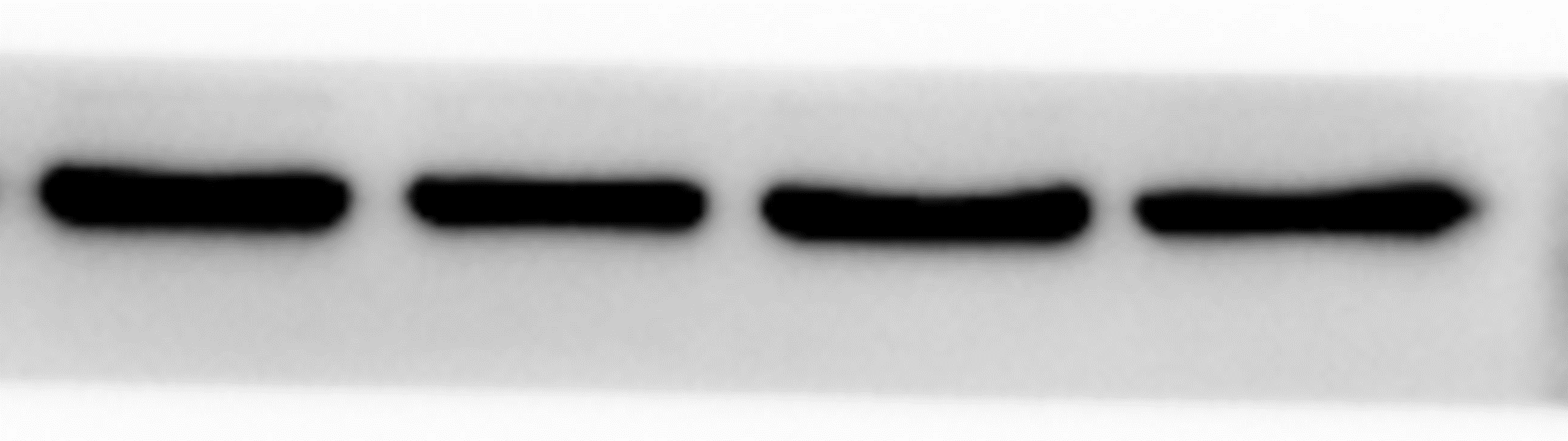

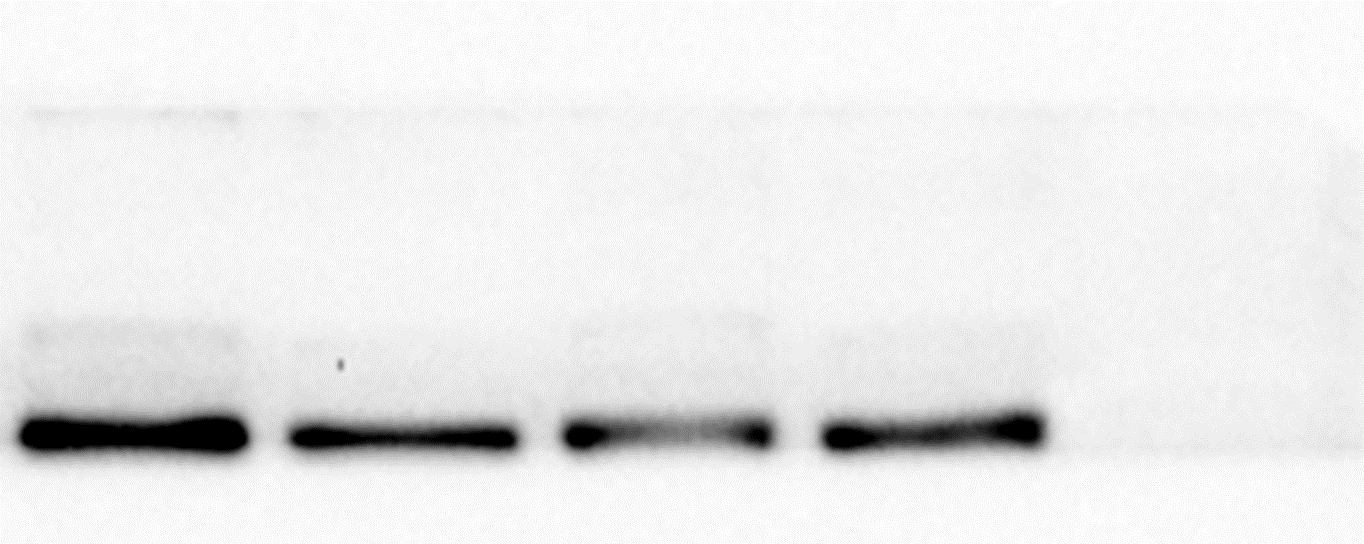


**0 100 0 100 (μM)**

**0 0 500 500 (μg/mL)**

**ACTB**

**p62**

**LC3-I**

**LC3-II**

**3-MA: 0 100 0 100 (μM)**

**PS-NPs: 0 0 500 500 (μg/mL)**

**0 100 0 100 (μM)**

**0 0 500 500 (μg/mL)**

**Figure S5A**

**PS-NP concentration (μg/mL)**

**0**

**0.5**

**5**

**50**

**500**

**PS-NP concentration (μg/mL)**

**0**

**0.5**

**5**

**50**

**500**

**PS-NP concentration (μg/mL)**

**0**

**0.5**

**5**

**50**

**500**

**pAMPK**


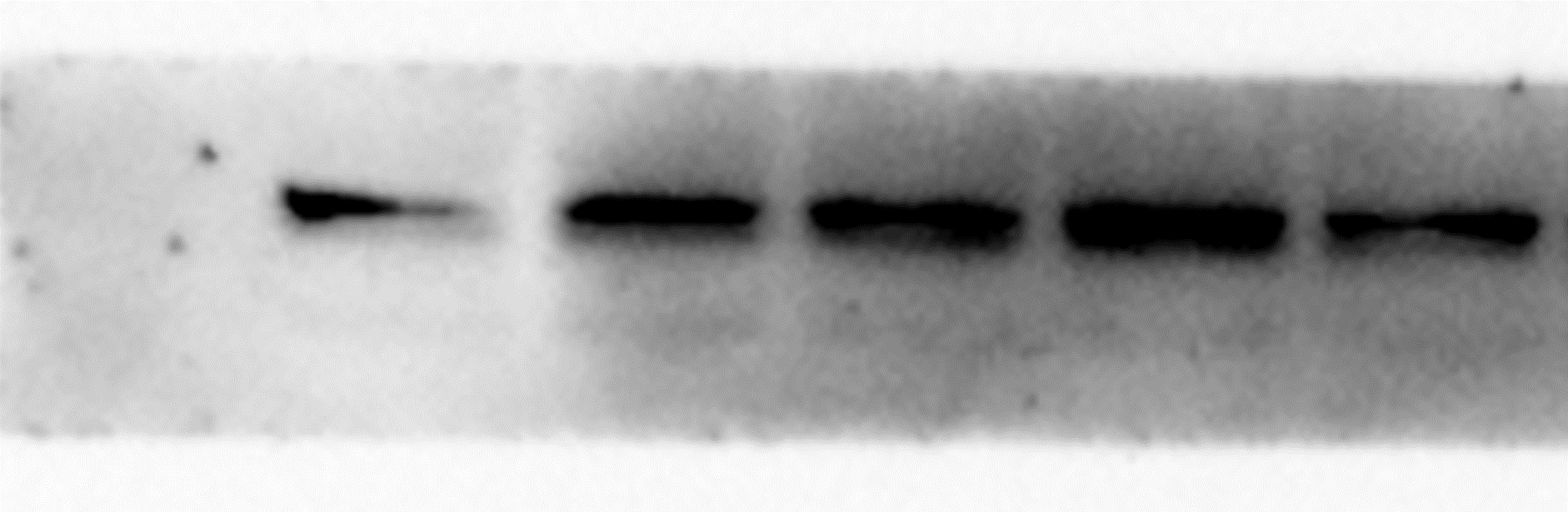

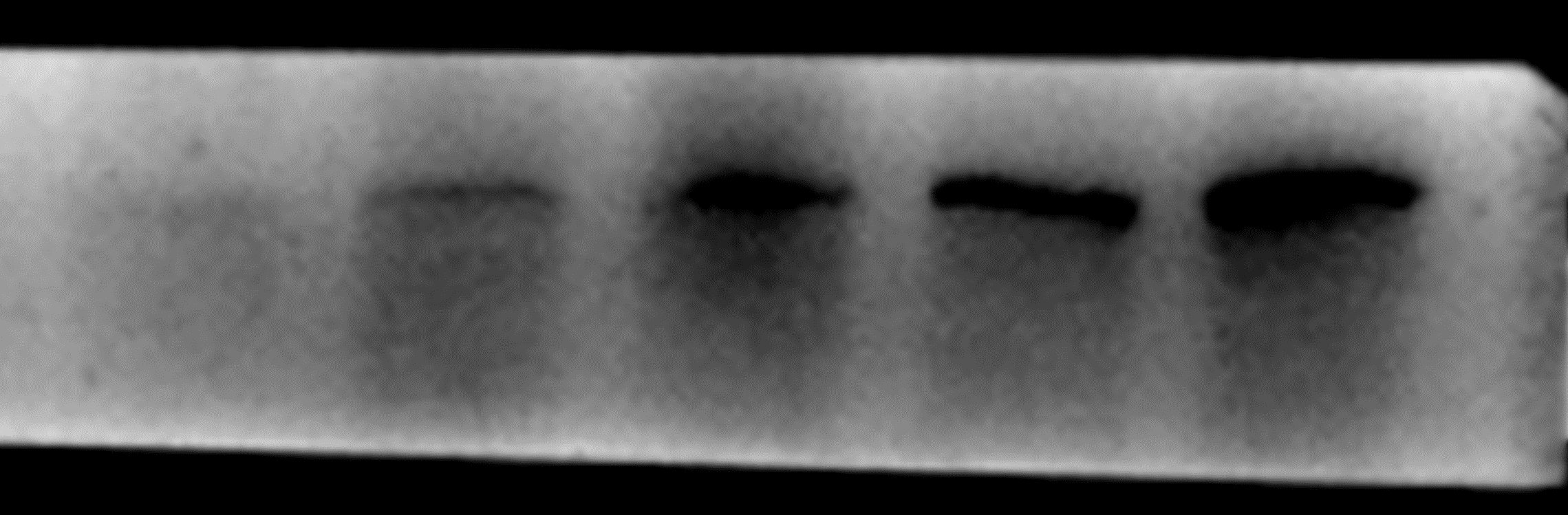

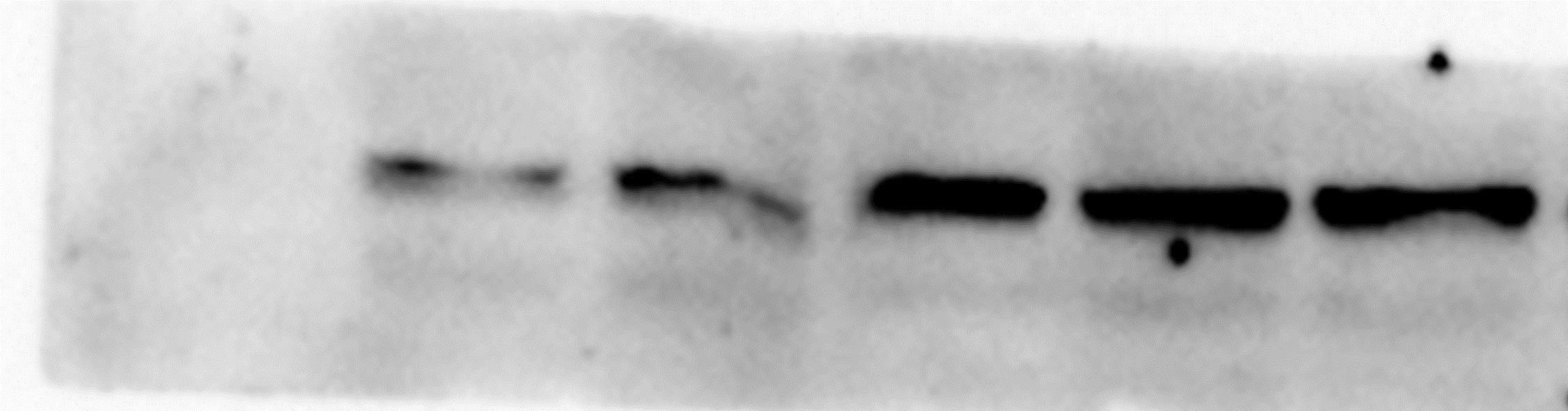


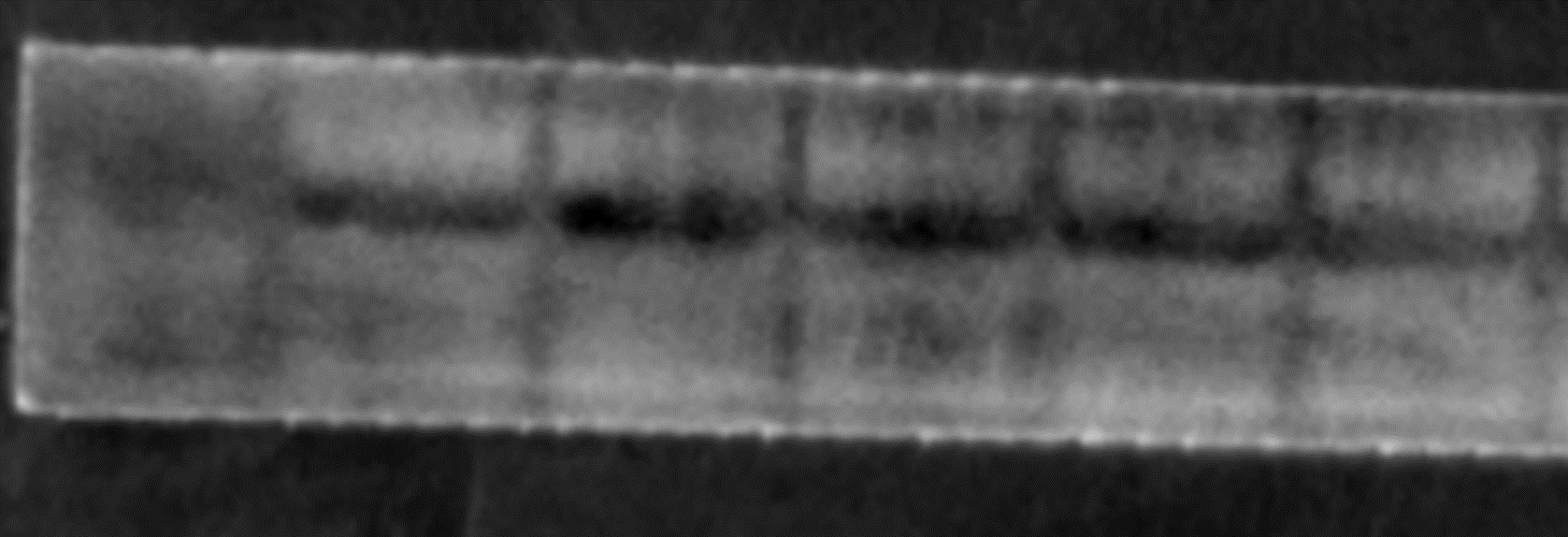

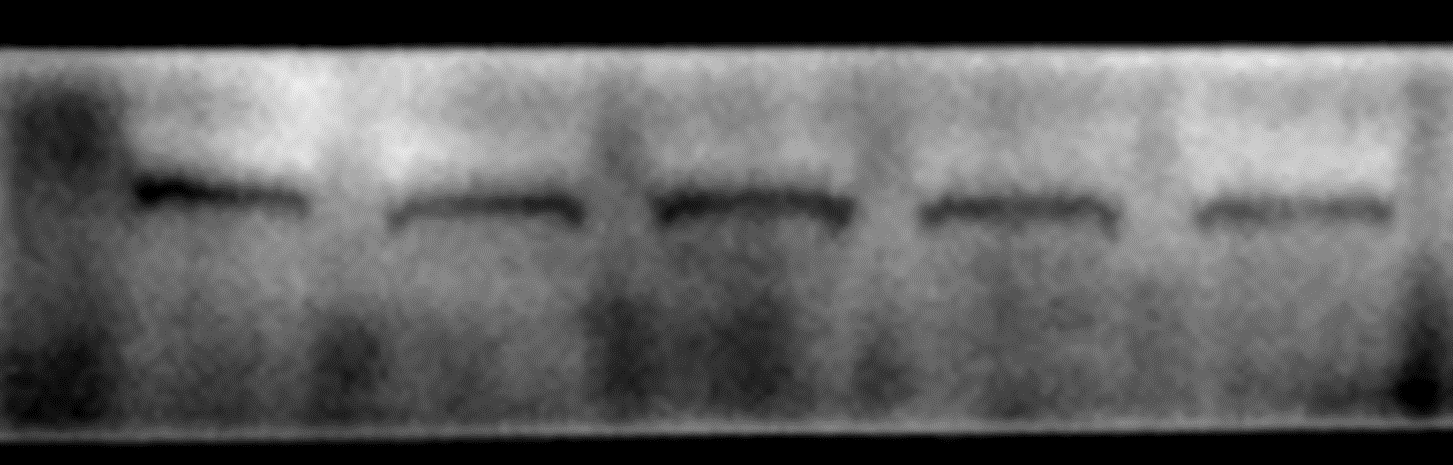

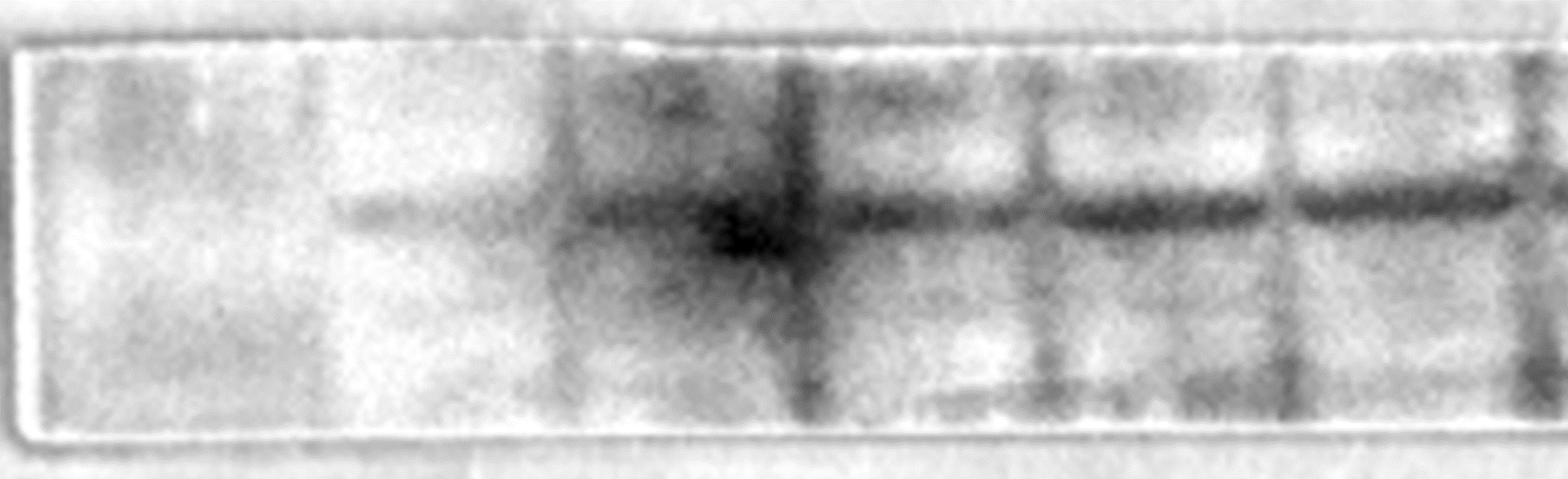


**AMPK**


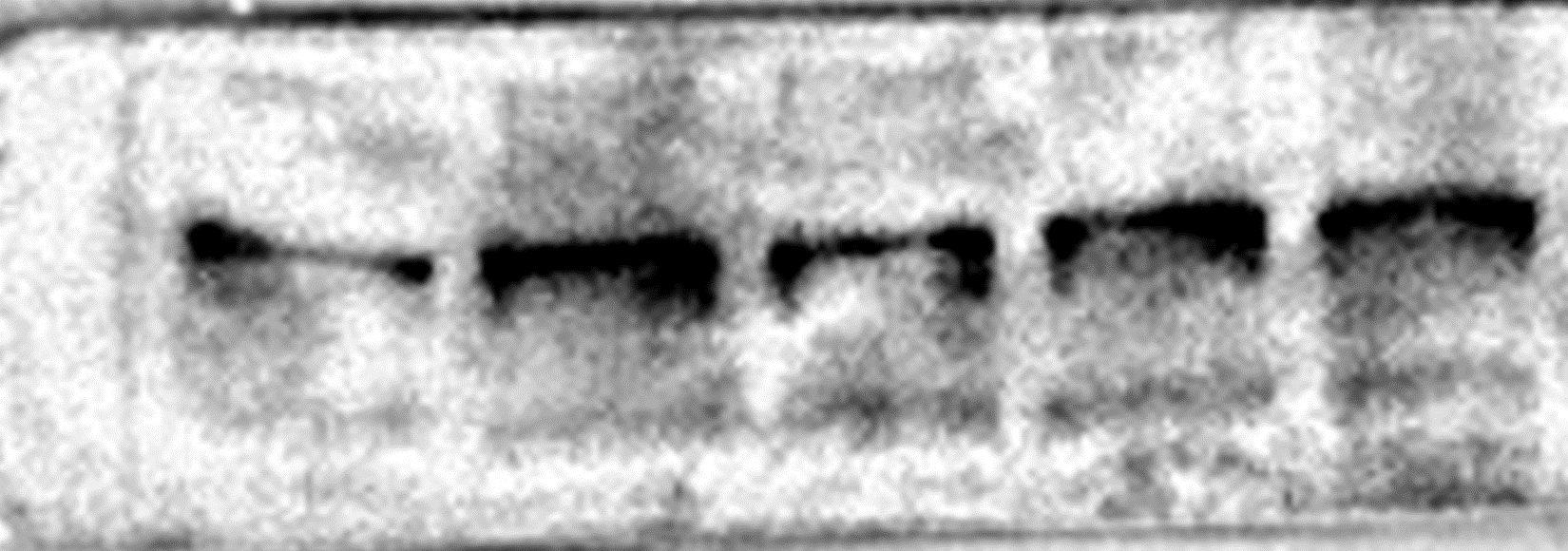

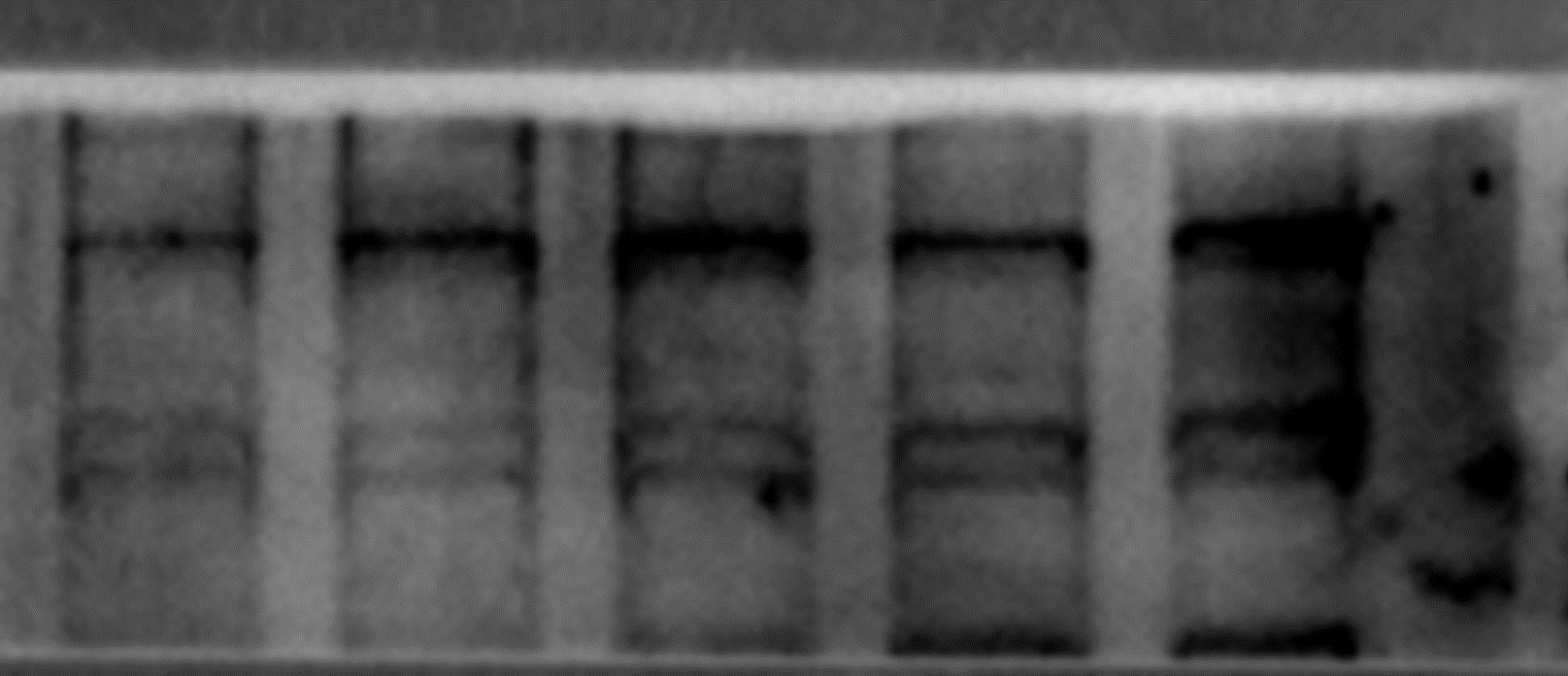

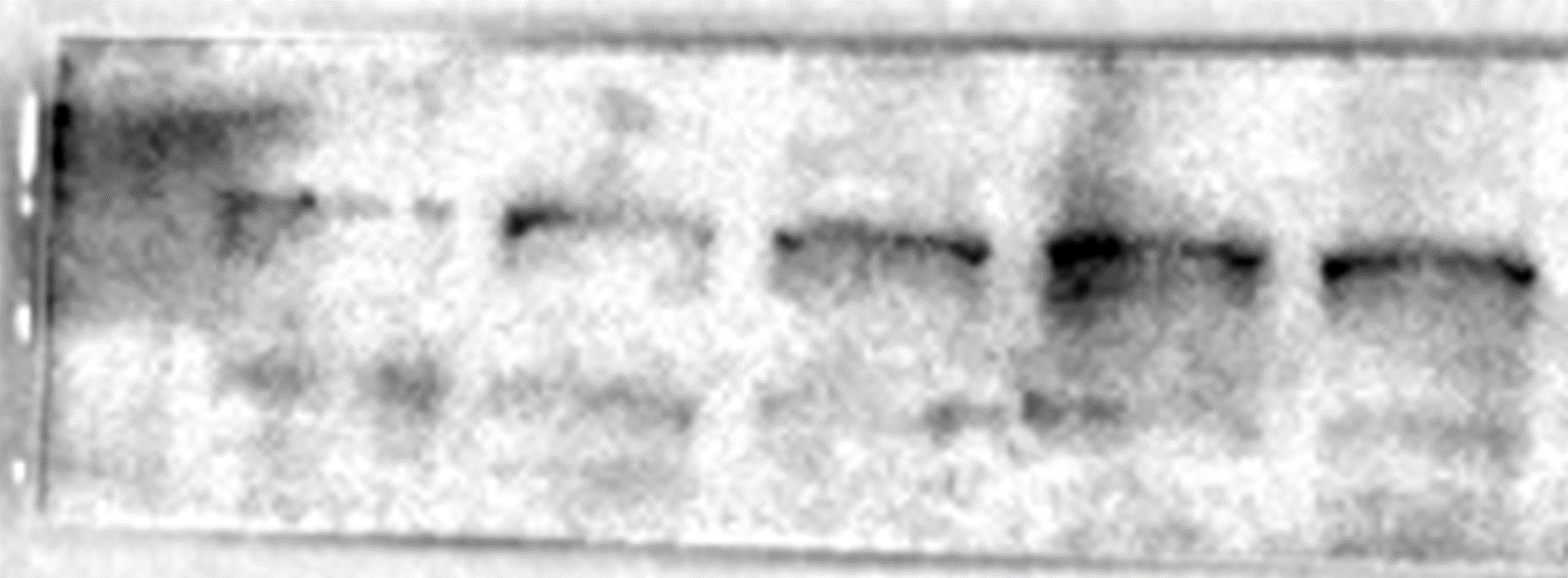


**pULK1**


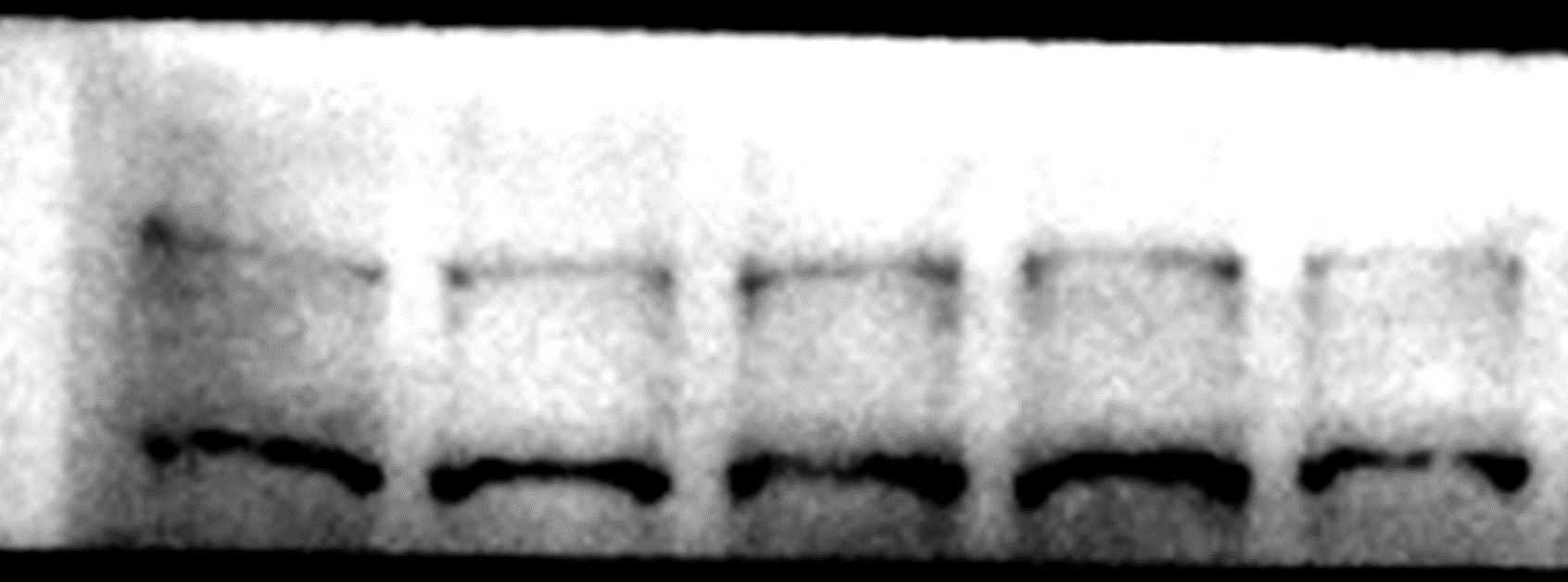

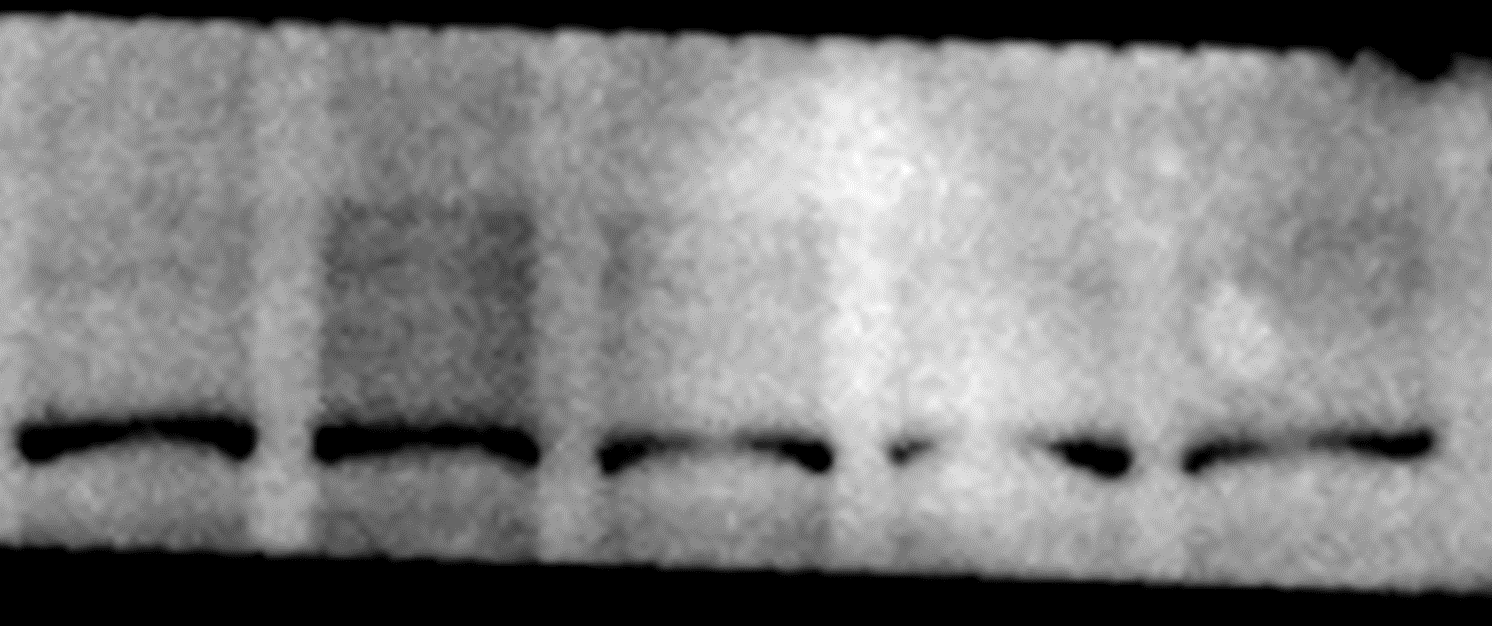

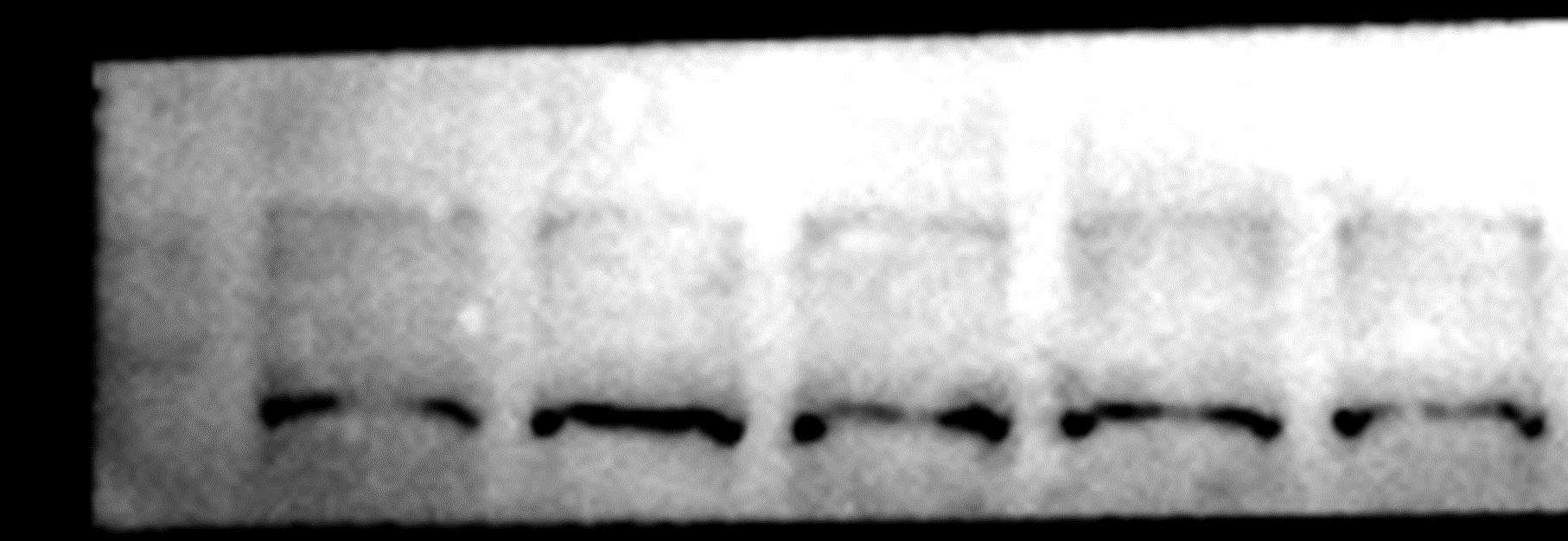


**ULK1**


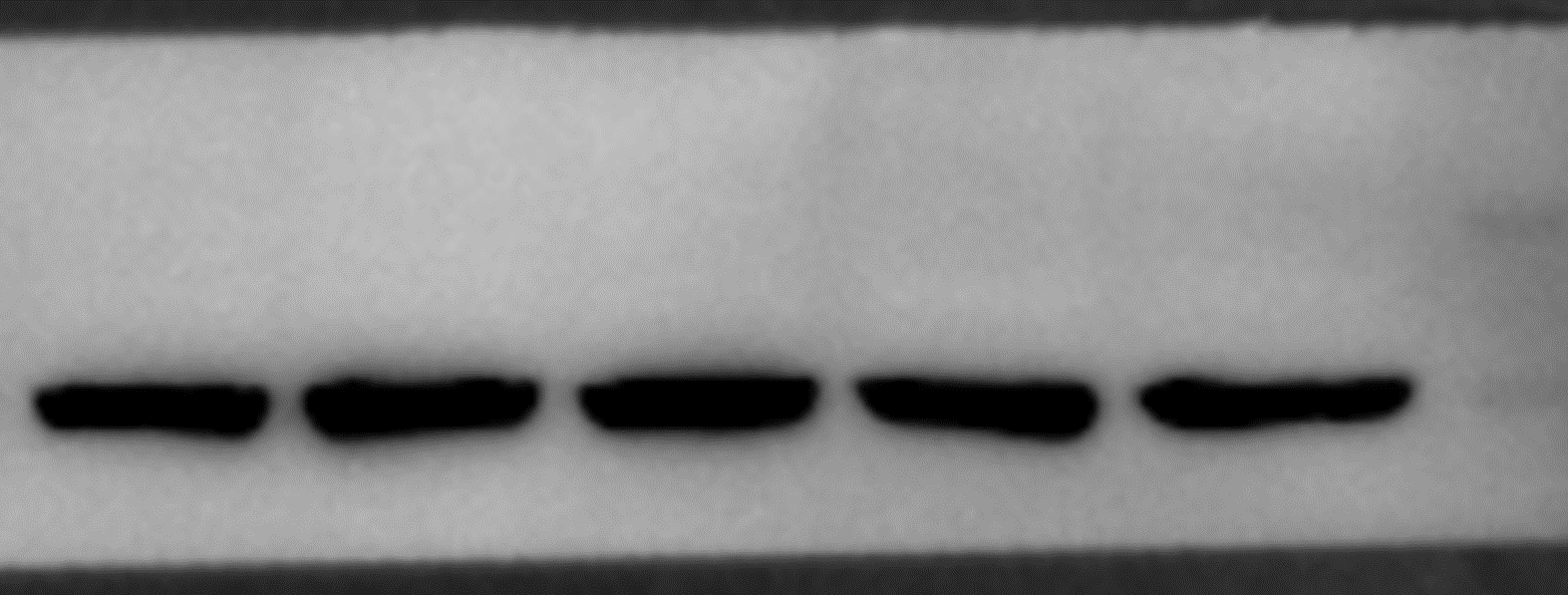

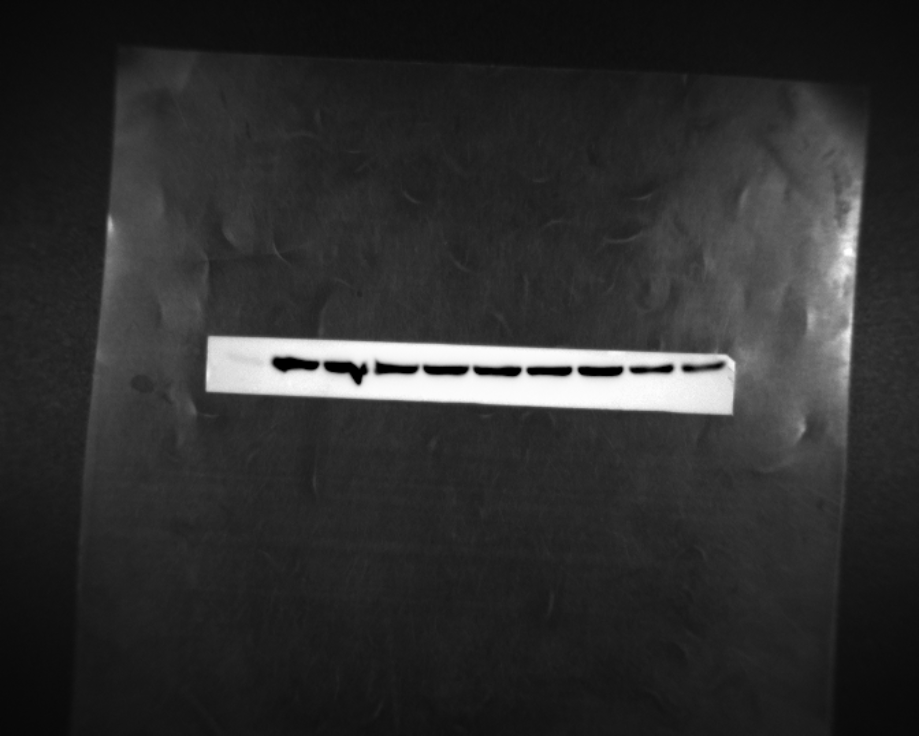


**ACTB**


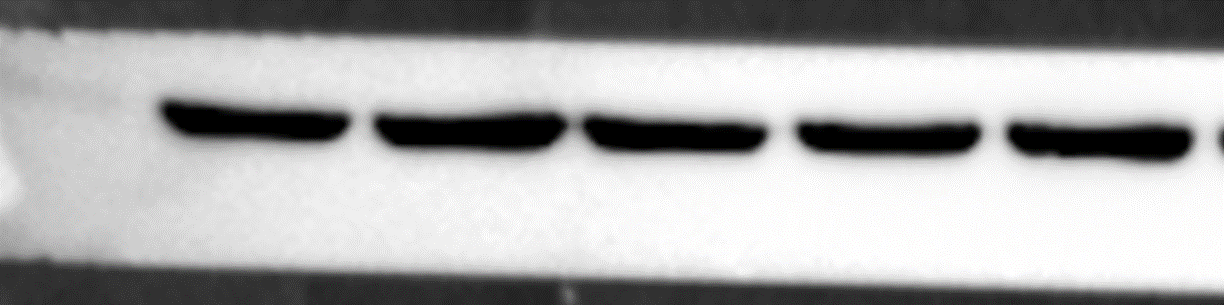


**Figure S5B**

**0 0.5 0 0.5 (μM)**

**0 0 500 500 (μg/mL)**

**0 0.5 0 0.5 (μM)**

**0 0 500 500 (μg/mL)**

**CoC: 0 0.5 0 0.5 (μM)**

**PS-NPs: 0 0 500 500 (μg/mL)**


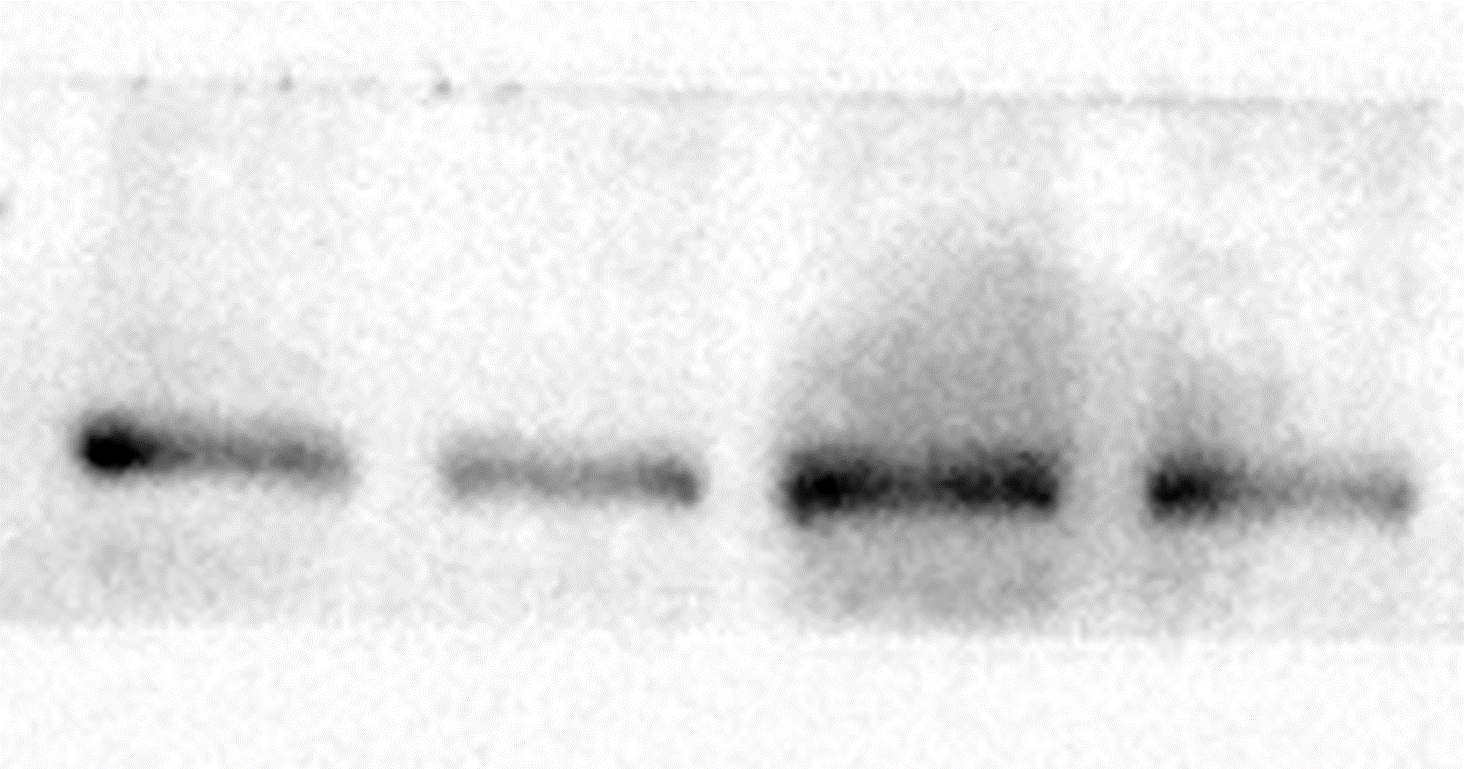


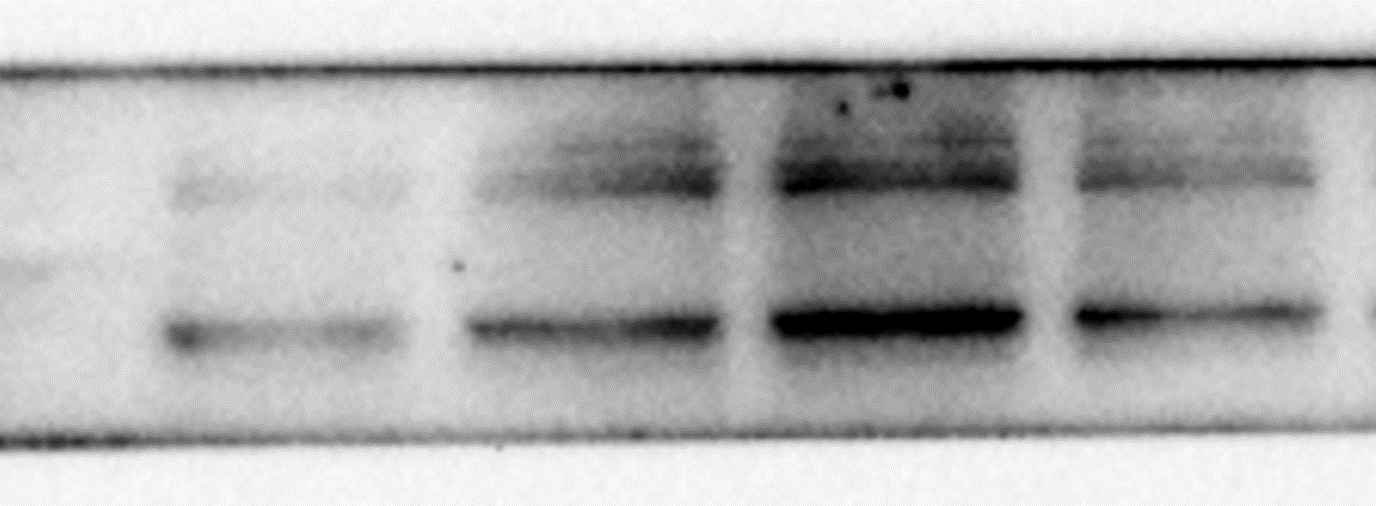

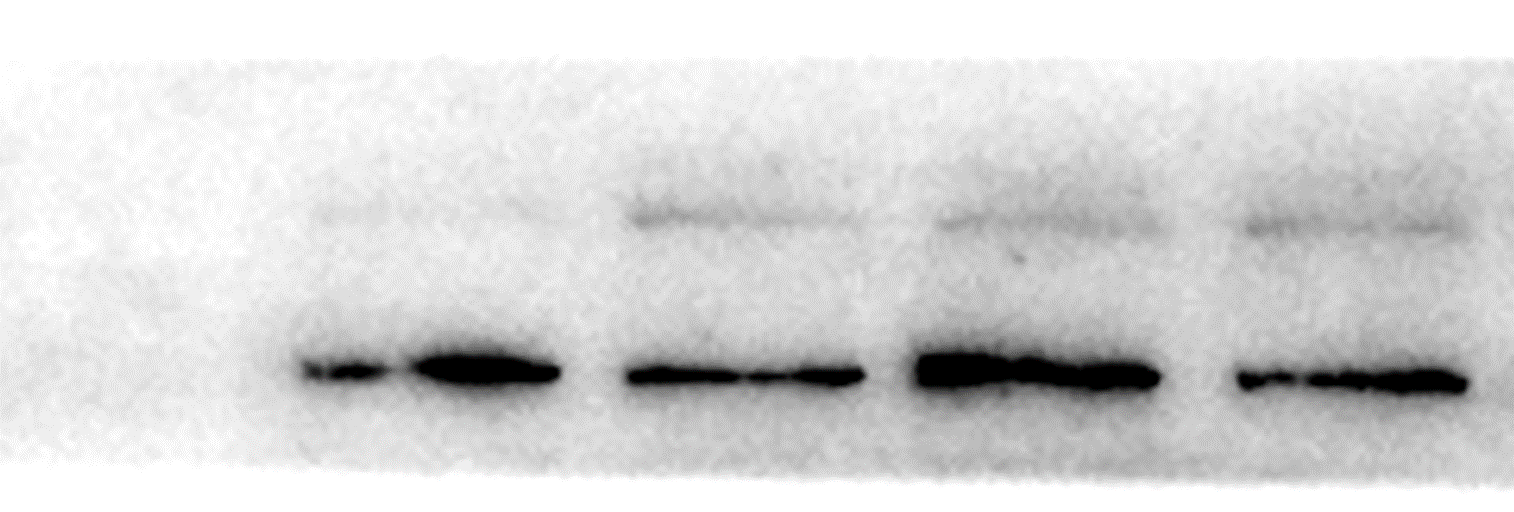


**pAMPK**


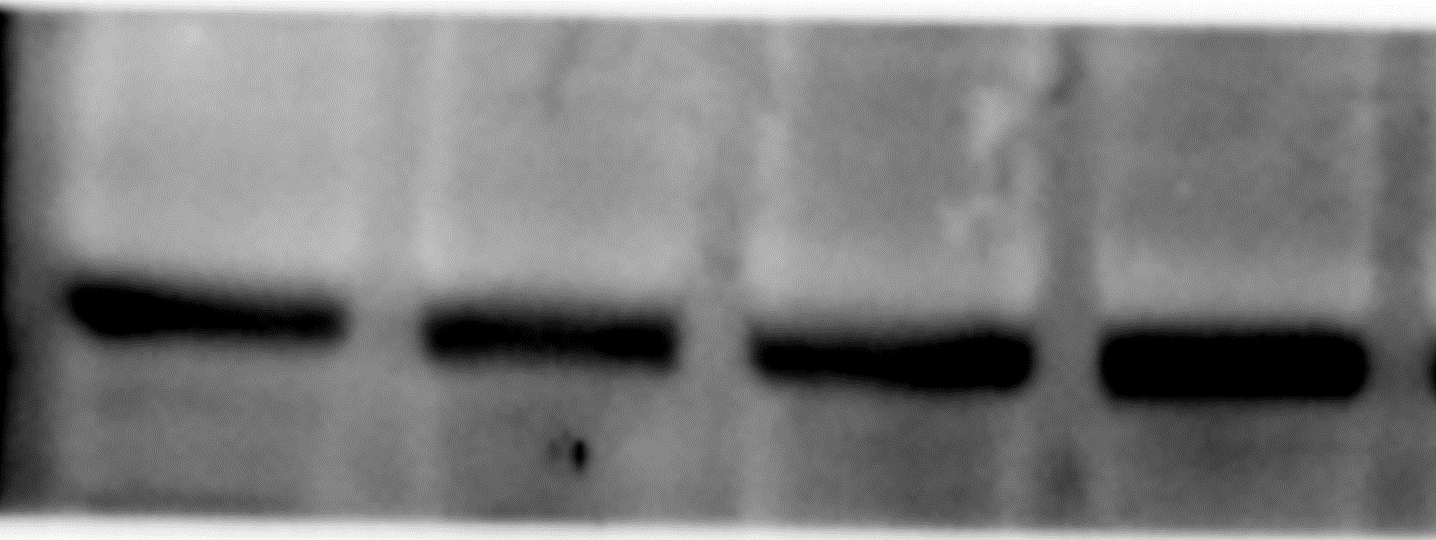

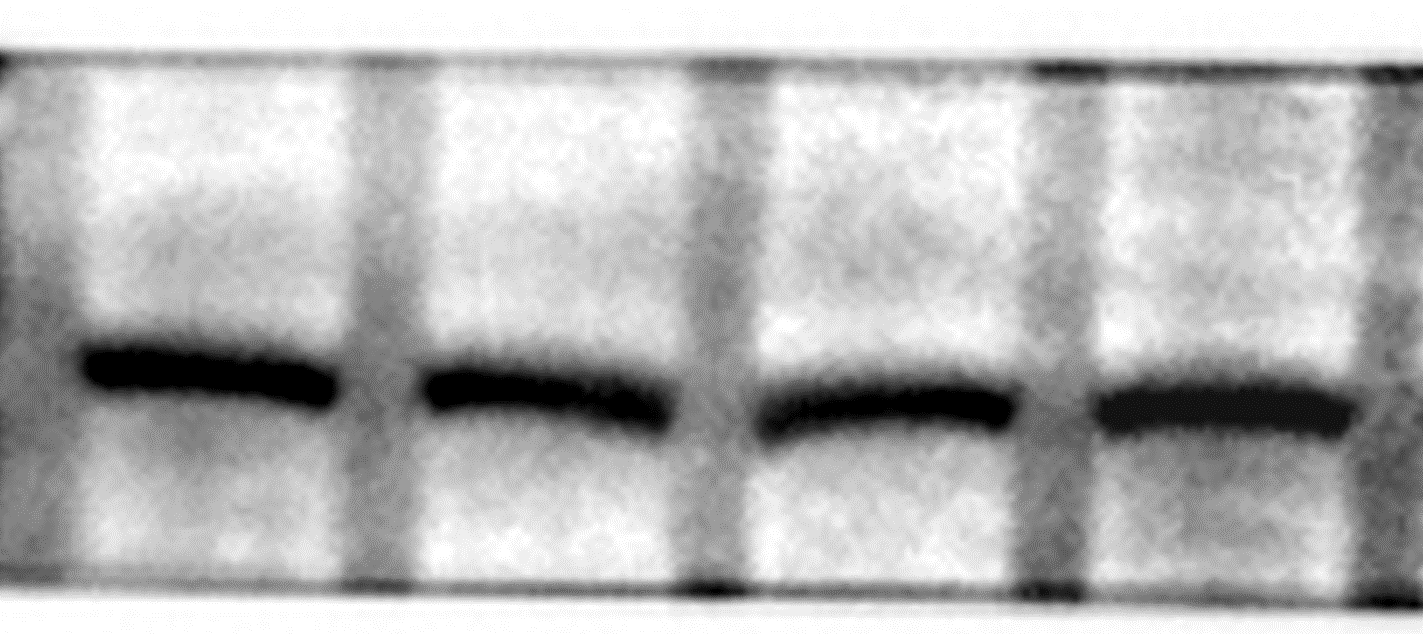

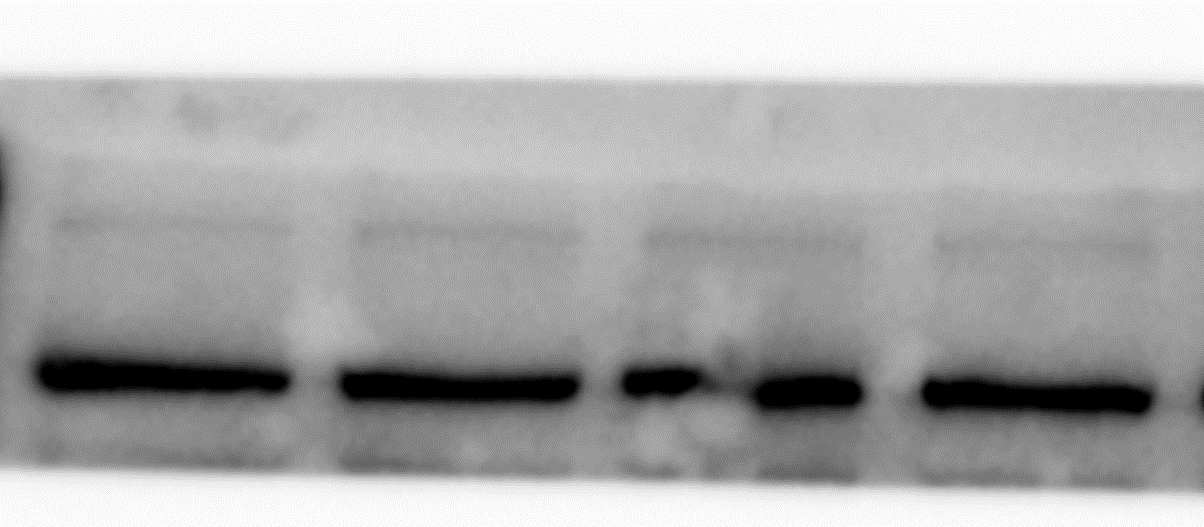


**AMPK**


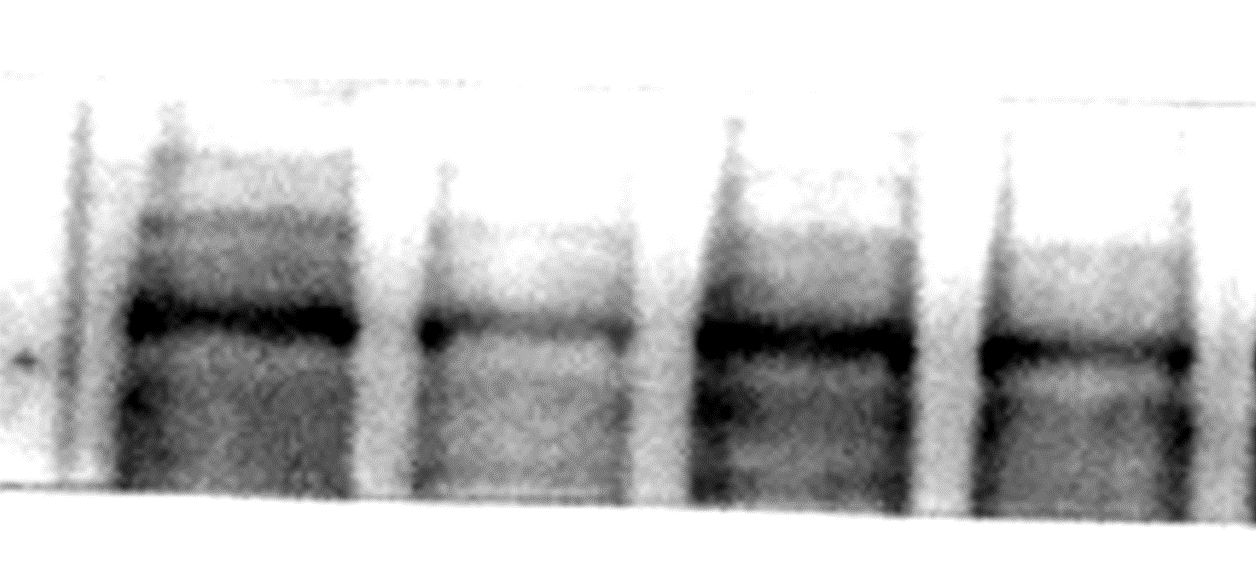


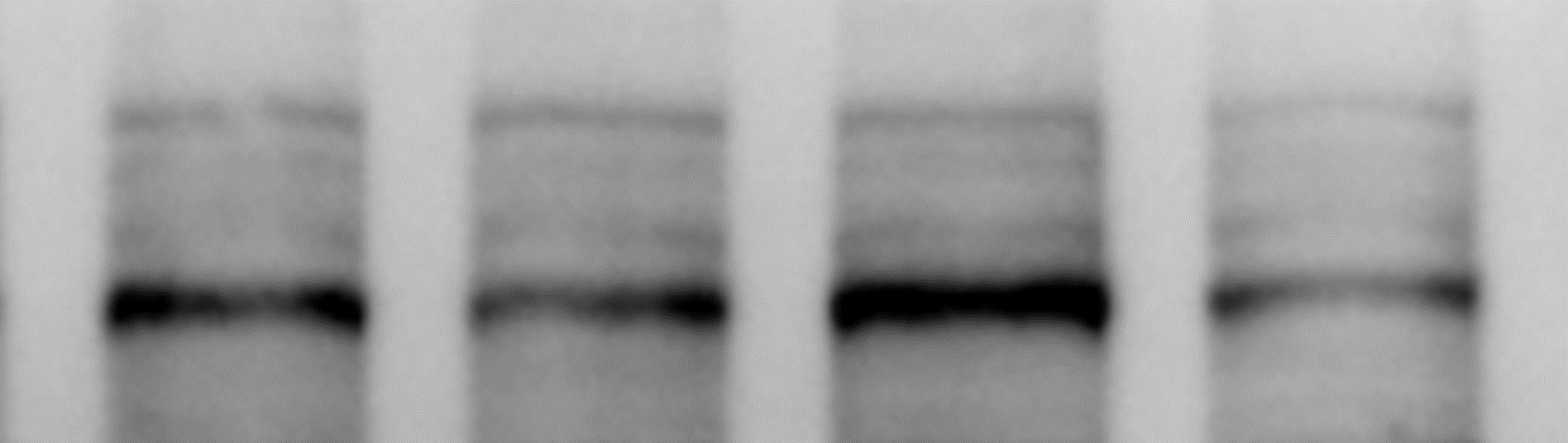

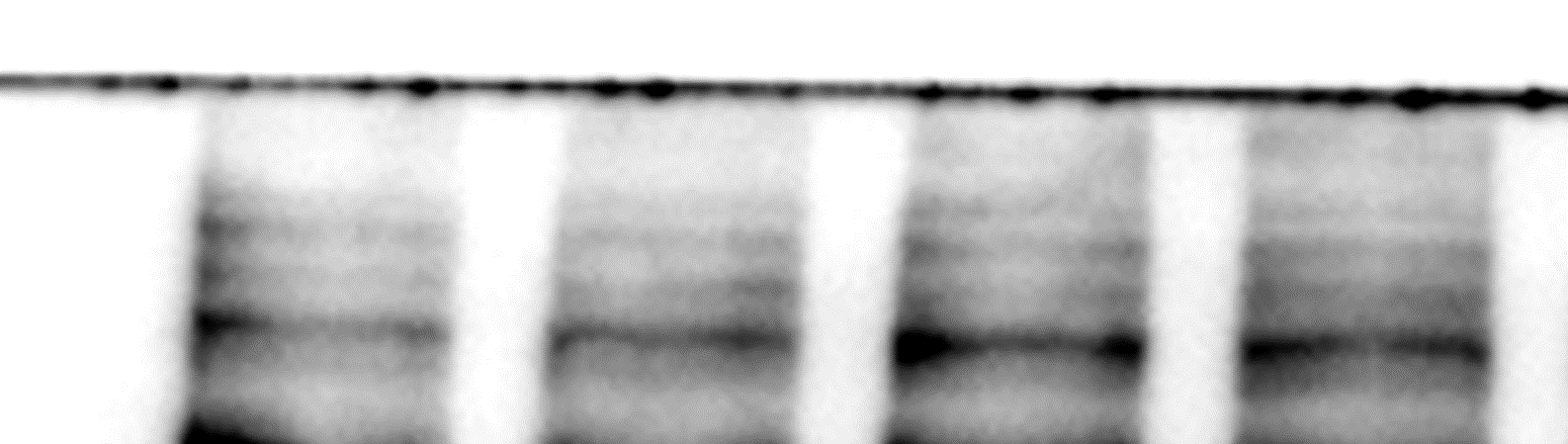


**pULK1**


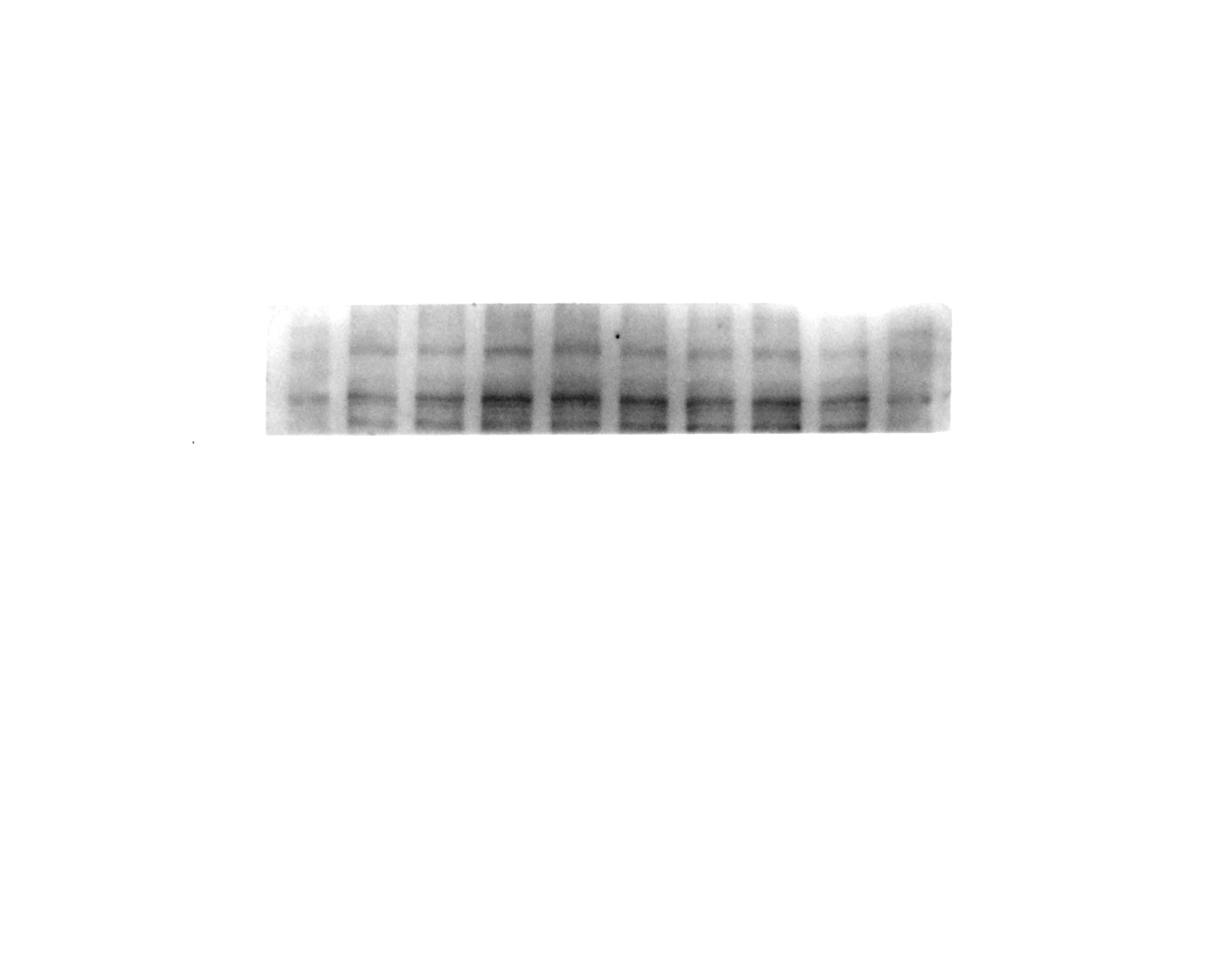

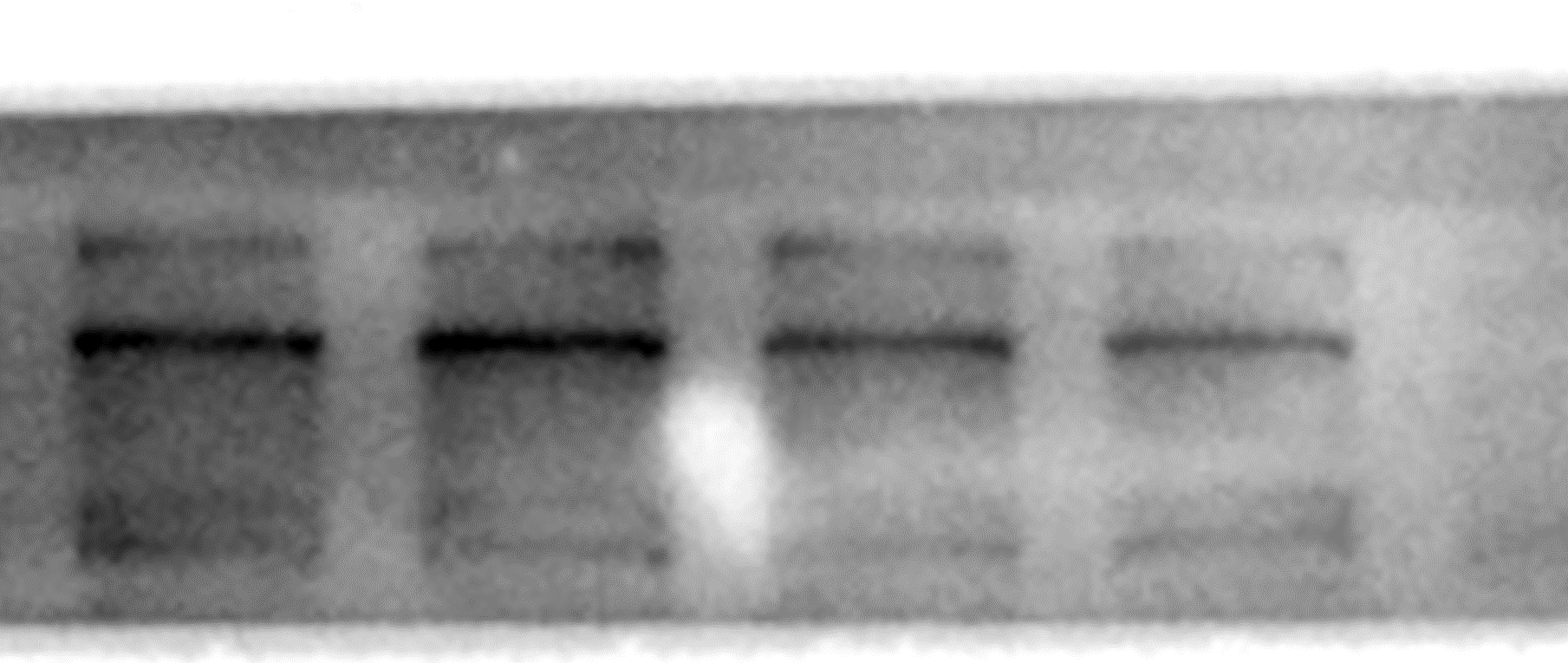


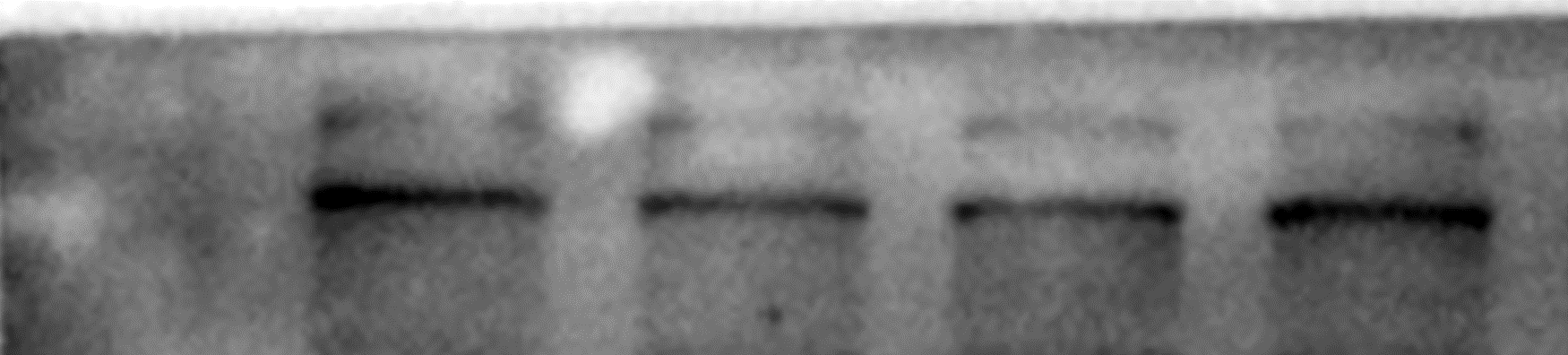


**ULK1**


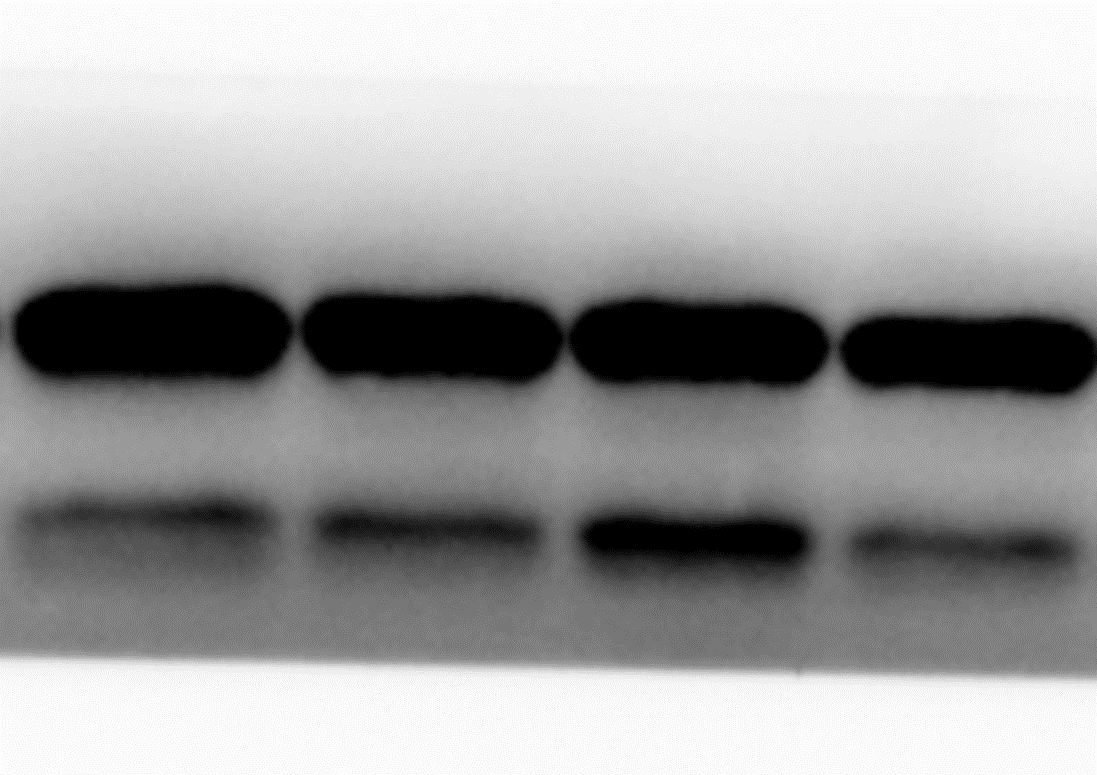


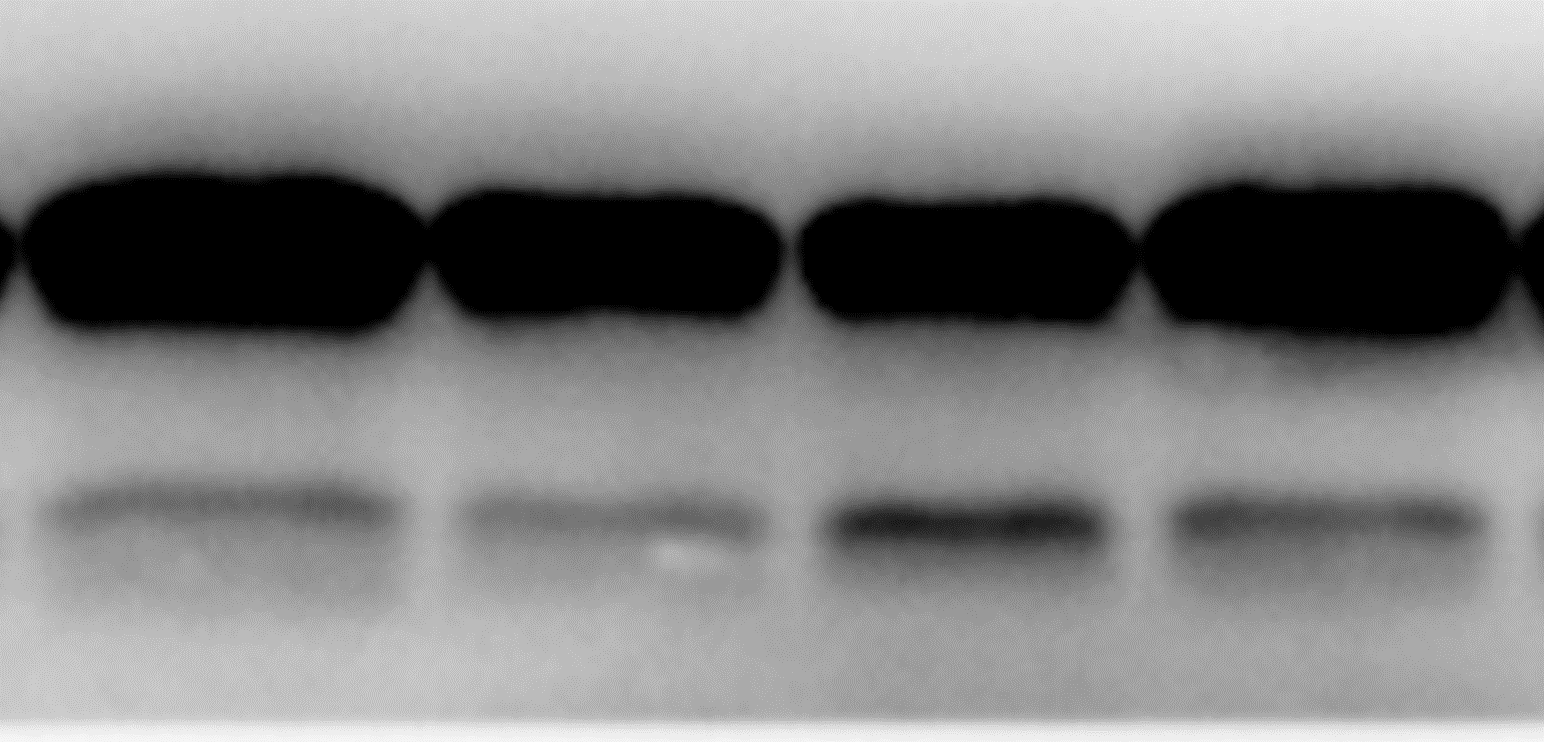

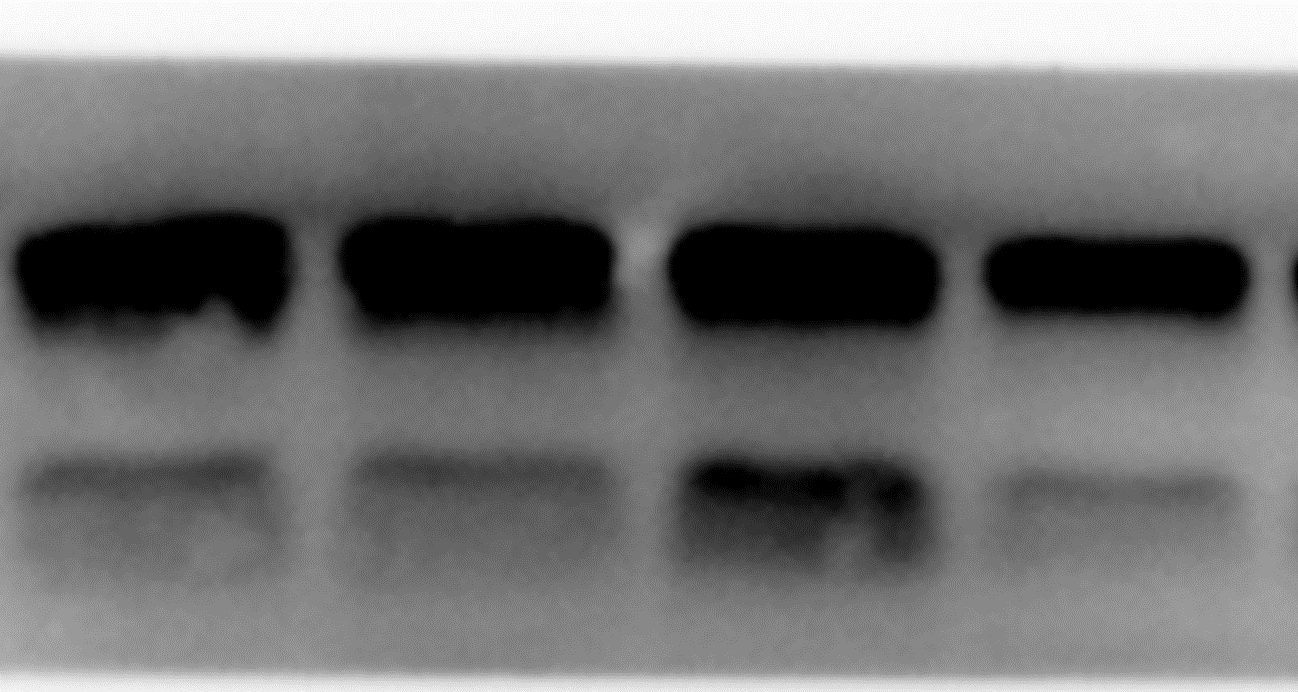


**LC3-I**

**LC3-II**


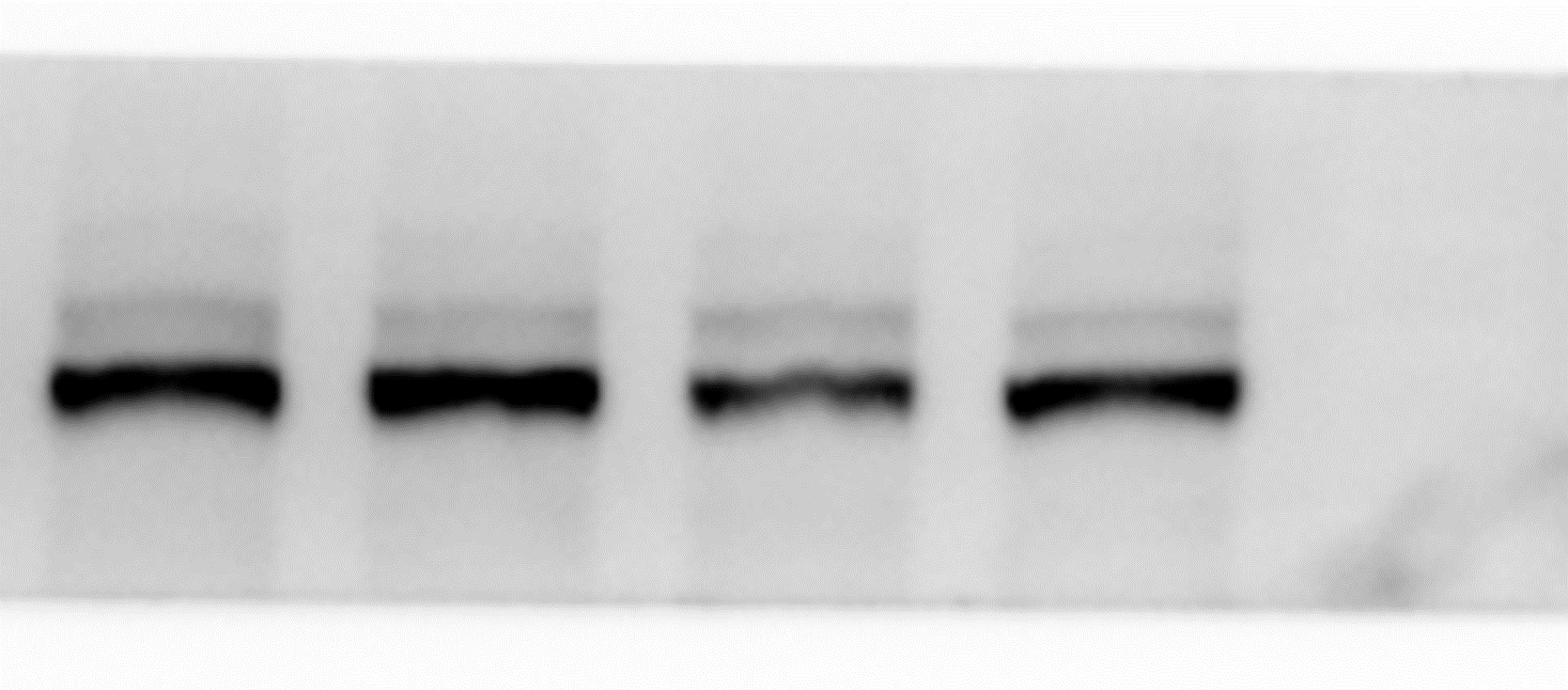

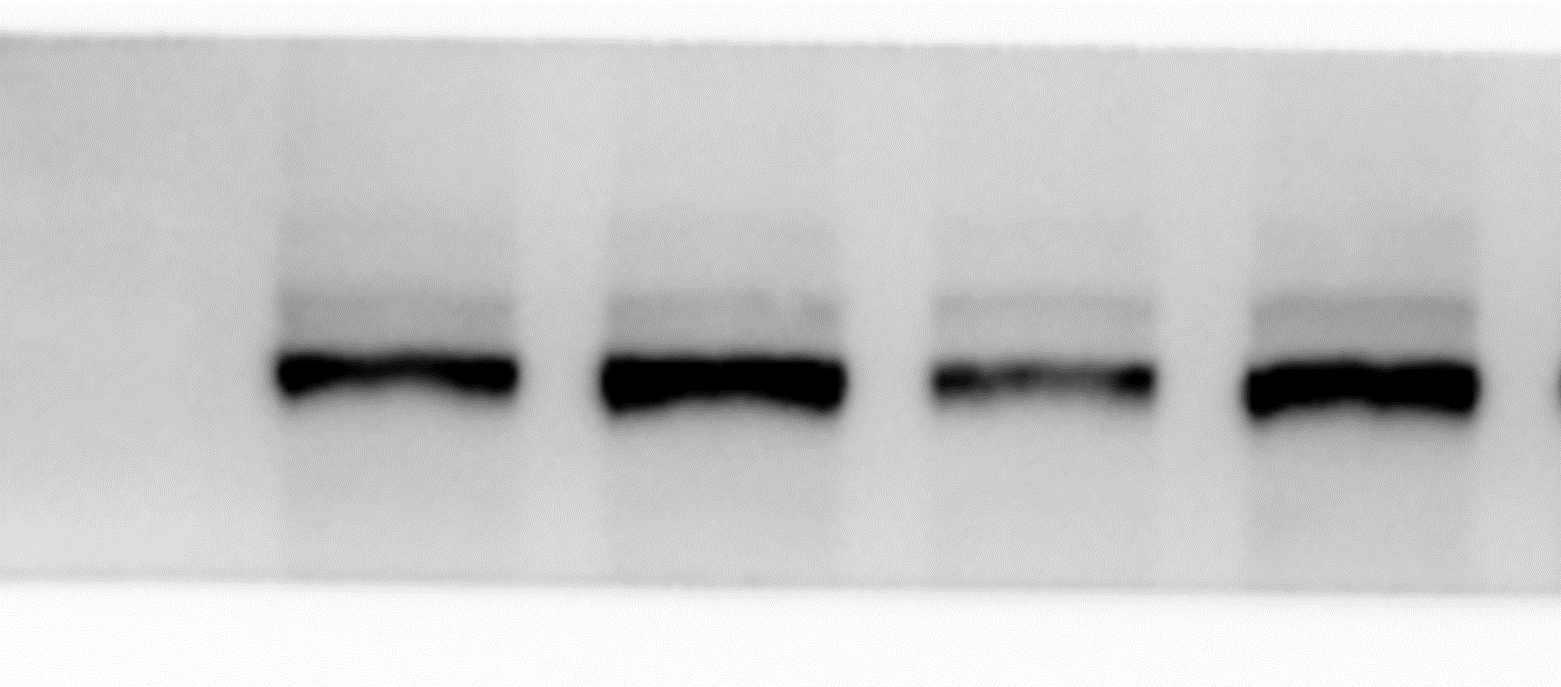


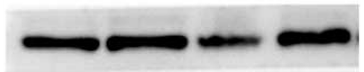


**p62**


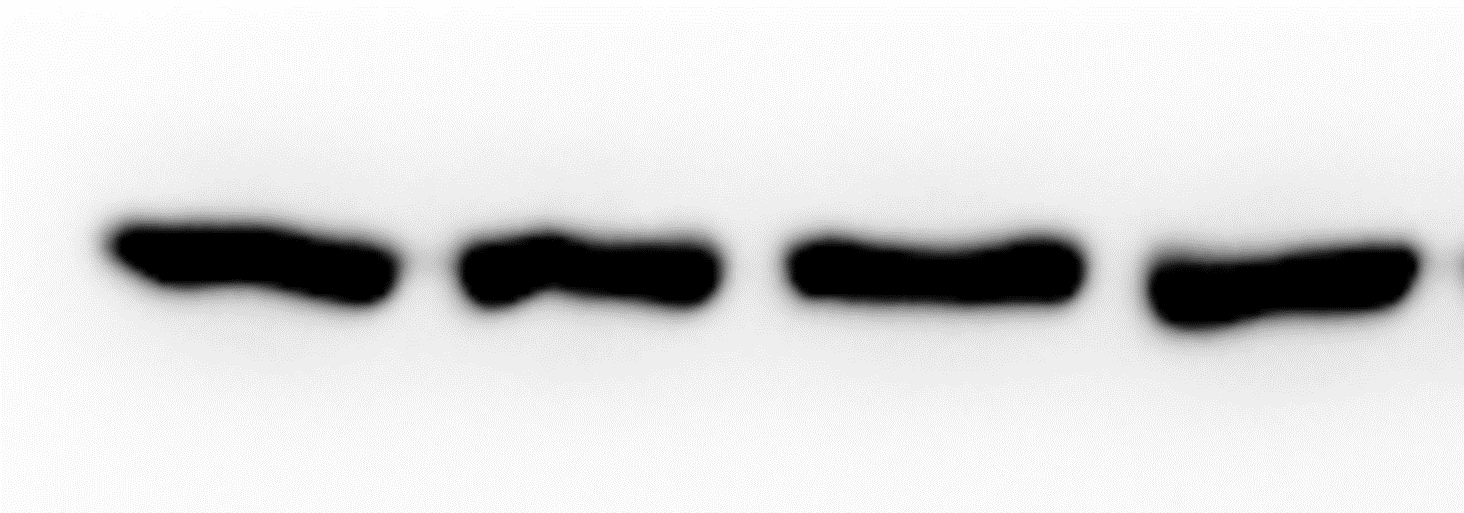


**ACTB**


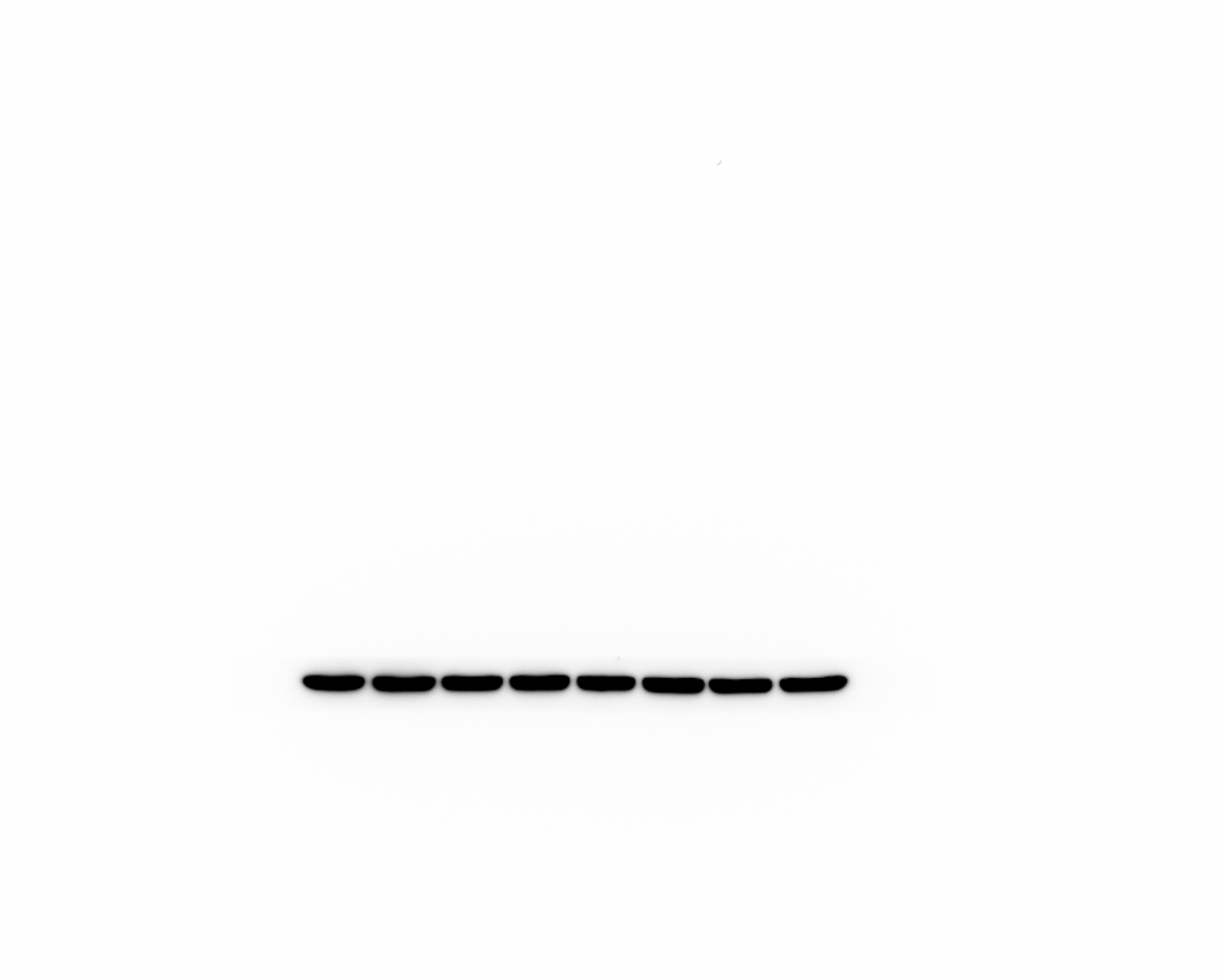

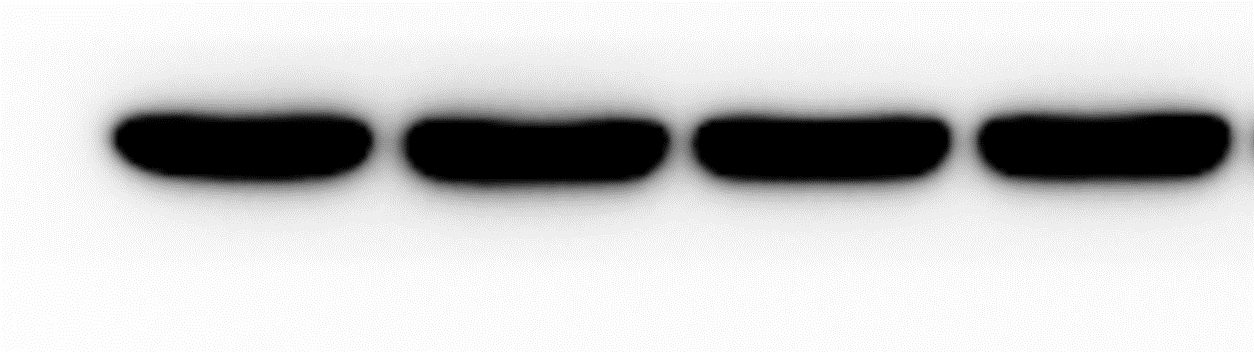


**Figure S6**

**0 16 0 16 (μM)**

**0 0 500 500 (μg/mL)**

**0 16 0 16 (μM)**

**0 0 500 500 (μg/mL)**

**Mel: 0 16 0 16 (μM)**

**PS-NPs: 0 0 500 500 (μg/mL)**


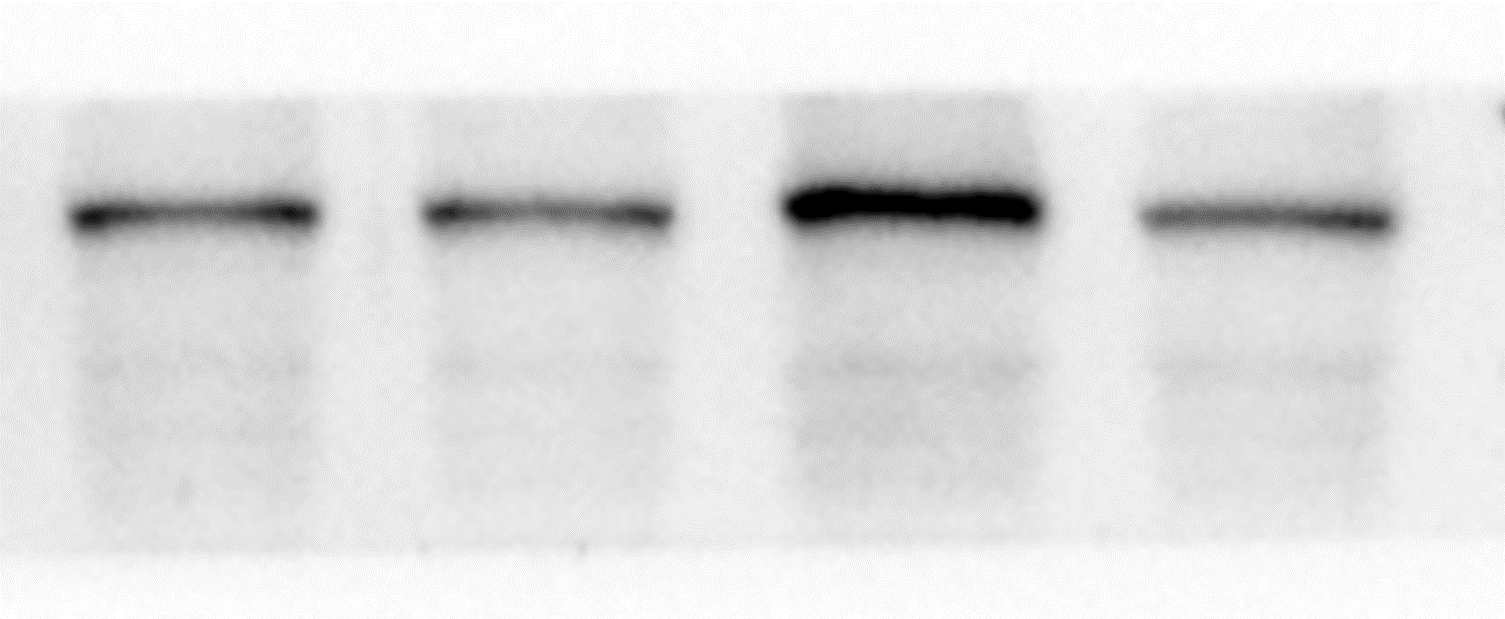

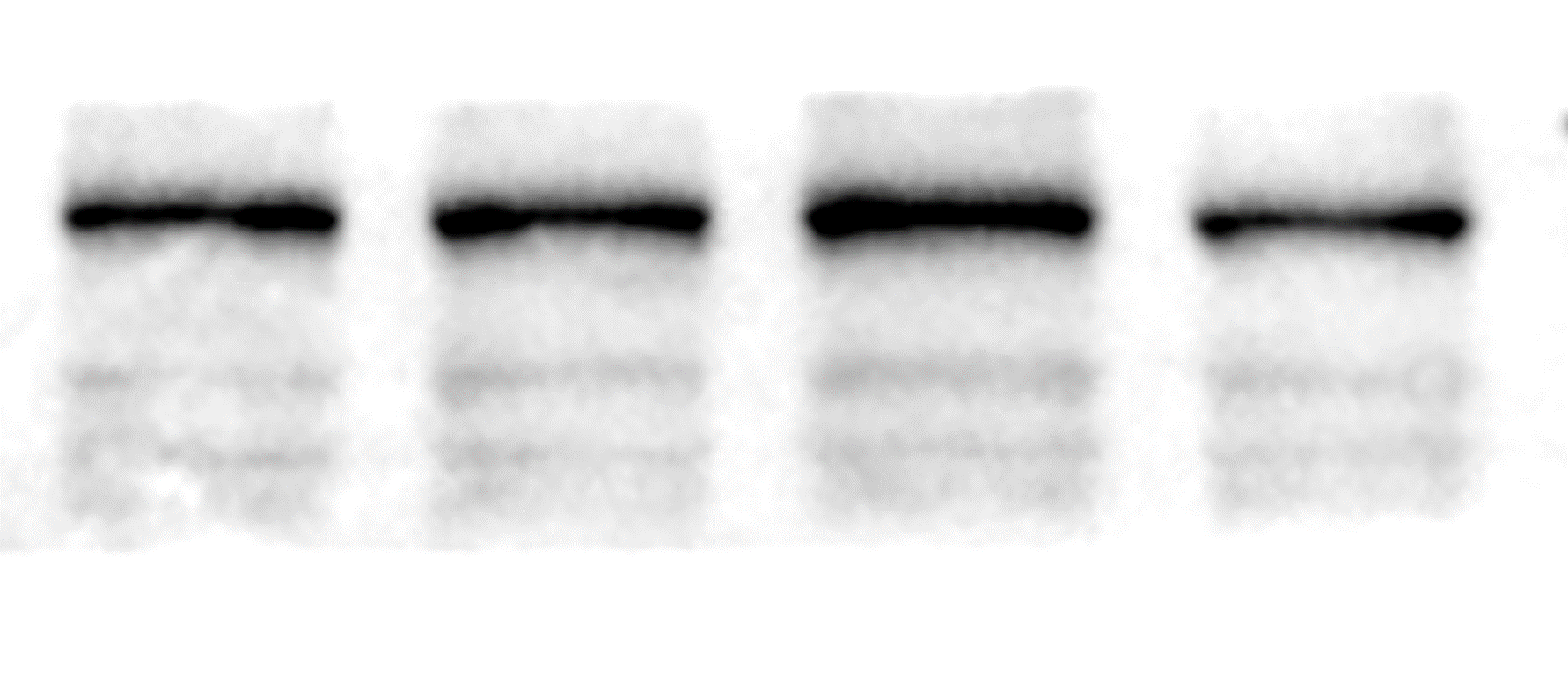

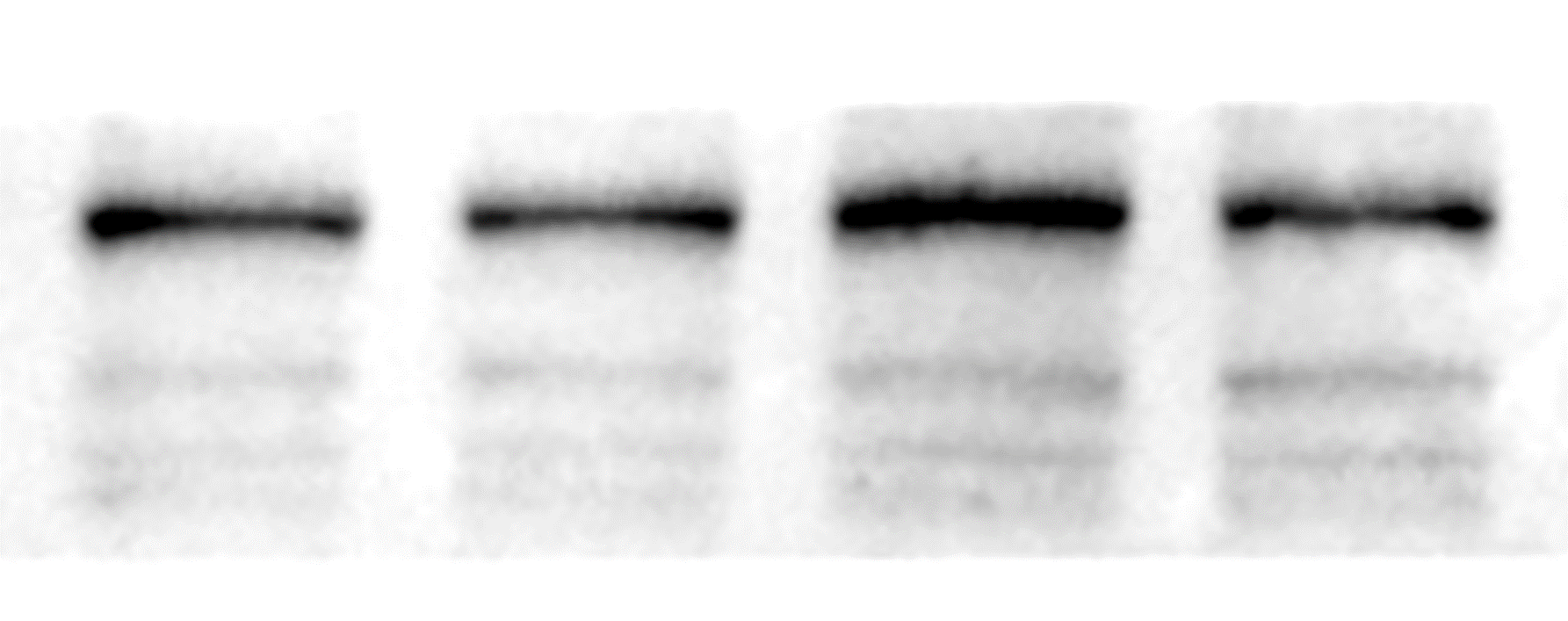


**pAMPK**


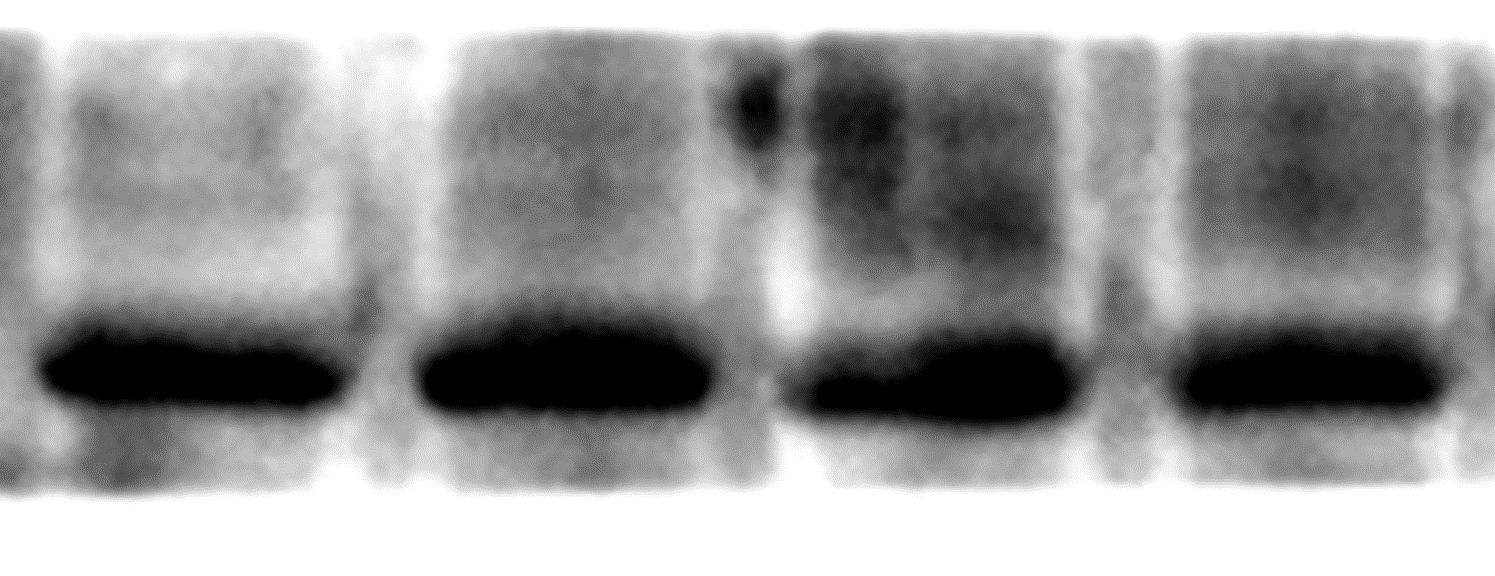

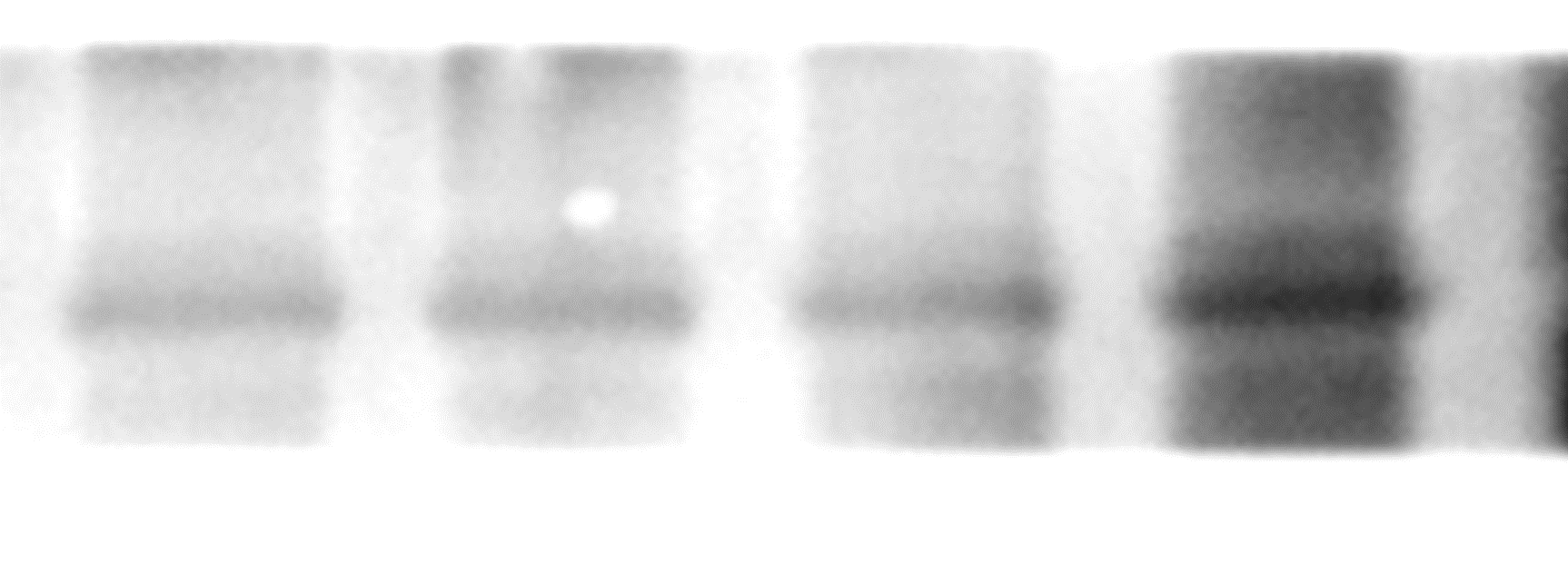

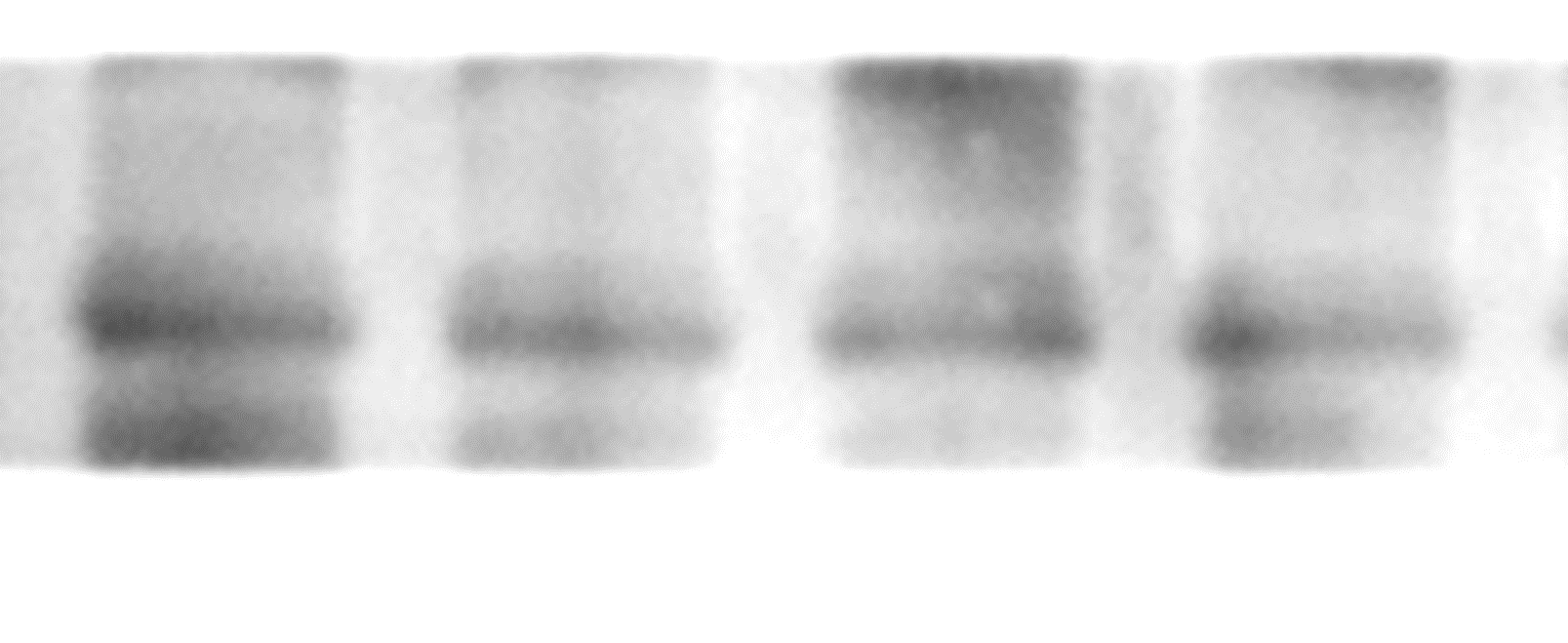


**AMPK**


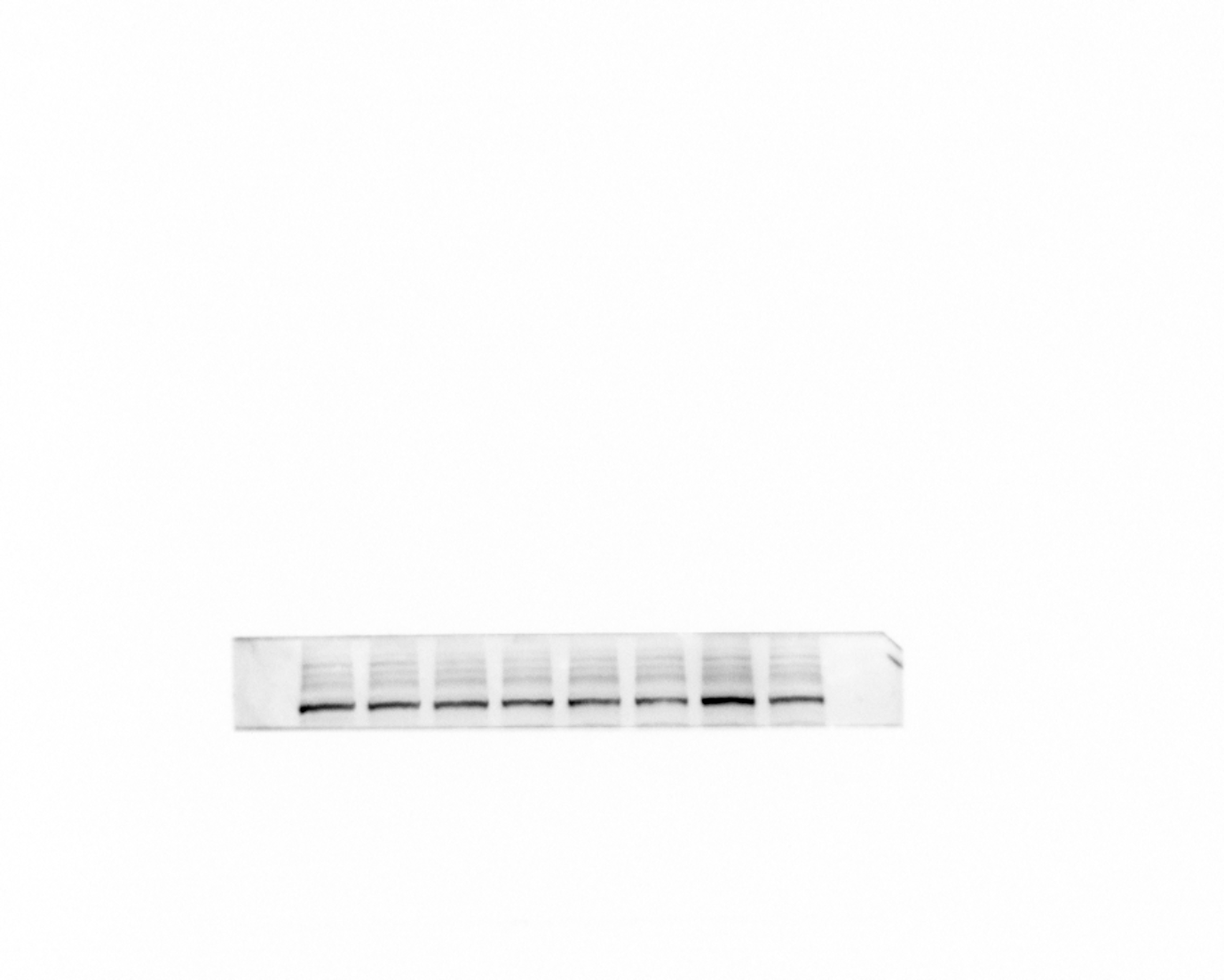

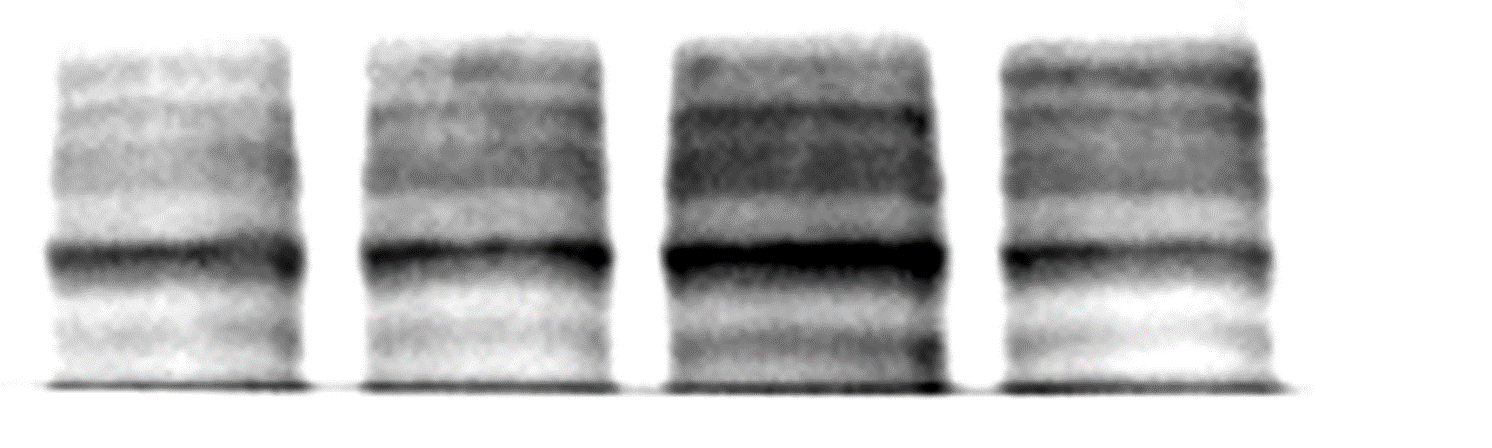

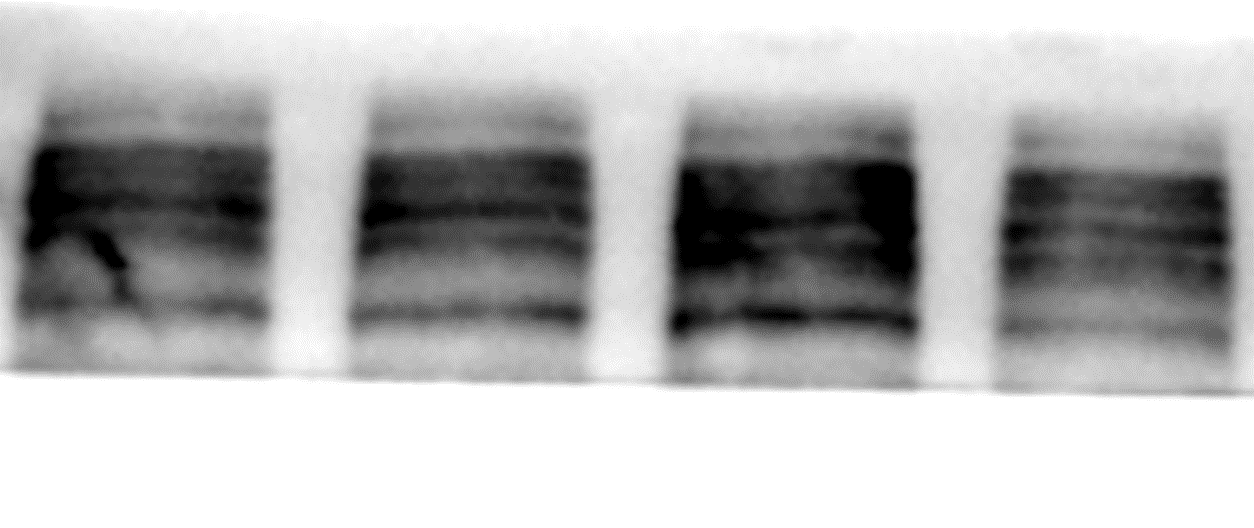


**pULK1**


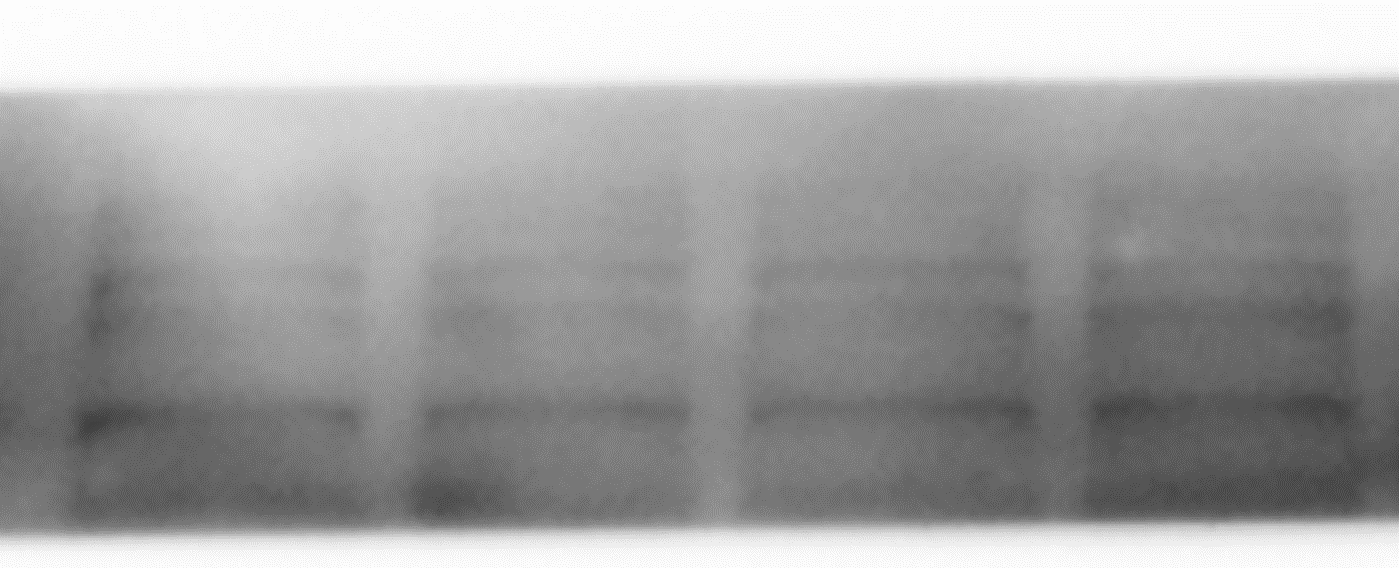

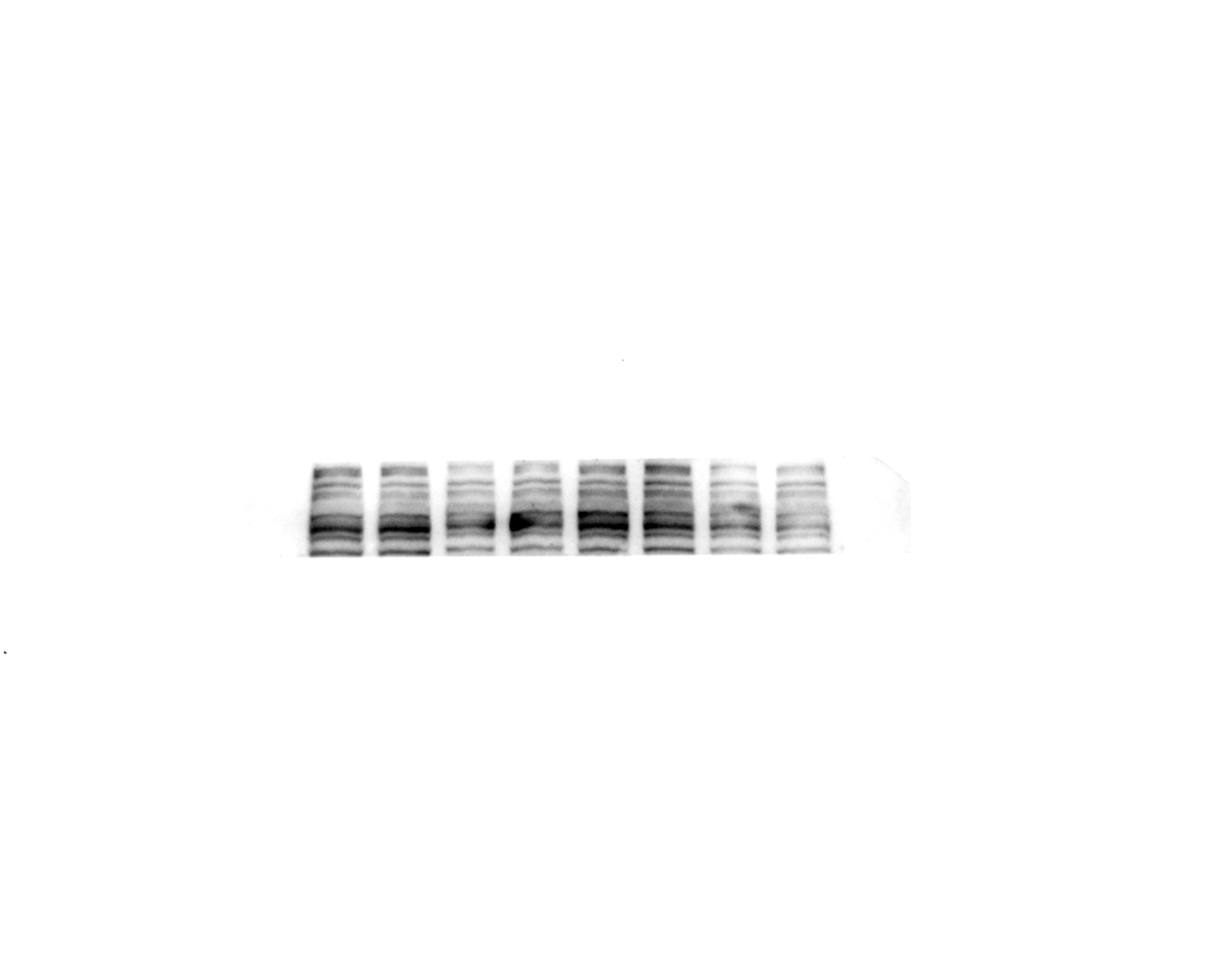

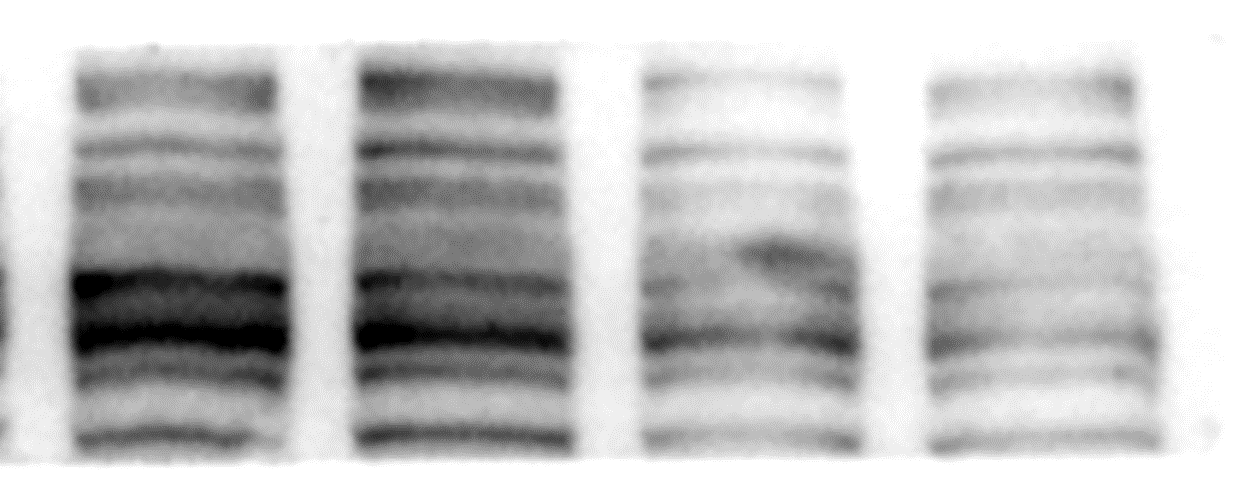


**ULK1**


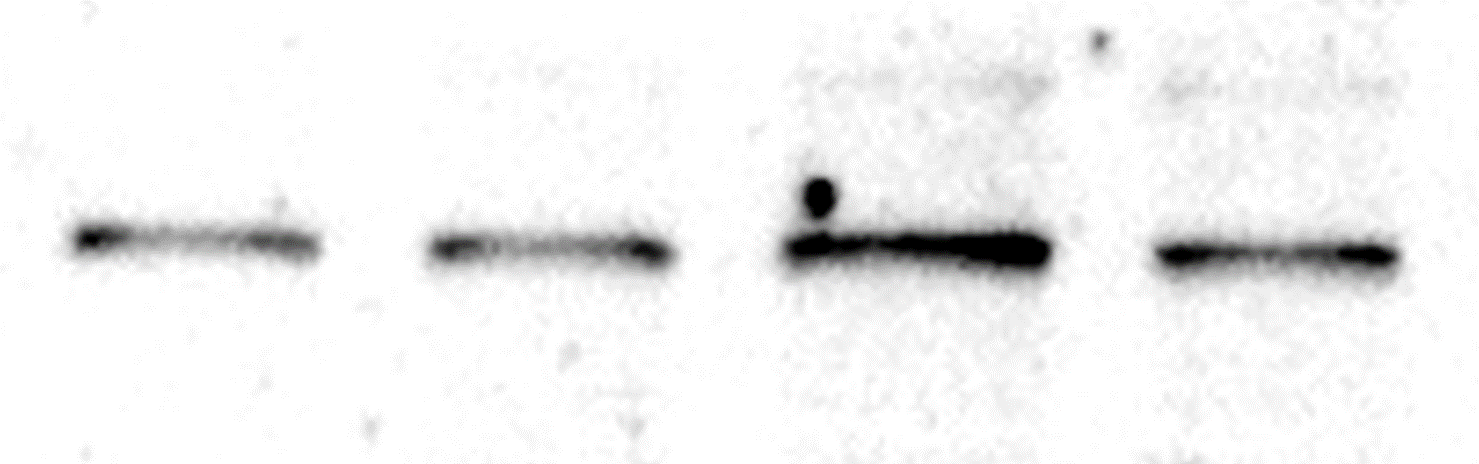

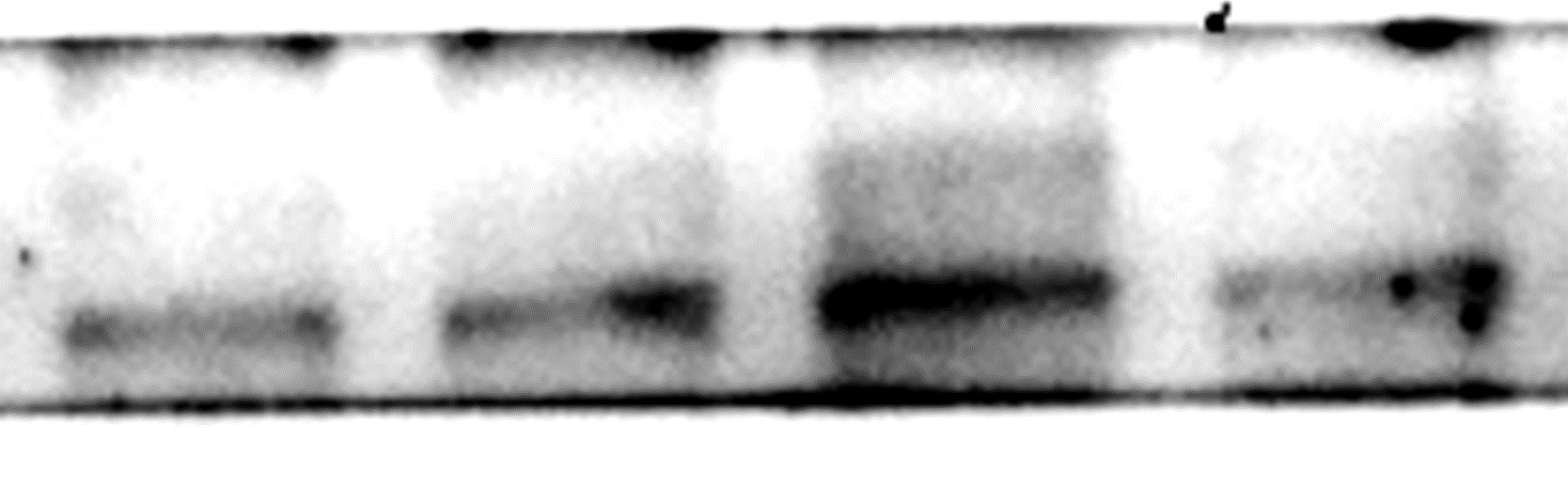

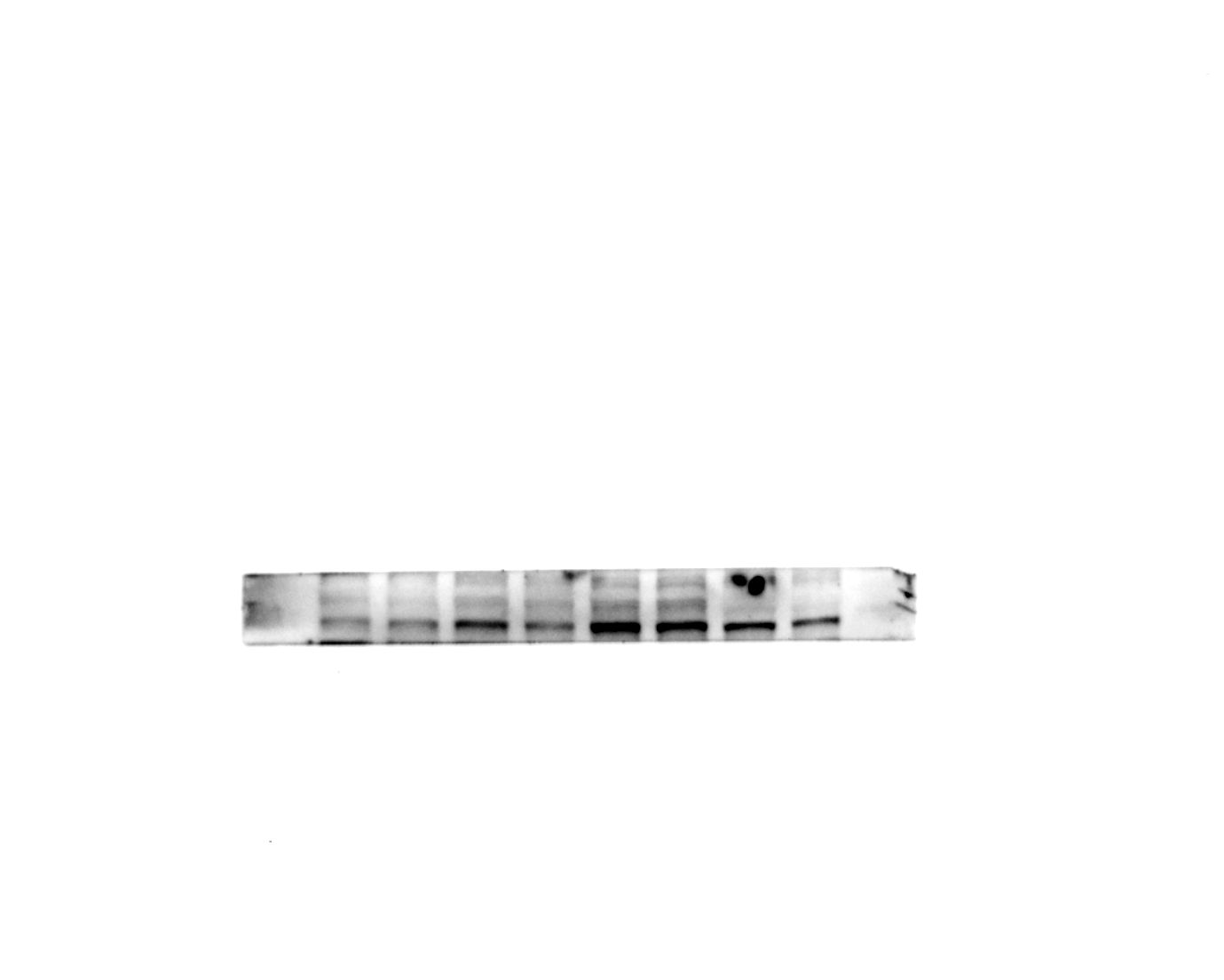


**PINK1**


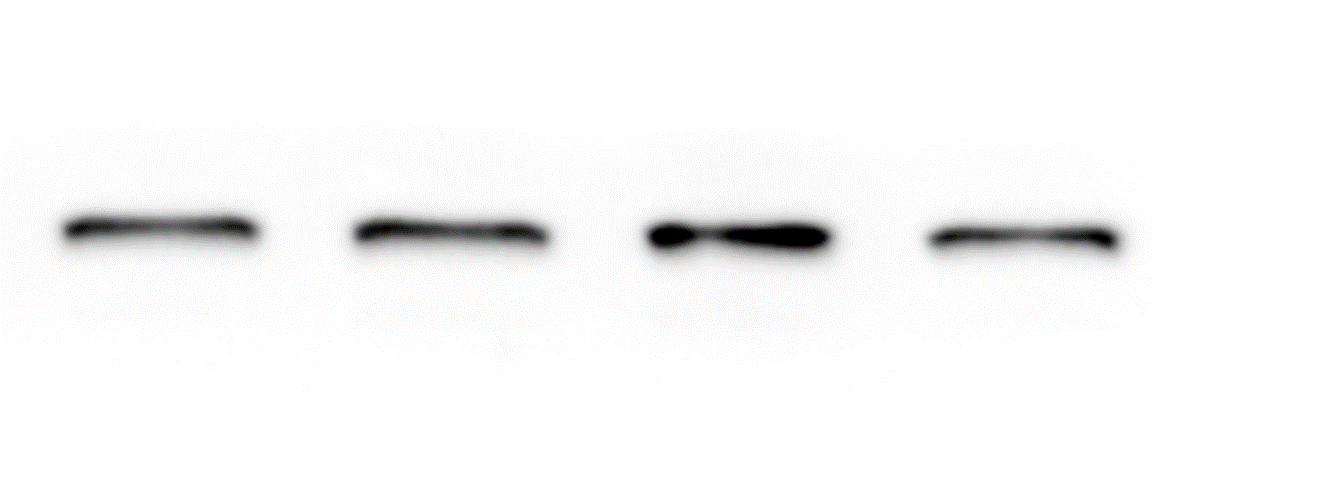


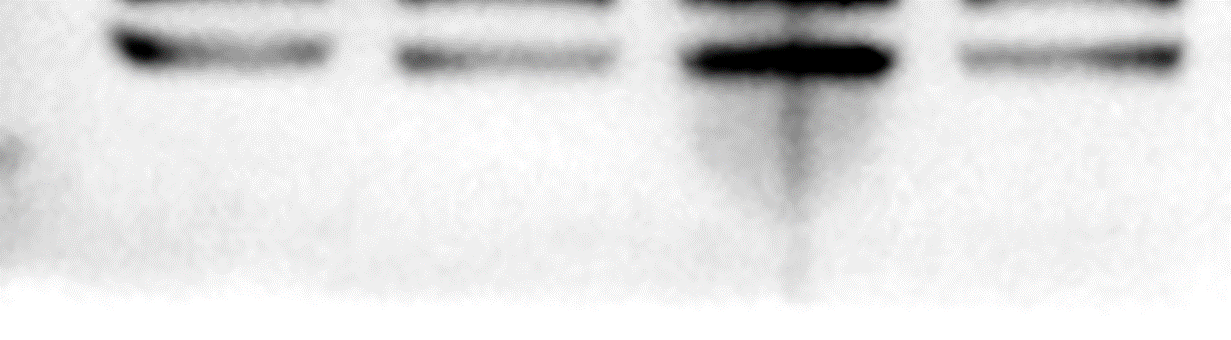

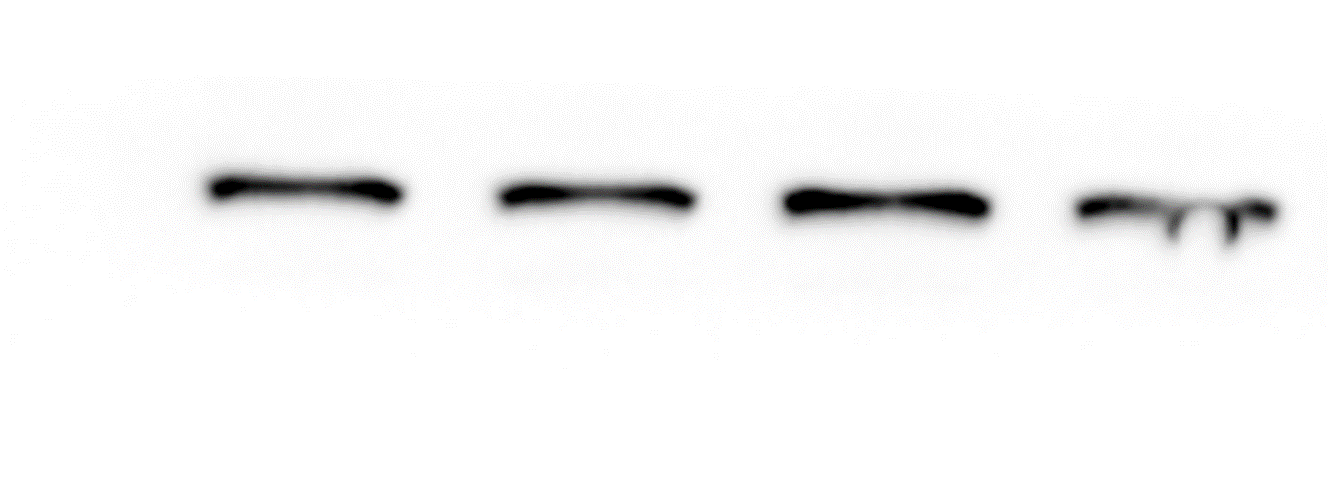

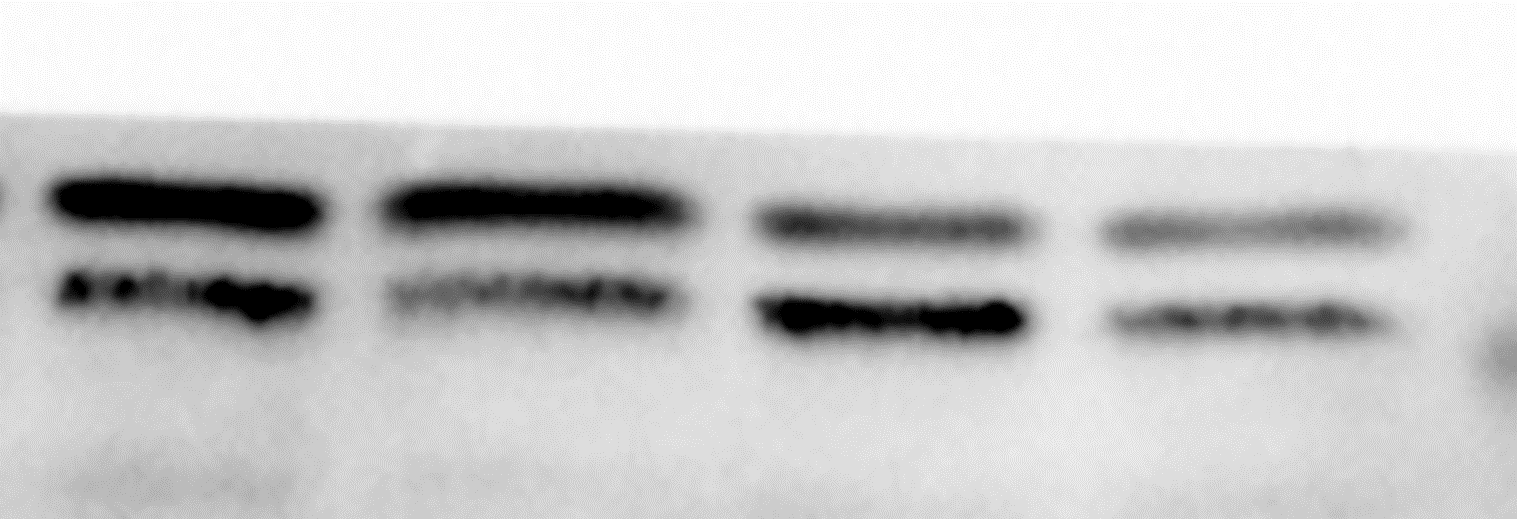

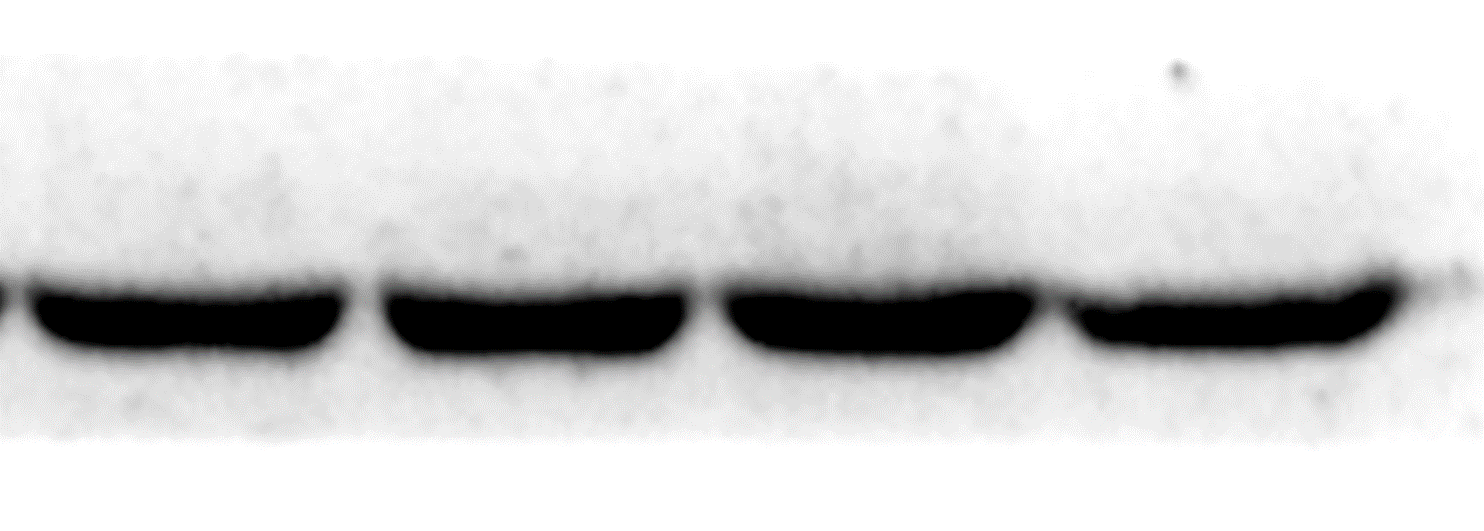


**Parkin**


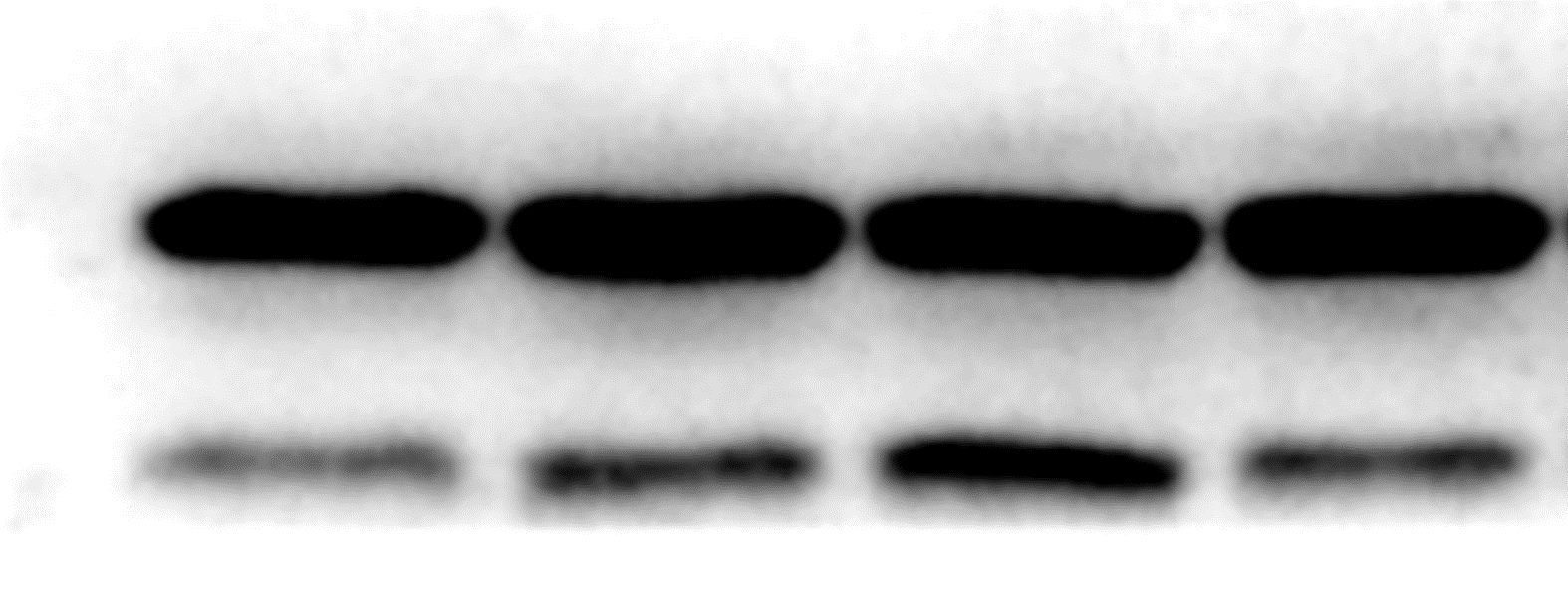

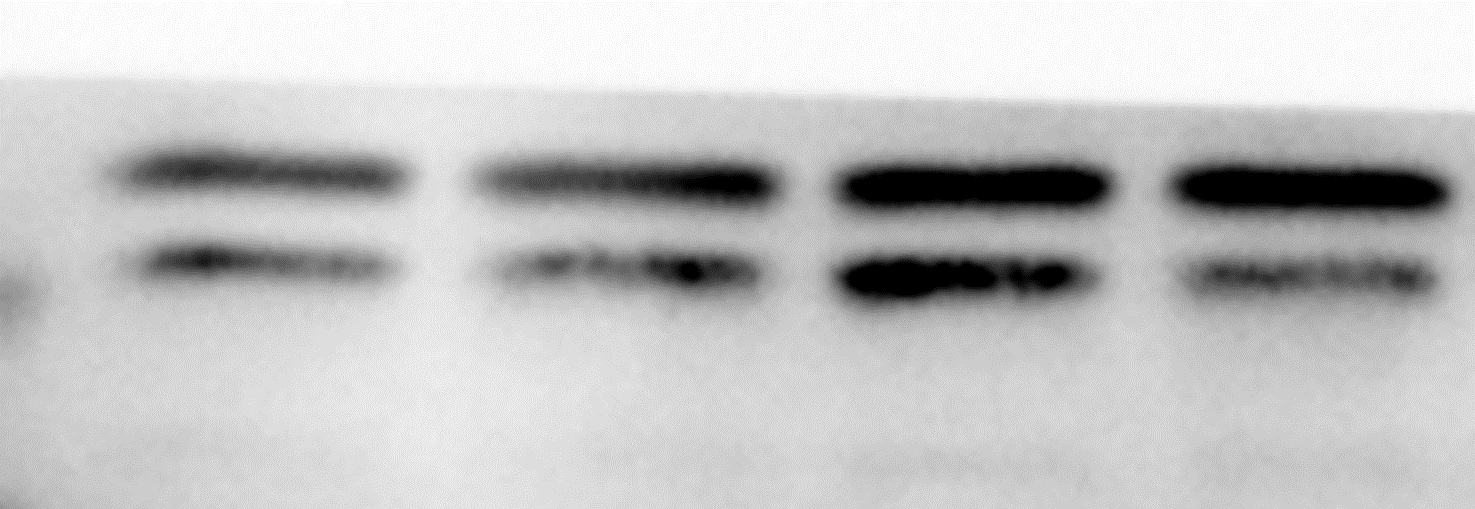


**LC3-II**

**LC3-I**

**p62**

**ACTB**

**Figure S7**

**Midbrain_Western blot**

**PS-NPs+Mel**

**Control**

**PS-NPs+Mel**

**PS-NPs**

**Mel**

**PS-NPs+Mel**

**PS-NPs**

**Mel**

**Control**

**PS-NPs**

**Mel**

**Control**

**pAMPK**

**AMPK**

**pULK1**

**ULK1**

**PINK1**

`

**Parkin**

**LC3-I**

**LC3-II**

**p62**

**ACTB**

**Figure S8C**

**Striatum_Western blot**

**PS-NPs+Mel**

**PS-NPs**

**Mel**

**Control**

**PS-NPs+Mel**

**PS-NPs+Mel**

**PS-NPs**

**Mel**

**Control**

**PS-NPs**

**Mel**

**Control**

**pAMPK**

**AMPK**

**pULK1**

**ULK1**

**PINK1**

**Parkin**

**LC3-II**

**LC3-I**

**p62**

**ACTB**
